# Supplementary material for: Autophagy modulates endothelial junctions to restrain neutrophil diapedesis during inflammation
Source: Immunity. 2021 Sep 14;54(9):1989–2004.e9. doi: 10.1016/j.immuni.2021.07.012 (PMC8459396; doi:10.1016/j.immuni.2021.07.012)
Supplement: Document S1. Figures S1–S6 [file mmc1.pdf]

Immunity, Volume *54*

Supplemental information

Autophagy modulates endothelial junctions to restrain neutrophil diapedesis during inflammation

Natalia Reglero-Real, Lorena Pérez-Gutiérrez, Azumi Yoshimura, Loïc Rolas, José Garrido-Mesa, Anna Barkaway, Catherine Pickworth, Rebeca S. Saleeb, Maria Gonzalez-Nuñez, Shani N. Austin-Williams, Dianne Cooper, Laura Vázquez-Martínez, Tao Fu, Giulia De Rossi, Matthew Golding, Mathieu-Benoit Voisin, Chantal M. Boulanger, Yoshiaki Kubota, William A. Muller, Sharon A. Tooze, Thomas D. Nightingale, Lucy Collinson, Mauro Perretti, Ezra Aksoy, and Sussan Nourshargh

Figure S1

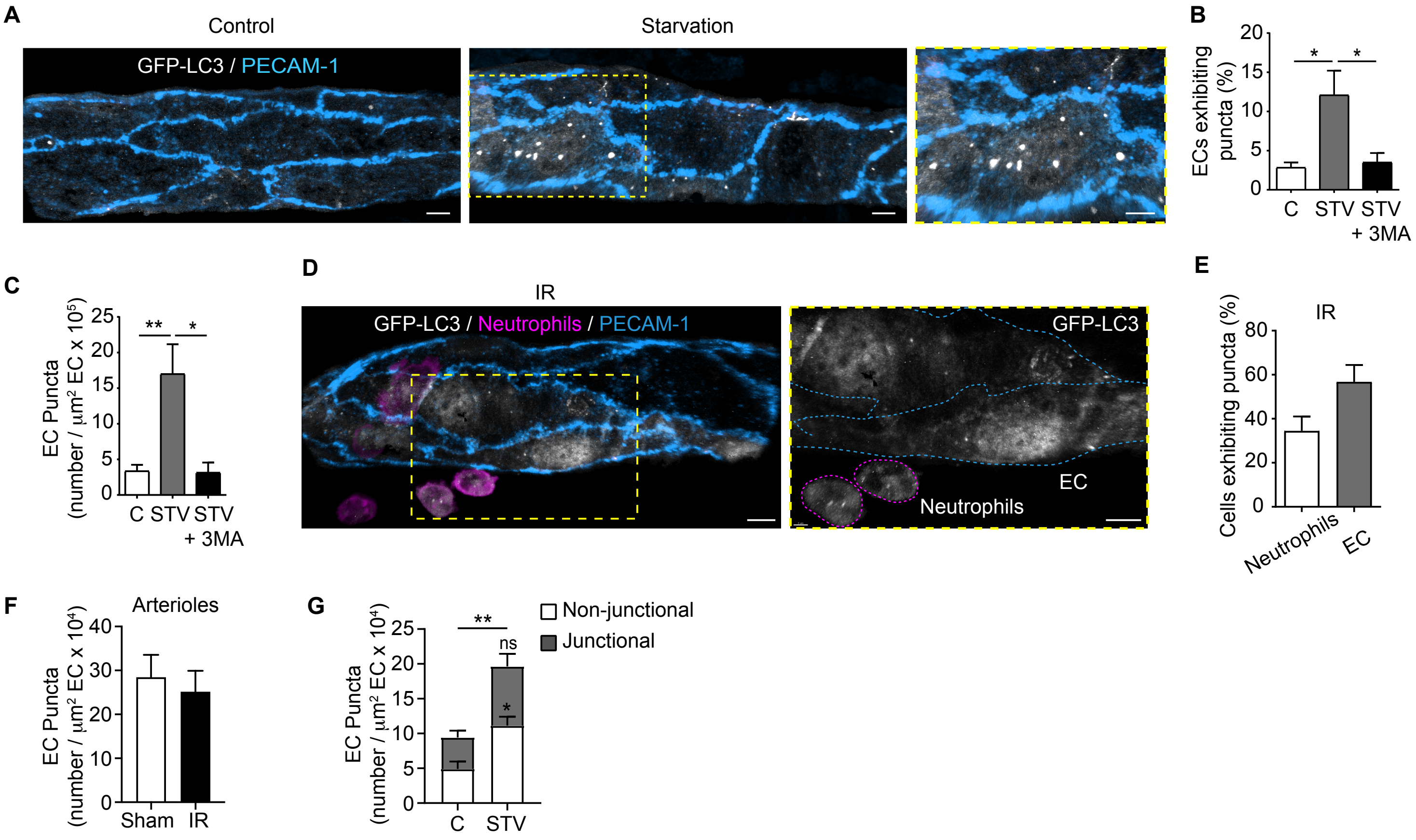

Figure S1 (Related to Figure 1). ECs exhibit GFP-LC3 puncta *in vivo* in response to starvation and inflammation. (**A-C**) *GFP-Map1lc3a$^{TG/+}$* mice were starved for 24 h. (**A**) Representative (n=7) confocal images of post-capillary venules (PCVs, PECAM-1) (scale bar 5 μm). (**B**) Percentage of venular ECs exhibiting puncta and (**C**) number of puncta per EC area in control and 3-MA-treated tissues, as quantified by confocal microscopy (n= 4-7 mice/group). (**D-F**) *GFP-Map1lc3a$^{TG/+}$* mice were subjected to local IR injury. (**D**) Representative confocal images (n=4) of PCVs (PECAM-1) stained for neutrophils (MRP14) (scale bar 5 μm). (**E**) Percentage of neutrophils and ECs exhibiting puncta (n=3-4 mice/group) and (**F**) quantification of GFP-LC3 puncta per arteriolar EC area, as assessed by confocal microscopy (n=3-6 mice/group). (**G**) *GFP-Map1lc3a$^{TG/+}$* mice were starved for 24 h and cremasteric PCVs analyzed for GFP-LC3 puncta localization by confocal microscopy (n=4-7 mice/group). Dashed boxes delineate magnified areas. Means ± SEM. Statistically significant difference from controls is shown by *P< 0.05 and **P< 0.01; ns, not significant.

Figure S2

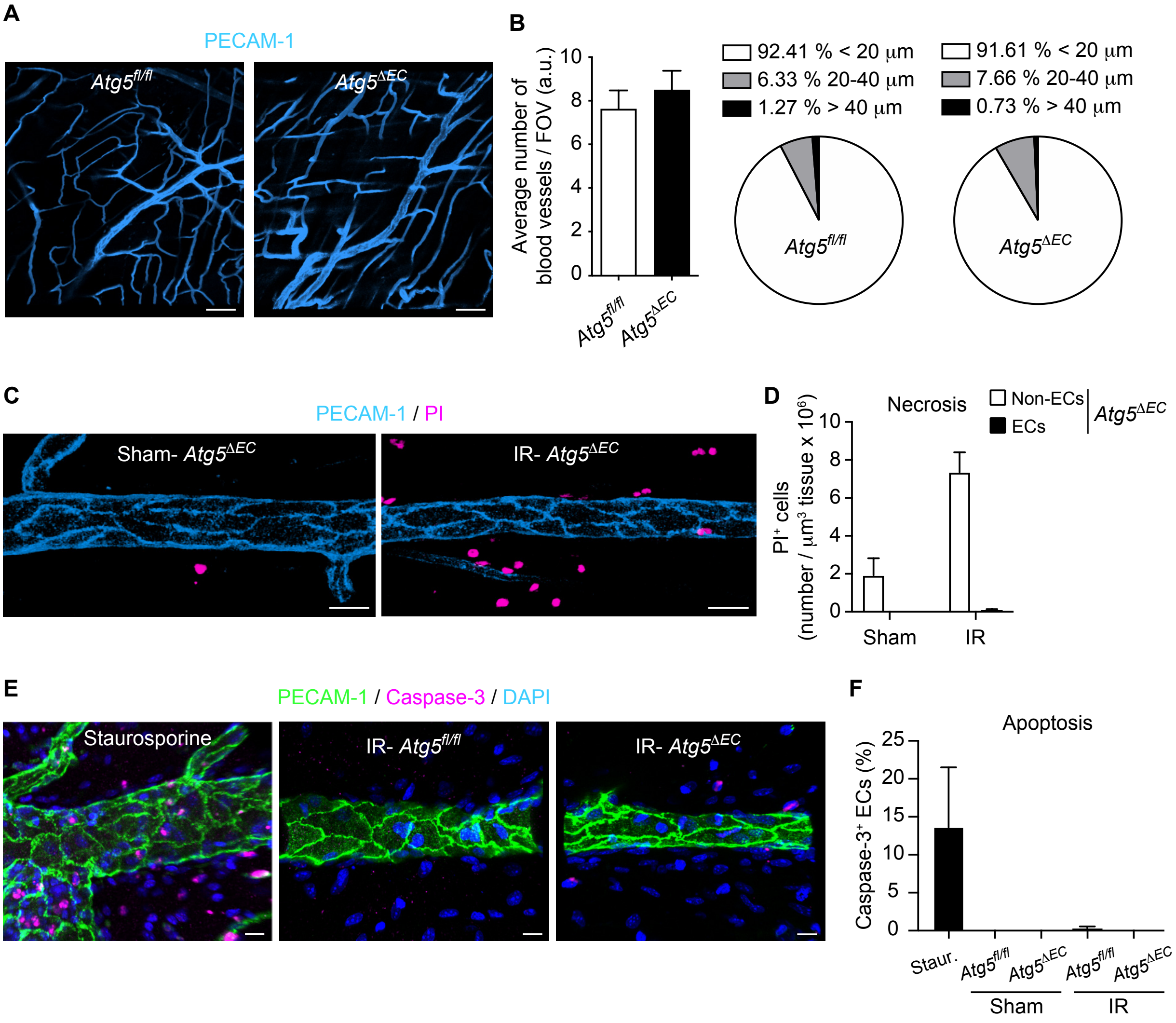

Figure S2 (Related to Figure 2). ATG5 deficient ECs are viable and healthy *in vivo*. (**A**) Representative confocal images (n=4) and (**B**) quantification of the number and size of blood vessels in post-capillary venules (PCVs) of *Atg5*$^{fl/fl}$ and *Atg5*$^{\Delta EC}$ mice, as quantified by confocal microscopy (n=4 mice/group). (**C-F**) *Atg5*$^{fl/fl}$ and *Atg5*$^{\Delta EC}$ mice were subjected to local IR injury. (**C**) Representative confocal images (n=4) of PCVs (PECAM-1) stained for propidium iodide (PI) and (**D**) quantification of necrotic cells (PI+) within *Atg5*$^{\Delta EC}$ mice, as quantified by confocal IVM (n=4 mice/group). (**E**) Representative confocal images (n=3) of PCVs (PECAM-1) stained for apoptotic cells (Caspase-3, scale bar 15 µm) and (**F**) percentage of Caspase-3 positive ECs, as assessed by confocal microscopy (n=3 mice/group). Means ± SEM.

Figure S3

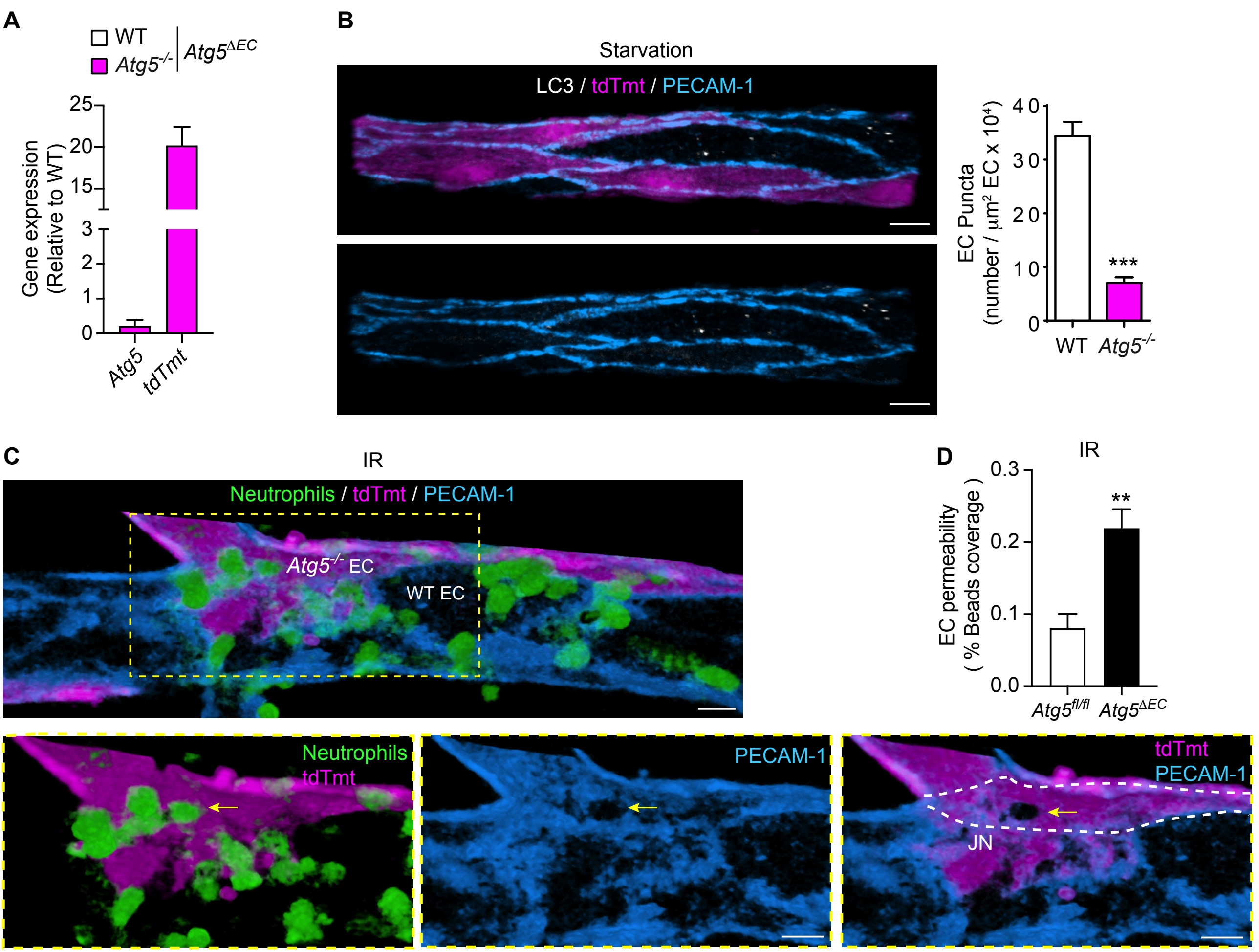

Figure S3 (Related to Figure 3). EC ATG5 deficiency inhibits LC3 puncta formation *in vivo* and supports neutrophil transcellular TEM. (**A**) Lung ECs of $Atg5^{\Delta EC}$ mice were analyzed for *Atg5* and *tdTomato* mRNA levels by RT-qPCR (n=3 mice/group). (**B**) Representative confocal images (n=3) of cremasteric post-capillary venules (PCVs) of starved $Atg5^{\Delta EC}$ mice stained for endogenous LC3, and associated quantification of LC3 puncta per venular EC area of $tdTomato^-$, wild-type (WT) ECs and $tdTomato^+$, $Atg5^{-/-}$ ECs (scale bar 7 µm, n=3 mice/group). (**C-D**) $Atg5^{fl/fl}$ and $Atg5^{\Delta EC}$ mice were subjected to local IR injury. (**C**) Representative confocal images (n=7) of a PCV supporting neutrophil transcellular TEM as shown through establishment of a pore in the body of an $Atg5^{-/-}$ ($tdTmt^+$) EC (arrow, scale bar 10 µm). (**D**) Quantification of EC permeability in $Atg5^{fl/fl}$ and $Atg5^{\Delta EC}$ mice, as assessed by confocal microscopy (n=3-4 mice/group). Dashed boxes delineate magnified areas. Means ± SEM. Statistically significant difference from controls is shown by $^{**}P< 0.01$ and $^{***}P< 0.001$.

Figure S4

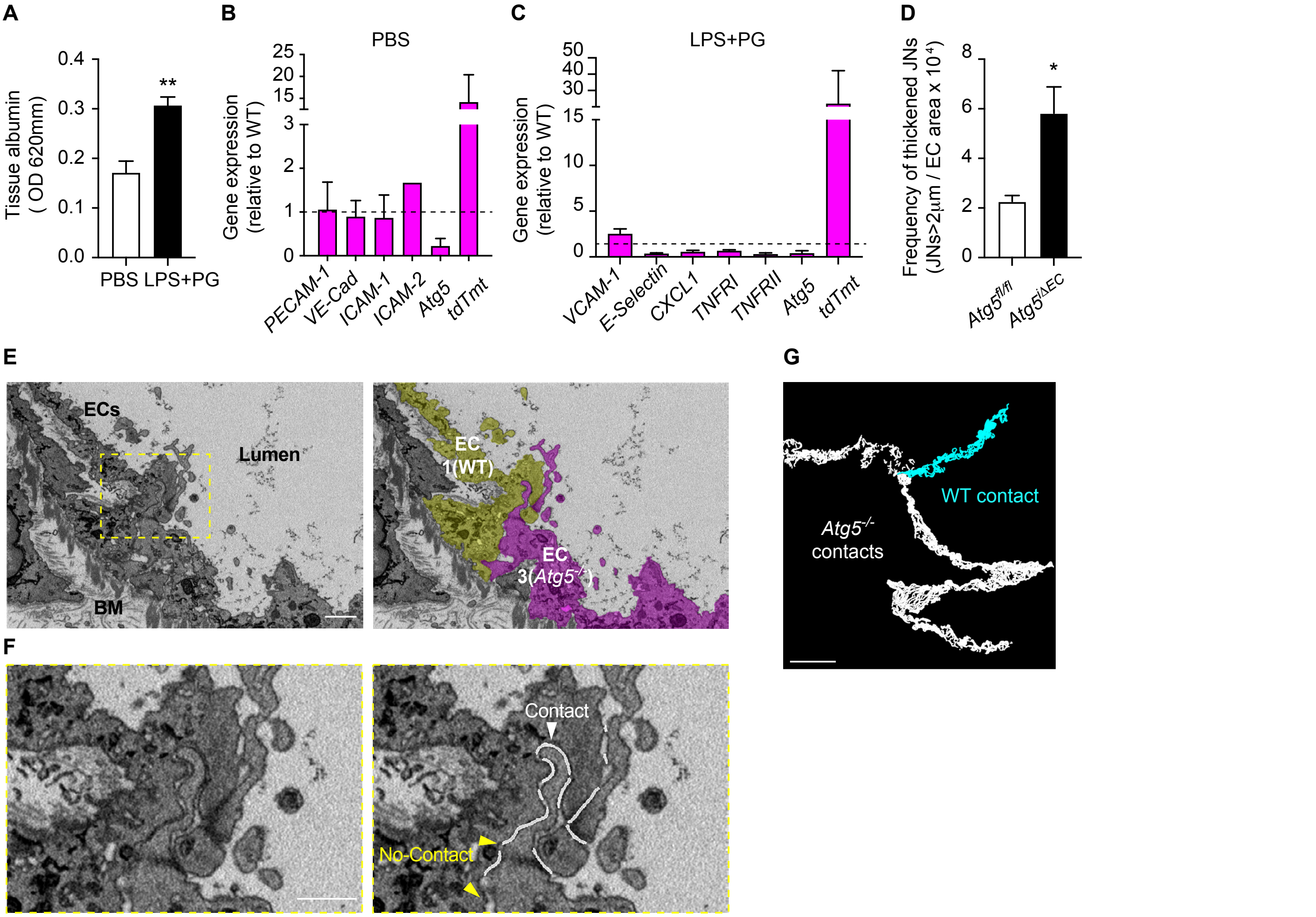

Figure S4 (Related to Figure 4). ATG5-dependent autophagy regulates the architecture and molecular composition of EC contacts. (**A-C**) *Atg5*$^{\Delta EC}$ mice were subjected to PBS or a model of endotoxemia (LPS+PG). (**A**) Quantification of lung vascular leakage, as assessed by local Evans blue extravasation (n=3 mice/group). mRNA levels under (**B**) basal and (**C**) endotoxemia conditions in lung ECs from *Atg5*$^{\Delta EC}$ mice, as assessed by RT-qPCR (n=3-5 mice/group). (**D**) Frequency of thickened junctions in post-capillary venules of IR-stimulated *Atg5*$^{i\Delta EC}$ mice, as quantified by confocal microscopy (n=3-4 mice/group). (**E-G**) One *Atg5*$^{\Delta EC}$ mouse was subjected to local IR injury. Electron micrographs of WT and *Atg5*$^{-/-}$ ECs shown in Figure 4J illustrating segmentation of (**E**) ECs and (**F**) cell-cell contacts (scale bars 1 μm and 0.5 μm, respectively). (**G**) 3D reconstruction of segmented cell-cell contacts following SBF-SEM of ECs depicted in Figure 4K (scale bar 5 μm). Dashed box delineates magnified area. Means ± SEM. Statistically significant difference from controls or between indicated groups is shown by *P< 0.05 and **P<0.01. BM, Basement membrane.

Figure S5

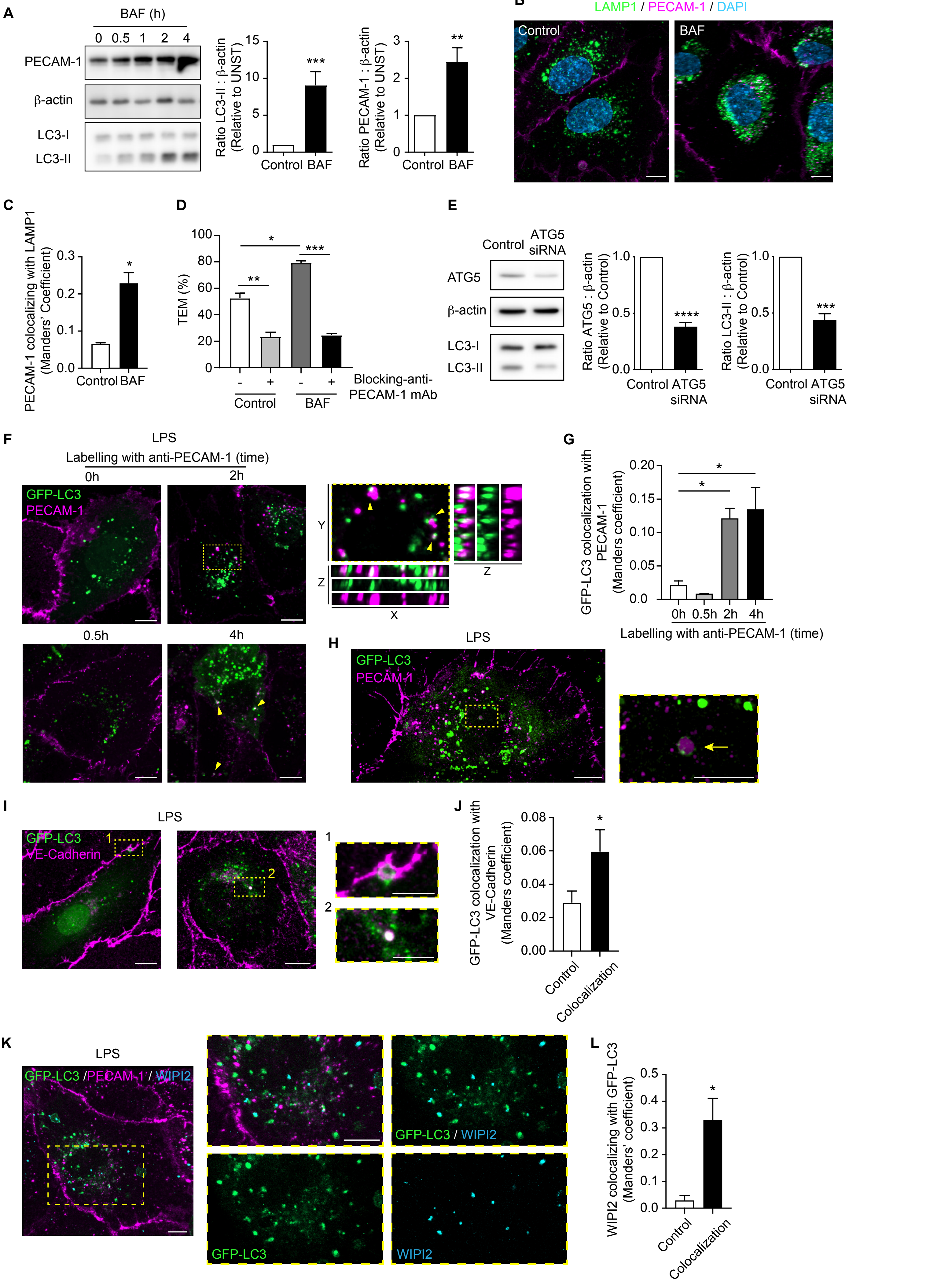

A
BAF (h)
0 0.5 1 2 4
PECAM-1
β-actin
LC3-I
LC3-II
Ratio LC3-II : β-actin (Relative to UNST)
Control BAF
***
Ratio PECAM-1 : β-actin (Relative to UNST)
**
B
LAMP1 / PECAM-1 / DAPI
Control
BAF
C
PECAM-1 colocalizing with LAMP1 (Manders' Coefficient)
Control BAF
D
TEM (%)
Blocking-anti-PECAM-1 mAb
Control
BAF
E
Control
ATG5 siRNA
ATG5
β-actin
LC3-I
LC3-II
Ratio ATG5 : β-actin (Relative to Control)
Ratio LC3-II : β-actin (Relative to Control)
****
***
F
LPS
Labelling with anti-PECAM-1 (time)
0h
2h
0.5h
4h
GFP-LC3
PECAM-1
G
GFP-LC3 colocalization with PECAM-1 (Manders coefficient)
0h 0.5h 2h 4h
Labelling with anti-PECAM-1 (time)
H
LPS
GFP-LC3
PECAM-1
I
LPS
GFP-LC3
VE-Cadherin
J
GFP-LC3 colocalization with VE-Cadherin (Manders coefficient)
Control
Colocalization
K
LPS
GFP-LC3 / PECAM-1 / WIPI2
GFP-LC3 / WIPI2
GFP-LC3
WIPI2
L
WIPI2 colocalizing with GFP-LC3 (Manders' coefficient)
Control
Colocalization

Figure S5 (Related to Figure 5). Bafilomycin A1 increases PECAM-1 protein levels and supports enhanced neutrophil TEM and internalized surface PECAM-1 localizes to GFP-LC3 positive compartments. (**A-D**) HUVECs were treated with bafilomycin A1 (BAF, 100 nM) for 4 h or the indicated times. (**A**) Immunoblot of total PECAM-1, LC3-I and LC3-II with β-actin acting as loading protein control and associated quantifications of fold increase of LC3-II and PECAM-1 relative to unstimulated conditions (n=11). (**B**) Representative confocal images (n=3) of HUVECs immunostained for PECAM-1 and LAMP1 and (**C**) associated quantification of PECAM-1 colocalization with LAMP1 (n=3, scale bars 10 μm). (**D**) Neutrophil TEM in the presence or absence of a blocking anti-PECAM-1 mAb (n=3). (**E**) Immunoblot of total ATG5 and LC3-II with β-actin acting as loading protein control for ATG5 silenced and control HUVECs and associated quantifications showing ATG5 and LC3-II fold change (n=6). (**F-L**) GFP-LC3 transfected HUVECs were stimulated with LPS for 4 h. (**F**) Representative confocal images (n=3-5) of GFP-LC3^{+}/ PECAM-1^{+} vesicles at the indicated times after incubation with anti-PECAM-1 mAb by antibody feeding (scale bars 10 μm); and (**G**) associated quantification of GFP-LC3 colocalization with PECAM-1, as quantified by Manders' coefficient (n=3-5; 40-100 cells analyzed per condition). (**H**) Representative super-resolution confocal image (n=1) of a GFP-LC3-associated vesicle containing PECAM-1 (arrow, scale bars 10 μm). (**I**) Representative confocal images (n=4) of GFP-LC3 transfected HUVECs immunostained for VE-Cadherin (scale bars 10 μm and enlargements 5 μm) and (**J**) quantification of GFP-LC3 colocalization with VE-Cadherin as compared to its offset control, as quantified by Manders' coefficient (n=4; >100 cells analyzed). (**K**) Representative confocal images (n=4) of GFP-LC3 transfected HUVECs immunostained for PECAM-1 and WIPI2, showing GFP-LC3^{+} and WIPI2^{+} vesicles (scale bar 10 μm) and (**L**) associated quantification of WIPI2 colocalization with GFP-LC3 as compared to its offset control, as quantified by Manders' coefficient (n=4; >100 cells analyzed). Dashed boxes delineate magnified areas. Means ± SEM. Statistically significant difference from controls or between indicated groups is shown by *P< 0.05, **P< 0.01, ***P< 0.001 and ****P<0.0001.

Figure S6

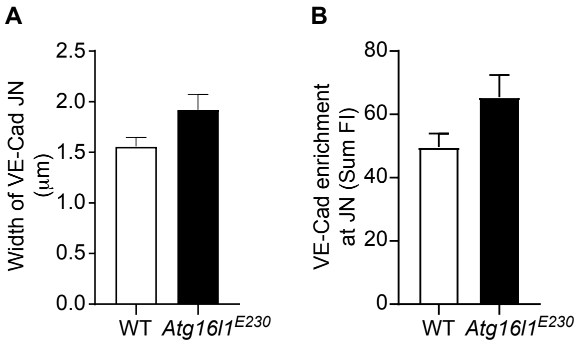

Figure S6 (Related to Figure 6). VE-Cadherin junctional expression in post-capillary venules from *Atg16L1^{E230}* mice. WT and *Atg16L1^{E230}* mice were subjected to local IR injury. Quantification of VE-Cadherin (**A**) junctional width and (**B**) protein enrichment, as assessed by confocal microscopy (n=3 mice/group; 12-19 vessels quantified per condition). Means ± SEM.
