# Supplementary material for: Autophagy modulates endothelial junctions to restrain neutrophil diapedesis during inflammation
Source: Immunity. 2021 Sep 14;54(9):1989–2004.e9. doi: 10.1016/j.immuni.2021.07.012 (PMC8459396; doi:10.1016/j.immuni.2021.07.012)
Supplement: Document S2. Article plus supplemental information [file mmc8.pdf]

Immunity

Article

Autophagy modulates endothelial junctions to restrain neutrophil diapedesis during inflammation

Graphical abstract

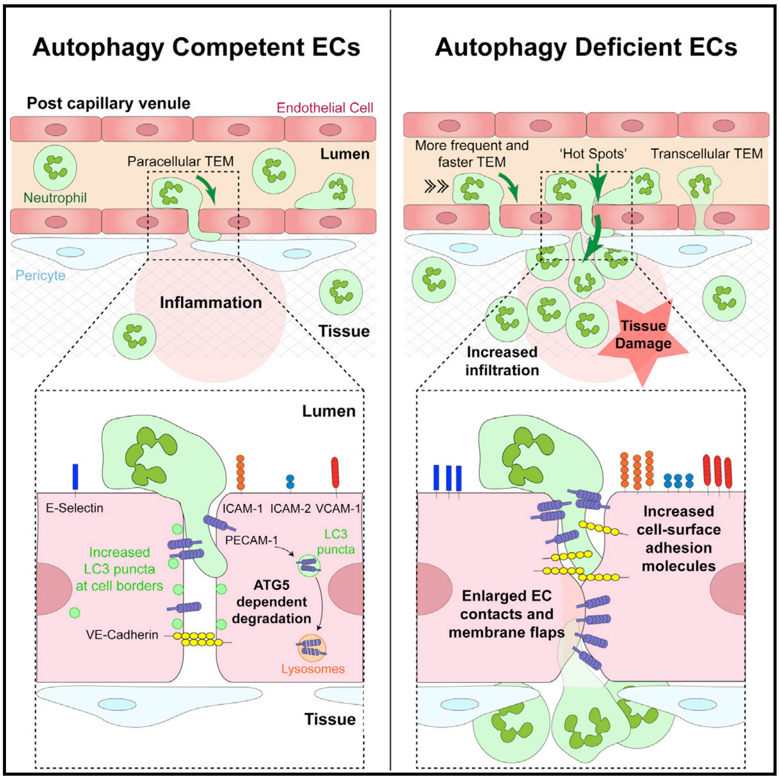

Highlights

- Inflamed venular ECs exhibit an autophagic response that localizes to EC contacts
- EC ATG5 deficiency promotes excessive and faster neutrophil TEM
- Ablation of EC autophagy increases cell surface expression of adhesion molecules
- Non-canonical autophagy operates in inflamed ECs and controls neutrophil migration

Authors

Natalia Reglero-Real, Lorena Pérez-Gutiérrez, Azumi Yoshimura, ..., Mauro Perretti, Ezra Aksoy, Sussan Nourshargh

Correspondence

n.reglero@qmul.ac.uk (N.R.-R.), s.nourshargh@qmul.ac.uk (S.N.)

In brief

Transendothelial cell migration (TEM) is a vital step in neutrophil infiltration of tissues, but the molecular basis of its cessation is unclear. Reglero-Real et al. show that in inflamed tissues, endothelial cell autophagy remodels junctional architecture and acts as a negative regulator of neutrophil TEM.

Reglero-Real et al., 2021, Immunity 54, 1989–2004
September 14, 2021 © 2021 The Author(s). Published by Elsevier Inc.
https://doi.org/10.1016/j.immuni.2021.07.012

CellPress

Immunity

CellPress
OPEN ACCESS

Article

Autophagy modulates endothelial junctions to restrain neutrophil diapedesis during inflammation

Natalia Reglero-Real,[1,10,*] Lorena Pérez-Gutiérrez,[1,10] Azumi Yoshimura,[2] Loïc Rolas,[1] José Garrido-Mesa,[3] Anna Barkaway,[1] Catherine Pickworth,[1] Rebeca S. Saleeb,[1] Maria Gonzalez-Nuñez,[3] Shani N. Austin-Williams,[3] Dianne Cooper,[3,9] Laura Vázquez-Martínez,[1] Tao Fu,[4] Giulia De Rossi,[5] Matthew Golding,[1] Mathieu-Benoit Voisin,[1] Chantal M. Boulanger,[6] Yoshiaki Kubota,[7] William A. Muller,[4] Sharon A. Tooze,[8] Thomas D. Nightingale,[1] Lucy Collinson,[2] Mauro Perretti,[3,9] Ezra Aksoy,[3] and Sussan Nourshargh[1,9,11,*]

[1]Centre for Microvascular Research, William Harvey Research Institute, Barts and the London School of Medicine and Dentistry, Queen Mary University of London, London EC1M 6BQ, UK
[2]Electron Microscopy Science Technology Platform, Francis Crick Institute, London NW1 1AT, UK
[3]Centre for Biochemical Pharmacology, William Harvey Research Institute, Barts and the London School of Medicine and Dentistry, Queen Mary University of London, London EC1M 6BQ, UK
[4]Department of Pathology, Feinberg School of Medicine, Northwestern University, Chicago, IL 60611, USA
[5]Department of Cell Biology, Institute of Ophthalmology, University College London, London EC1V9EL, UK
[6]Université de Paris, PARCC, INSERM, 75015 Paris, France
[7]Department of Anatomy, Keio University School of Medicine, Tokyo 113-0022, Japan
[8]Molecular Cell Biology of Autophagy Laboratory, Francis Crick Institute, London NW1 1AT, UK
[9]Centre for Inflammation and Therapeutic Innovation, Queen Mary University of London, London EC1M 6BQ, UK
[10]These authors contributed equally
[11]Lead contact
*Correspondence: n.reglero@qmul.ac.uk (N.R.-R.), s.nourshargh@qmul.ac.uk (S.N.)
https://doi.org/10.1016/j.immuni.2021.07.012

SUMMARY

The migration of neutrophils from the blood circulation to sites of infection or injury is a key immune response and requires the breaching of endothelial cells (ECs) that line the inner aspect of blood vessels. Unregulated neutrophil transendothelial cell migration (TEM) is pathogenic, but the molecular basis of its physiological termination remains unknown. Here, we demonstrated that ECs of venules in inflamed tissues exhibited a robust autophagic response that was aligned temporally with the peak of neutrophil trafficking and was strictly localized to EC contacts. Genetic ablation of EC autophagy led to excessive neutrophil TEM and uncontrolled leukocyte migration in murine inflammatory models, while pharmacological induction of autophagy suppressed neutrophil infiltration into tissues. Mechanistically, autophagy regulated the remodeling of EC junctions and expression of key EC adhesion molecules, facilitating their intracellular trafficking and degradation. Collectively, we have identified autophagy as a modulator of EC leukocyte trafficking machinery aimed at terminating physiological inflammation.

INTRODUCTION

The recruitment of neutrophils from the blood circulation to sites of injury and infection is a critical component of host defense and hence physiological inflammation. This innate immune reaction must be tightly regulated to avoid excessive tissue damage and the instigation of inflammatory diseases. To exit the vascular compartment, neutrophils are required to breach the endothelial cell (EC) monolayer that lines the lumen of all blood vessels (Nourshargh et al., 2010). Neutrophil migration through the endothelium is exquisitely regulated by the reorganization of junctionally expressed adhesion molecules such as platelet endothelial cell adhesion molecule-1 (PECAM-1), members of the junctional adhesion molecule (JAM) family, and VE-cadherin (Nourshargh and Alon, 2014; Reglero-Real et al., 2016; Vestweber, 2015), and is driven by locally generated and presented chemokines (Ley et al., 2007; Nourshargh and Alon, 2014; Girbl et al., 2018). Leukocyte transendothelial migration (TEM) commonly occurs via the movement of leukocytes across junctions between adjacent ECs (paracellular TEM), although migration through the body of the endothelium (transcellular TEM) also occurs *in vitro* and *in vivo* (Ley et al., 2007; Muller, 2011; Woodfin et al., 2011). In contrast to our in-depth knowledge of neutrophil TEM onset and progression, the molecular basis of neutrophil TEM cessation remains unclear.

Macroautophagy (hereafter called canonical autophagy) is an evolutionary conserved process that enables the delivery of cytoplasmic content to the lysosome for degradation. In recent years, canonical autophagy has emerged as a central regulator of innate immune functions, including cytokine production,

This is an open access article under the CC BY license (http://creativecommons.org/licenses/by/4.0/).

differentiation of immune cells, and pathogen clearance (Deretic and Levine, 2018; Yu et al., 2018; Deretic, 2021). Moreover, genome-wide studies have identified human autophagy-related (*Atg*) gene polymorphisms as markers of predisposition for multiple inflammatory disorders such as systemic lupus erythematosus (SLE), neutrophilic airway inflammation, and Crohn's disease (Bentham et al., 2015; Levine and Kroemer, 2019; Pham et al., 2016). While there is ample evidence of immune cell autophagy-related genes regulating inflammation, less is known about the role of EC autophagy in this context. However, autophagy is a homeostatic regulator of a number of EC functions, most notably EC survival and developmental angiogenesis, and is a key modulator of numerous vascular disorders (Nussenzweig et al., 2015; Vion et al., 2017; Sprott et al., 2019; Santovito et al., 2020). Mechanistically, the role of autophagy in these processes is linked to the exquisite capacity of the EC to sense local changes in physical and hemodynamic forces and cellular and molecular composition. Cellular stresses, including hypoxia, infection, and trauma, instigate causes of biological fluctuations in the EC milieu (Nourshargh and Alon, 2014), and activation of autophagy forms a key mechanism through which ECs respond to local perturbations (Verhoeven et al., 2021).

Despite the above, little is known regarding EC autophagy in relation to immune cell trafficking, most notably within the microcirculatory system. To address this fundamental issue, here, using high-resolution confocal microscopy, we investigated the role of EC autophagy in the neutrophil breaching of venular walls. We found that in acutely inflamed cremaster muscles, postcapillary venular ECs exhibited a profound autophagic response. This event was exclusively localized to EC borders and was aligned with the peak of neutrophil trafficking. Confocal intravital microscopy (IVM) revealed exaggerated and faster neutrophil TEM across autophagy-deficient ECs, while the pharmacological induction of autophagy inhibited neutrophil migration. Mechanistically, autophagic machinery regulated the expression of key EC adhesion molecules and modeling of EC contacts via the reorganization and degradation of junctional molecules. The WD40 domain of the autophagy-related protein ATG16L1, essential for non-canonical autophagy, played a role during this process. These findings reveal the ability of autophagy components to function in a non-canonical manner in inflamed ECs. Since the lack of EC autophagy led to excessive neutrophil infiltration in multiple inflammatory models, our results identify EC autophagy as an essential cellular process to limit physiological neutrophil trafficking.

RESULTS

Acutely inflamed microvascular venules exhibit induction of autophagy within EC junctions

Canonical autophagy involves the formation of dedicated double-membrane vesicles, commonly known as autophagosomes, that sequester autophagic cargos destined for cellular degradation and recycling processes (Ktistakis and Tooze, 2016). These organelles can be identified by their recruitment of the membrane-bound, phosphatidylethanolamine conjugated form of microtubule-associated protein light chain 3B (LC3) and are apparent as characteristic LC3^{+} puncta (Klionsky et al., 2016). To investigate the potential role of autophagy in relation to the temporal profile of neutrophil trafficking *in vivo*, we established a confocal microscopy method to observe and quantify the formation of LC3 puncta in cremasteric postcapillary venules (PCVs) (delineated with a non-function blocking anti-PECAM-1 monoclonal antibody [mAb]) using GFP-LC3 transgenic mice (*GFP-Map1lc3a*$^{TG/+}$) (Mizushima et al., 2004). Initial experiments detected increased numbers of GFP-LC3 puncta in venular ECs in response to nutrient starvation (Figures S1A–S1C), a potent stimulator of canonical autophagy. Local administration of the class III phosphatidylinositol 3-kinase (PI3K) inhibitor 3-methyladenine (3MA), an established blocker of starvation-induced autophagosome formation (Klionsky et al., 2016), significantly reduced the percentage of total ECs exhibiting GFP-LC3 puncta (Figure S1B) and the number of these organelles in ECs (Figure S1C). Next, we explored the prevalence of this reaction during the course of an acute inflammatory model of ischemia-reperfusion injury (IR; 40 min ischemia followed by reperfusion for up to 8 h). This reaction is characterized by a profound but transient infiltration of neutrophils during the reperfusion phase that peaks at 4 h and declines toward basal by 8 h post reperfusion (Figure 1A). In tissues subjected to IR (4 h reperfusion), transmigrated neutrophils exhibited GFP-LC3 puncta (Figures S1D and S1E), consistent with previous reports of a cell-autonomous role for autophagy in neutrophil functions (Bhattacharya et al., 2015; Riffelmacher et al., 2017). Following IR, arterial ECs showed no change in GFP-LC3 puncta numbers (Figure S1F), but venular ECs exhibited a robust increase in autophagic response (Figures 1B–1E, S1D, and S1E). Unlike the more diffuse distribution of puncta following starvation (Figures S1A and S1G), in IR-stimulated venules, this increase was entirely accounted for by the presence of GFP-LC3 puncta at PECAM-1^{+} EC contacts (Figures 1B and 1C). The quantification of endogenous LC3 puncta by immunofluorescence (IF) staining showed comparable results (Figure 1D), illustrating similar activities between GFP-LC3 and the wild-type (WT) protein in ECs. In investigating the time course of autophagy induction, GFP-LC3 puncta formation was only significant at the peak of the neutrophil emigration response, and declined toward control amounts during the cessation phase of neutrophil infiltration (Figure 1E). Lipopolysaccharide (LPS)-stimulated tissues (4 h), a reaction similarly characterized by neutrophil infiltration (Figure 1F), exhibited comparable EC junctional localization of GFP-LC3 puncta (Figures 1G and 1H). Overall, upon neutrophilic inflammatory reactions, ECs showed increased LC3 puncta at cell-cell contacts, suggesting that autophagic processes may play a role in regulating EC junctions, and hence immune cell infiltration into tissues.

EC ATG5 deficiency promotes increased and faster neutrophil TEM

Hypothesizing a causal link between EC autophagy and neutrophil trafficking, we sought to analyze neutrophil extravasation in mice with selective EC deficiency in the essential autophagy regulatory gene *Atg5* (Deretic and Levine, 2018; Ktistakis and Tooze, 2016). For this purpose, we used both constitutive (*Cdh5-cre;Atg5*$^{fl/fl}$ mice [*Atg5*$^{\Delta EC}$]) and inducible models of EC *Atg5* deletion (*Cdh5(BAC)-creER*^{T2}*; Rosa26*$^{tdTomato/tdTomato}$ [*Atg5*$^{i\Delta EC}$]). Since autophagy is an essential cellular survival mechanism, maintaining energy homeostasis through self-catabolic

CellPress
OPEN ACCESS

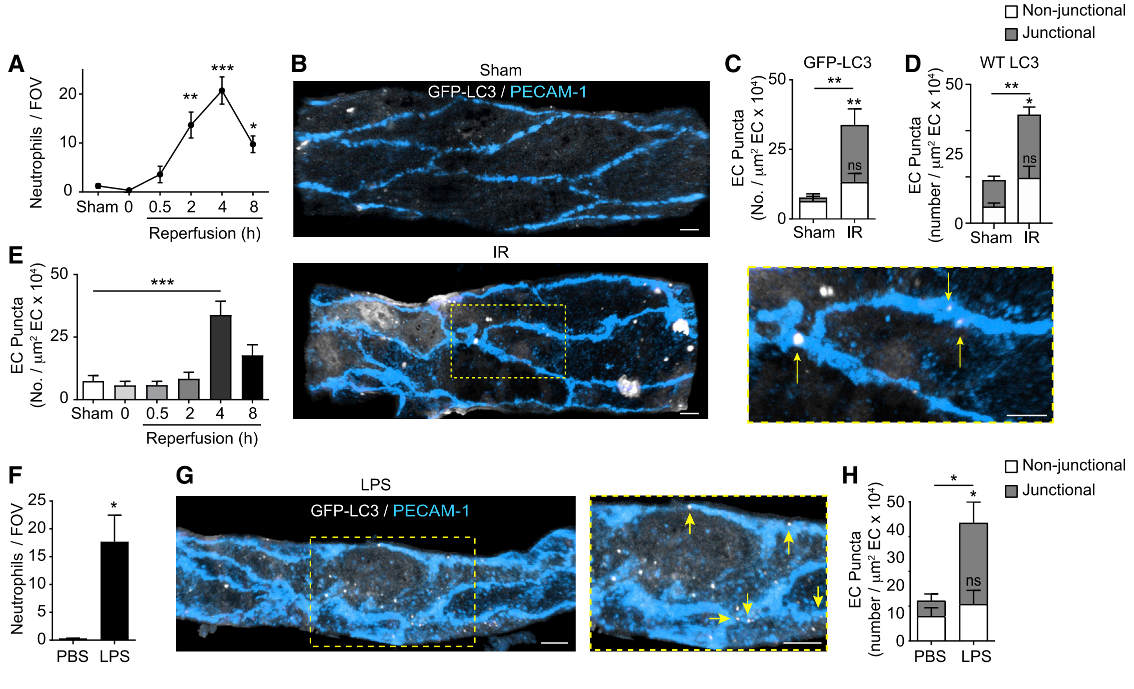

Figure 1. Acutely inflamed microvascular ECs exhibit induction of autophagy within vascular junctions

(A–E) *GFP-Map1lc3a*$^{TG/+}$ or WT mice were subjected to local IR injury

(A) Neutrophil extravasation at the indicated times post reperfusion, (n = 3–6 mice/group).

(B–E) Representative confocal images (n = 6) of postcapillary venules (PCVs, PECAM-1), with arrows indicating EC junctional localization of GFP-LC3 puncta (scale bar, 5 μm) (B) and quantification of (C) GFP-LC3 puncta or (D) endogenous LC3 puncta per venular EC area at 4 h and (E) at the indicated times post-reperfusion (n = 3–6 mice/group).

(F–H) *GFP-Map1lc3*$^{TG/+}$ mice were treated intrascrotally (i.s.) with PBS or LPS.

(F and G) Neutrophil extravasation (n = 3 mice/group) (F) and (G) representative (n = 3) confocal images of cremasteric PCVs (PECAM-1), with arrows indicating EC junctional localization of GFP-LC3 puncta (scale bar, 5 μm).

(H) Quantification of GFP-LC3 puncta per venular EC area (n = 3 mice/group). Dashed boxes delineate magnified areas.

Means ± SEMs. Statistically significant difference from controls or between indicated groups is shown by *$p < 0.05$, **$p < 0.01$, and ***$p < 0.001$; ns, not significant. See also Figure S1.

activity (Levine and Kroemer, 2019), we explored the health status of ATG5-deficient ECs *in vivo*. We observed comparable profiles of vascularity within the cremaster muscle microcirculation of *Atg5*$^{\Delta EC}$ mice as compared to their Cre^{-} littermate controls, *Atg5*$^{fl/fl}$ (Figures S2A and S2B). Furthermore, there was no evidence of enhanced EC necrosis or apoptosis in unstimulated, or in IR-stimulated, cremaster muscles of *Atg5*$^{\Delta EC}$ mice (Figures S2C–S2F). These results showed ATG5-deficient ECs to be viable and healthy in our experimental model.

We next evaluated the impact of EC autophagy deficiency on neutrophil TEM, initially continuing the work using the constitutive EC *Atg5* deletion model. Here, since the *Cdh5-cre* line is expressed in the embryonic hemogenic endothelium, which gives rise to recombined floxed alleles in ECs and hematopoietic lineages (Alva et al., 2006), we generated bone marrow chimeric mice to eliminate any potential impact of myeloid cell ATG5 deficiency on our readouts. To additionally provide a means of tracking neutrophil migration in real time, chimeric mice were generated by bone marrow transfer from *Lyz2-EGFP* genetically targeted mice (exhibiting EGFP^{hi} neutrophils) to irradiated *Atg5*$^{\Delta EC}$ and *Atg5*$^{fl/fl}$ mice. Analysis of total neutrophil infiltration in cremaster muscles of mice stimulated with local LPS or subjected to IR (4 h reperfusion) showed an increase in the infiltration of neutrophils in chimeric *Atg5*$^{\Delta EC}$ mice in comparison to chimeric *Atg5*$^{fl/fl}$ controls (Figures 2A–2C). Similarly, inducible EC autophagy-deficient mice, *Atg5*$^{i\Delta EC}$, exhibited an elevated neutrophil infiltration response following cremaster muscle IR (Figure 2D). In investigating the potential pathophysiological consequences of the intense neutrophil infiltration caused by EC autophagy deficiency, propidium iodide positivity revealed an elevated number of damaged cells in inflamed tissues of *Atg5*$^{\Delta EC}$ mice (Figure 2E). The propidium iodide-positive cells appeared to be predominantly tissue-infiltrated neutrophils, and there was no evidence of EC damage (Figures S2C and S2D). The latter confirmed the normal health status of ATG5-deficient ECs. These results indicated a negative regulatory role for EC autophagy in neutrophil infiltration into acutely inflamed tissues *in vivo*, providing a mechanism for protecting the host from excessive neutrophil-mediated tissue damage. To directly test this notion, and as a means of assessing a potential therapeutic strategy of controlling excessive neutrophil infiltration, we used a pharmacological approach to induce autophagy *in vivo*. Specifically, we used a cell-penetrant autophagy-inducing peptide, Tat-Beclin 1, that activates the master regulator of autophagy Beclin 1 (Peraro et al., 2017). Local application of Tat-Beclin 1, but not a scrambled control

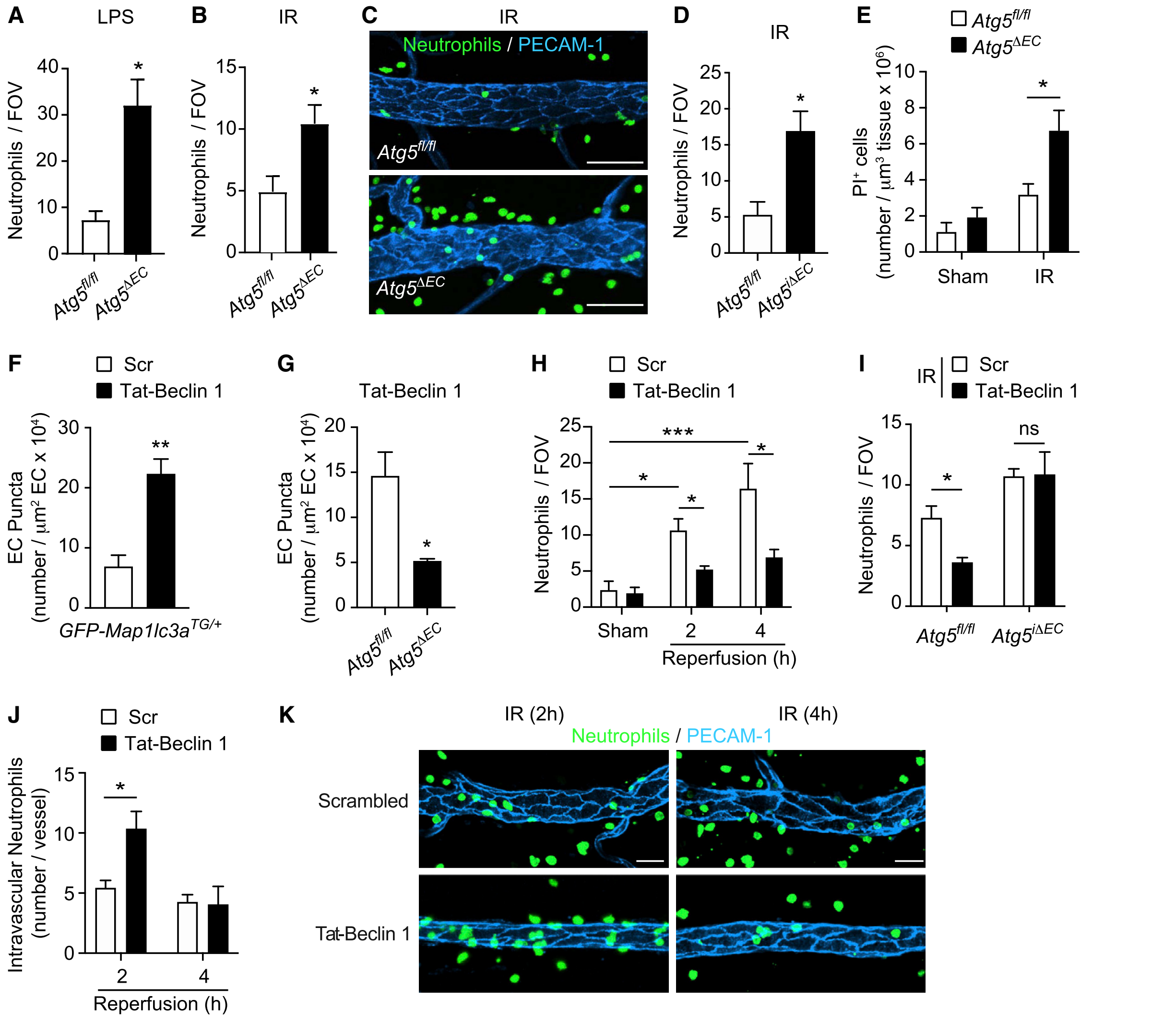

Figure 2. Modulation of EC autophagy controls neutrophil extravasation and cellular tissue damage

(A and B) Neutrophil extravasation in chimeric *Atg5*$^{fl/fl}$ and *Atg5*$^{\Delta EC}$ mice (A) treated i.s. with PBS or LPS and (B) subjected to local IR injury (n = 3–8 mice/group).

(C) Representative (n = 8) confocal images of IR-stimulated cremasteric PCVs (PECAM-1) immunostained for neutrophils (MRP14) (scale bar, 30 µm).

(D) Neutrophil extravasation in *Atg5*$^{fl/fl}$ and *Atg5*$^{i\Delta EC}$ mice subjected to local IR injury (n = 6 mice/group).

(E) Propidium iodide (PI)$^{+}$ cells in *Atg5*$^{fl/fl}$ and *Atg5*$^{\Delta EC}$ mice subjected to IR injury, as quantified by confocal IVM (n = 3–5 mice/group).

(F and G) GFP-LC3 puncta or endogenous LC3 puncta per venular EC area in cremasteric PCVs of (F) *GFP-Map1lc3*$^{TG/+}$ mice and (G) *Atg5*$^{fl/fl}$ and *Atg5*$^{\Delta EC}$ mice treated i.s. with scrambled or Tat-Beclin 1 peptide (n = 3–5 mice/group).

(H and I) Neutrophil extravasation in (H) WT mice and (I) *Atg5*$^{fl/fl}$ and *Atg5*$^{i\Delta EC}$ mice subjected to local IR injury and treated i.s. with scrambled or Tat-Beclin 1 (n = 4–6 mice/group).

(J and K) Intravascular neutrophils in WT mice subjected to local IR injury and treated i.s. with scrambled or Tat-Beclin 1 (n = 3–4 mice/group) (J), and (K) representative confocal images (n = 3–6) of cremasteric PCVs (PECAM-1) immunostained for neutrophils (MRP14) (scale bar, 30 µm).

Means ± SEMs. Statistically significant difference from controls or between indicated groups is shown by *p < 0.05, **p < 0.01, and ***p < 0.001; ns, not significant.

See also Figure S2.

peptide, induced GFP-LC3 and endogenous LC3 puncta in venular ECs in an ATG5-dependent manner (Figures 2F and 2G). This pharmacological strategy suppressed neutrophil infiltration in IR-stimulated cremaster muscles (Figures 2H), an effect that was abrogated in *Atg5*$^{i\Delta EC}$ mice (Figure 2I), showing the efficacy of Tat-Beclin 1 to be specific to EC autophagy induction. The suppression of neutrophil infiltration into tissues by Tat-Beclin 1 was aligned with the increased retention of neutrophils within the vascular lumen (Figures 2J and 2K), suggesting a regulatory role for EC autophagy in neutrophil breaching of ECs.

To delineate the stage of neutrophil trafficking regulated by EC ATG5, we used high-resolution confocal IVM (Girbl et al., 2018; Woodfin et al., 2011). Focusing on the inflammatory reaction induced by IR, we observed no difference in neutrophil adhesion to venular ECs between *Atg5*$^{fl/fl}$ and *Atg5*$^{\Delta EC}$ mice (Figure 3A). However, a significant increase (3-fold) in the number of neutrophil TEM events (Figure 3B), as well as a reduction

CellPress
OPEN ACCESS

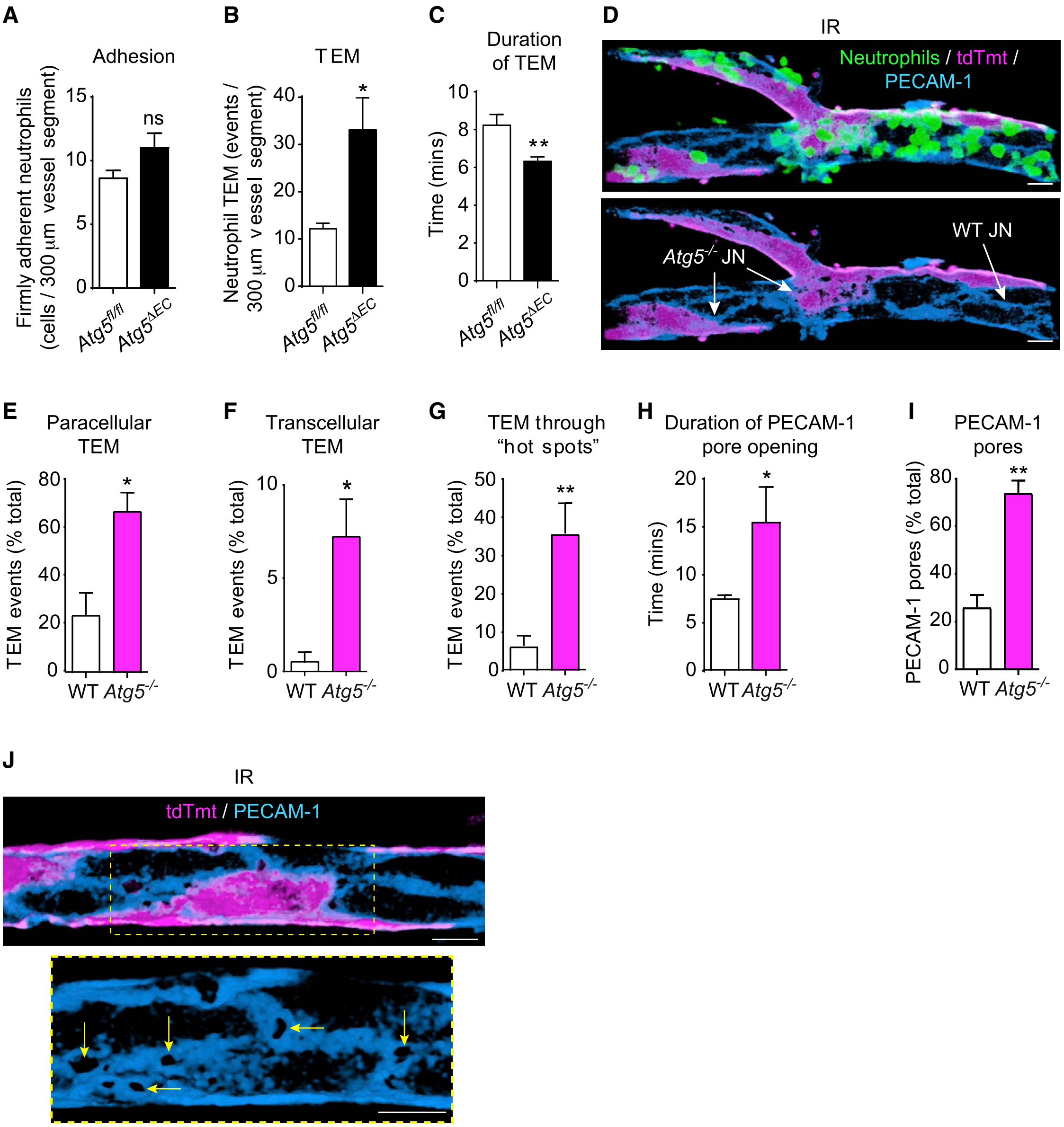

Figure 3. EC ATG5 deficiency promotes increased and faster neutrophil TEM

(A–C) Chimeric $Atg5^{fl/fl}$ and $Atg5^{\Delta EC}$ mice were subjected to local IR injury and neutrophil responses in cremasteric PCVs analyzed by confocal real-time IVM. Neutrophil (A) adhesion, (B) TEM, and (C) duration of TEM.

(D) Representative confocal images (n = 7) of cremasteric PCVs (PECAM-1) immunostained for neutrophils (MRP14), highlighting mosaic distribution of ECs, with arrows indicating $Atg5^{-/-}$ and WT junctions (scale bars, 15 μm).

(E–G) Neutrophil (E) paracellular and (F) transcellular TEM and (G) TEM through hotspots in WT and $Atg5^{-/-}$ junctions of chimeric $Atg5^{\Delta EC}$ mice.

(H and I) Pore (H) opening duration and (I) number within chimeric $Atg5^{\Delta EC}$ mice (n = 5–7 mice/group).

(J) Representative (n = 5) confocal images illustrating an $Atg5^{-/-}$ junction exhibiting multiple PECAM-1 pores (arrows; scale bars, 15 μm). Dashed boxes delineate magnified areas.

Means ± SEMs. Statistically significant difference from controls or between indicated groups is shown by *p < 0.05 and **p < 0.01; ns, not significant.

See also Figure S3.

in the duration of neutrophil TEM (Figure 3C), were detected in $Atg5^{\Delta EC}$ mice when compared to $Atg5^{fl/fl}$ littermates. As combining the Rosa-26-Tomato Cre reporter onto the $Atg5^{fl/fl}$ background provided a visual means of identifying ATG5-sufficient ECs (tdTomato$^-$; $Atg5^{fl/fl}$, hereafter referred to as WT) and ATG5-deficient counterparts (tdTomato$^+$; $Atg5^{-/-}$, hereafter referred to as $Atg5^{-/-}$) by confocal IVM (Figure 3D), we sought to take advantage of this phenomenon for our analysis. Specifically, noting a mosaic pattern of tdTomato expression (~40%) in PCVs of the cremaster muscle microcirculation (Figure 3D), we directly compared the profile and dynamics of neutrophil TEM events in relation to WT and $Atg5^{-/-}$ ECs within the

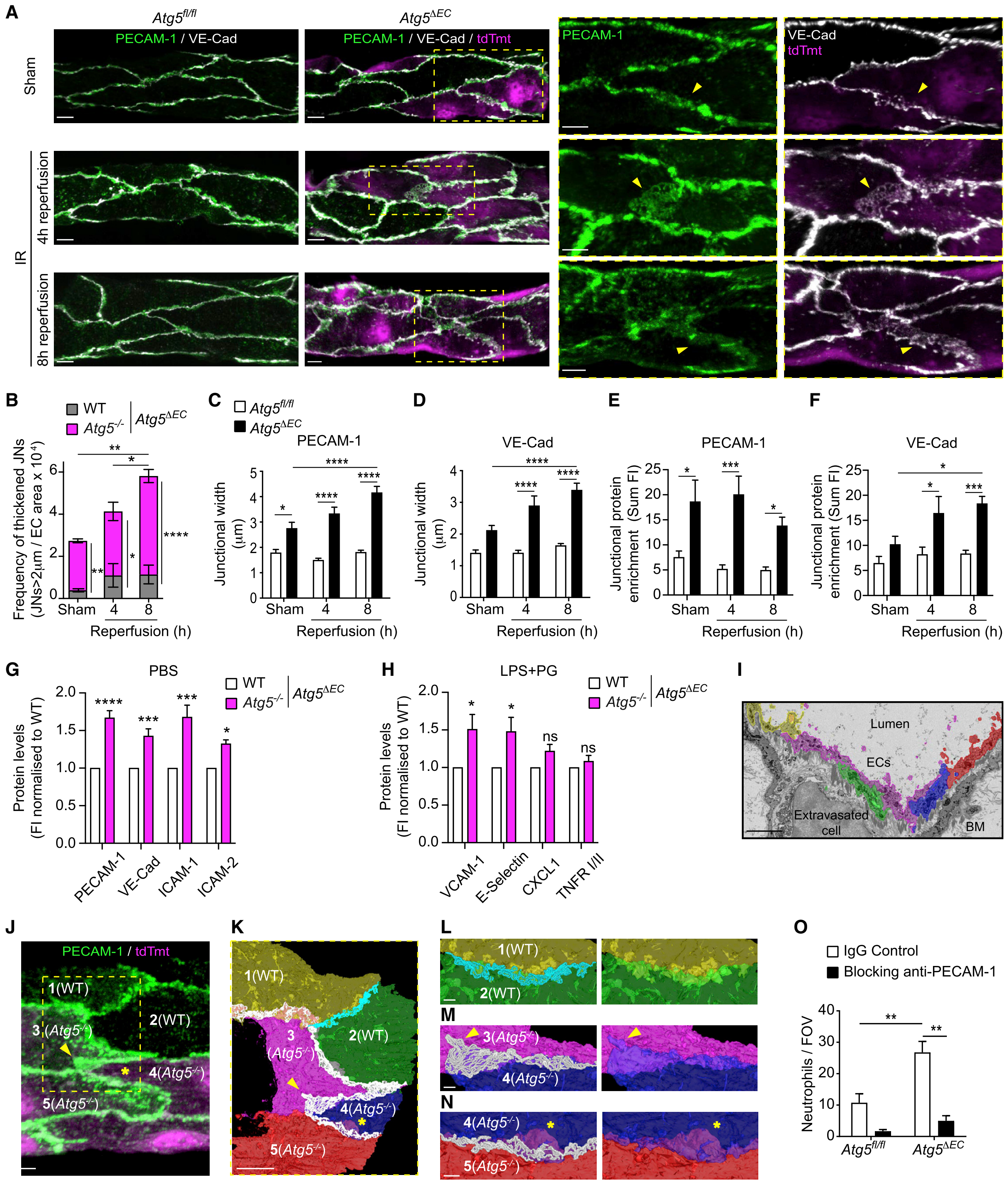

Figure 4. ATG5-dependent autophagy regulates the architecture and molecular composition of EC contacts

(A–F) *Atg5*$^{fl/fl}$ and *Atg5*$^{\Delta EC}$ mice were subjected to local IR injury.

(A and B) Representative confocal images (n = 3–4) of cremasteric PCVs (PECAM-1 and VE-cadherin) showing aberrant, thickened junctional structures (arrowheads) (A) and (B) frequency of thickened junctions (n = 3–4 mice/group).

(C–F) Quantification of PECAM-1 and VE-cadherin (C and D) junctional width and (E and F) junctional enrichment (n = 3 mice/group).

(G and H) Cell surface proteins under (G) basal and (H) endotoxemia conditions in WT and ATG5-deficient lung ECs from *Atg5*$^{\Delta EC}$ mice (n = 3–8 mice/group).

(legend continued on next page)

same venular segments. Importantly, the genetic status of the model was validated by quantifying *Atg5* and *tdTomato* mRNA in tdTomato$^+$ (Tmt$^+$) versus tdTomato$^-$ (Tmt$^-$) isolated lung ECs (Figure S3A) and by demonstrating the exclusive formation of LC3 puncta in Tmt$^-$ cremaster muscle ECs following starvation, as quantified by IF (Figure S3B). Since preliminary work showed a similar neutrophil TEM phenotype between WT-*Atg5*$^{-/-}$ and *Atg5*$^{-/-}$-*Atg5*$^{-/-}$ cellular contacts, to analyze neutrophil diapedesis, EC-EC junctions formed entirely by Tmt$^-$ ECs or those shared by at least one Tmt$^+$ EC were called WT and *Atg5*$^{-/-}$ junctions, respectively (Figure 3D). This approach revealed that higher numbers of neutrophils breached *Atg5*$^{-/-}$ junctions, predominantly in a paracellular mode, as compared to the frequency of neutrophils that penetrated WT junctions (Figure 3E). While WT ECs supported almost no incidence of neutrophil transcellular TEM, in line with our previous findings (Woodfin et al., 2011), *Atg5*$^{-/-}$ ECs showed a significant increase in neutrophil transcellular TEM (~8-fold greater; Figures 3F and S3C). Although leukocyte transcellular TEM across inflamed brain ECs is prevalent (Engelhardt and Ransohoff, 2012), our findings offer EC ATG5 deficiency as a basis for transcellular neutrophil TEM in the peripheral circulation. In agreement with the higher frequency of neutrophil TEM events across *Atg5*$^{-/-}$ junctions, autophagy-deficient junctions exhibited distinct hotspots wherein the junctions under investigation supported multiple sequential neutrophil TEM responses (Figure 3G; Video S1). This phenomenon, which included at times up to 8 neutrophil TEM occurrences through the same pore (Video S2), and the prolonged duration of pore openings (Figure 3H), were associated with a significant increase in the number of PECAM-1 pores created at *Atg5*$^{-/-}$ junctions (Figures 3I and 3J). The latter suggests that within the same venule, neutrophils were 3 times more likely to go through an *Atg5*$^{-/-}$ junction than a WT junction. These distinct regions, with a high capacity to support neutrophil TEM, presented unusually enlarged PECAM-1-labeled areas (Figure 3J), demonstrating the existence of aberrant EC protein amounts and localization within autophagy-deficient junctions. *Atg5*$^{\Delta EC}$ mice, in comparison to *Atg5*$^{fl/fl}$ littermates, also exhibited enhanced vascular permeability, as assessed by the leakage of intravenously (i.v.)-injected fluorescent beads (20 nm in diameter) (Figure S3D), further indicating the aberrant functionality of autophagy-deficient EC junctions. Collectively, neutrophils exhibited exaggerated and faster migration across ATG5-deficient venular endothelium, revealing EC autophagy as an effective modulator of neutrophil TEM dynamics.

ATG5-dependent autophagy regulates the architecture and molecular composition of EC contacts

To explore the junctional phenotype of *Atg5*$^{-/-}$ cells in greater detail, we analyzed the expression of two integral EC junctional components, VE-cadherin and PECAM-1 (Dejana et al., 2009; Muller, 2016; Reglero-Real et al., 2016), in control and inflamed cremasteric microcirculation of *Atg5*$^{\Delta EC}$ and *Atg5*$^{fl/fl}$ mice by IF. In *Atg5*$^{fl/fl}$ mice, control sham-treated muscles and tissues subjected to IR (4 and 8 h post reperfusion) showed the characteristic defined junctional expressions of VE-cadherin and PECAM-1 (Figure 4A). In contrast, the tissues of *Atg5*$^{\Delta EC}$ mice exhibited distinctly thickened junctions, typified by the enrichment of VE-cadherin and PECAM-1 (Figure 4A). These thickened EC contact sites were aligned exclusively with *Atg5*$^{-/-}$ junctions and were commonly >2 μm in width, as compared to an average of ~1 μm in *Atg5*$^{fl/fl}$ mice and WT junctions of *Atg5*$^{\Delta EC}$ mice (Figures 4B–4D). Furthermore, the frequency and size of aberrant *Atg5*$^{-/-}$ junctional regions increased with the reperfusion time (Figures 4B–4D) and expressed ~2- to 4-fold more immunoreactive PECAM-1 and VE-cadherin as compared to junctions in *Atg5*$^{fl/fl}$ mice (Figures 4E and 4F). In aiming to investigate this phenomenon in a different vascular bed, we analyzed the expression of multiple leukocyte trafficking molecules on lung ECs (LECs) isolated from untreated *Atg5*$^{\Delta EC}$ mice or *Atg5*$^{\Delta EC}$ mice subjected to a model of endotoxemia characterized by enhanced lung vascular permeability (Figure S4A). Here, we found increased cell-surface protein amounts of adhesion molecules PECAM-1, VE-cadherin, intercellular adhesion molecule-1 (ICAM-1), ICAM-2, vascular cell adhesion molecule-1 (VCAM-1), and E-selectin in *Atg5*$^{-/-}$ (Tmt$^+$) as compared to WT (Tmt$^-$) ECs (Figures 4G and 4H). However, *Atg5*$^{-/-}$ LECs showed no difference in mRNA expression of the molecules (apart from VCAM-1) under basal or inflammatory conditions (Figures S4B and S4C). These results rule out differential transcriptional regulation as a dominant cause of the enhanced protein detected. Stimulated WT and *Atg5*$^{-/-}$ LECs exhibited similar amounts of the chemokine C-X-C motif chemokine ligand 1 (CXCL1) and tumor necrosis factor (TNF) receptors I and II (Figure 4H), suggesting selective regulation of integral membrane leukocyte trafficking machinery by EC ATG5. In addition, *Atg5*$^{i\Delta EC}$ mice recapitulated the aberrant junctional phenotype observed in constitutive *Atg5*$^{\Delta EC}$ upon IR (Figure S4D).

To gain insight into potential changes in the architecture of ATG5-deficient EC contacts at the ultrastructural level, we applied correlative light and electron microscopy (CLEM) to analyze IR-stimulated tissues of *Atg5*$^{\Delta EC}$ mice. For this purpose,

(I–N) Correlative light electron microscopy (CLEM) analysis of a venular segment in an IR-stimulated *Atg5*$^{\Delta EC}$ mouse (n = 1).

(I) Serial-block face scanning electron microscopy (SBF-SEM) micrograph of the region of interest (ROI) (targeted as shown in Video S3), illustrating segmentation of ECs.

(J) Confocal image showing WT and *Atg5*$^{-/-}$ ECs within the ROI, with the latter exhibiting thickened PECAM-1 junctional structures (arrowhead and asterisk).

(K) 3D reconstruction of segmented ECs and cell-cell contacts of the venular area depicted in (J).

(L–N) Enlargements of the 3D model illustrating (L) WT-WT and (M and N) *Atg5*$^{-/-}$-*Atg5*$^{-/-}$ cell contacts showing areas of enlarged contacts (arrowhead) and membrane flaps (asterisk, scale bars, 1 μm).

(O) Neutrophil extravasation in *Atg5*$^{fl/fl}$ and *Atg5*$^{\Delta EC}$ mice subjected to IR injury and treated with an isotype control or anti-PECAM-1-blocking mAb (n = 3–4 mice/group). Dashed boxes delineate magnified areas.

Means ± SEMs. Statistically significant difference from controls or between indicated groups is shown by *p < 0.05, **p < 0.01, ***p < 0.001, and ****p < 0.0001. Scale bars, 5 μm, unless otherwise specified.

See also Figure S4.

we implemented a method for aligning confocal microscopy and microscopic X-ray computed tomography (micro-CT) imaging to locate regions of interest (ROIs) within EM-processed samples (see Method details; Video S3). With this approach, we analyzed a PCV incorporating 2 WT (cells 1 and 2) and 3 *Atg5*$^{-/-}$ ECs (cells 3, 4, and 5) (Video S3). Imaging of this ROI by serial-block face scanning electron microscopy (SBF-SEM), followed by segmentation of individual ECs (Figures 4I and S4E; Video S4) and cell-cell contacts (Figure S4F), generated a three-dimensional (3D) model that correlated PECAM-1 IF staining with apposed lateral plasma membranes of adjacent ECs (Figures 4J and 4K; Video S5). The latter is in agreement with previous EM findings (Feng et al., 2004). The contacts formed between ECs, as defined by the maximum distance that enables PECAM-1 homophilic interactions ($\leq$25 nm) (Jiang et al., 2016), were enlarged between *Atg5*$^{-/-}$- *Atg5*$^{-/-}$ and WT-*Atg5*$^{-/-}$ cells as compared to WT-WT contacts (Figures 4K–4M and S4G). Furthermore, the images revealed *Atg5*$^{-/-}$ ECs exhibiting PECAM-1-enriched non-contact sites that appeared as sheet-like plasma membrane projections or flaps in close proximity to cell-cell contacts, representing regions of cellular overlap (Figure 4N; Video S5). This ultrastructural analysis identified *Atg5*$^{-/-}$ junctions as enlarged and potentially more accessible regions for neutrophils to breach, presenting a mechanism for the observed enhanced neutrophil TEM. Notably, the treatment of mice with a blocking anti-PECAM-1 mAb abrogated neutrophil infiltration in both control and *Atg5*$^{\Delta EC}$ mice (Figure 4O). These data support the notion that accumulated proteins at *Atg5*$^{-/-}$ contacts are accessible to migrating neutrophils and facilitate exaggerated neutrophil trafficking across ATG5-deficient venular ECs. Collectively, ATG5-deficient ECs exhibited aberrant molecular and cellular features that can account for excessive neutrophil trafficking across autophagy-deficient endothelium.

EC autophagy machinery regulates PECAM-1 intracellular trafficking and degradation

Having found that defective EC autophagy leads to the accumulation of cell surface molecules, most notably junctional proteins, we next investigated the role of the autophagy machinery in their dynamics and turnover. Because we showed that neutrophil trafficking in *Atg5*$^{\Delta EC}$ mice is PECAM-1 dependent (Figure 4O), we focused on this molecule under conditions of autophagy deficiency as an illustration of endothelial leukocyte-trafficking machinery. Initially using an *in vitro* approach, we analyzed PECAM-1 expression in human umbilical vein ECs (HUVECs) treated with the vacuolar H^+ ATPase inhibitor bafilomycin A1 that prevents the fusion between autophagosomes and lysosomes. Within this model, while autophagic degradation activity (autophagic flux) was suppressed, as indicated by LC3-II accumulation, PECAM-1 total protein and co-localization with lysosomal-associated membrane protein-1 (LAMP-1) were enhanced (Figures S5A–S5C). The latter indicated that PECAM-1 traffics into LAMP-1$^+$ compartments destined for degradation. Bafilomycin A1 treatment of HUVECs also resulted in increased neutrophil TEM in a PECAM-1-dependent manner (Figure S5D), in line with the *in vivo* data acquired from *Atg5*$^{\Delta EC}$ mice (Figures 2A, 2B, 3B, and 4O). Next, we small interfering RNA (siRNA) silenced ATG5 in HUVECs, a strategy that reduced ATG5 protein expression and LC3-II accumulation by ~60% (Figure S5E). To model an acute inflammatory scenario, we examined LPS (4 h)-stimulated control and ATG5-silenced HUVECs for the dynamics and degradation kinetics of cell surface PECAM-1 post-internalization through pulse-chase experiments (Figure 5A). Here, by labeling endogenous cell surface PECAM-1 with cell-impermeant sulfo-NHS-biotin and then chasing this pool over time, we noted that the biotinylated PECAM-1 fraction degraded more rapidly in control than in ATG5-silenced HUVECs (Figures 5B and 5C). As a complementary approach, we labeled GFP-LC3-transfected HUVECS with a non-blocking AF555-anti-PECAM-1 mAb, a strategy that distinctly delineated junctionally localized PECAM-1 (Figure S5F, upper left panel). After 0.5 h of this immunolabeling, the antibody internalized and began to distribute to intracellular vesicles (Figure S5F, lower left panel). At 2 and 4 h postantibody labeling, a fraction of GFP-LC3 vesicles (17% and 20%, respectively) directly co-localized with PECAM-1 (Figures 5D, S5F, and S5G). Furthermore, super-resolution and live cell confocal imaging demonstrated the recruitment of GFP-LC3 to PECAM-1$^+$ vesicles in real time (Figures 5E and S5H; Video S6). Likewise, LPS-stimulated ECs revealed partial co-localization of GFP-LC3 vesicles with VE-cadherin (Figures S5I and S5J). Since ATG5 is mechanistically linked to diverse membrane trafficking pathways (Ktistakis and Tooze, 2016), to investigate the role of canonical autophagy in PECAM-1 trafficking, we tracked the recruitment of the ATG16L1-interacting protein WIPI2 (WD repeat domain, phosphoinositide-interacting protein 2) to GFP-LC3$^+$-PECAM-1$^+$ compartments. Immunostaining of fixed GFP-LC3-transfected HUVECs with an anti-WIPI2 mAb revealed WIPI2 co-localization with GFP-LC3 vesicles (Figures S5K and S5L). This is consistent with the role of WIPI2 in LC3 recruitment to developing autophagosomes (Dooley et al., 2014). However, the majority of GFP-LC3$^+$-PECAM-1$^+$ vesicles (>90%) were devoid of WIPI2 staining (Figures 5F and 5G), suggesting that the targeting of LC3 to PECAM-1$^+$ compartments is principally mediated by ATG5-dependent non-canonical autophagy mechanisms. Collectively, cell-surface PECAM-1 distributes to LC3$^+$ compartments and is partially degraded in an ATG5-dependent manner in stimulated ECs.

Non-canonical autophagy operates in microvascular ECs and regulates PECAM-1 distribution in inflamed tissues

A growing body of evidence suggests that, in addition to canonical autophagy, other trafficking pathways that use a subset of ATG proteins can result in the conjugation of LC3 to non-autophagosome compartments independently of WIPI2 (Ktistakis and Tooze, 2016; Martinez et al., 2016; Martinez-Martin et al., 2017). Since we found no evidence for WIPI2 recruitment to GFP-LC3$^+$-PECAM-1$^+$ compartments in HUVECs (Figures 5F and 5G), we investigated a potential role for non-canonical pathways in LC3 membrane recruitment during inflammation in venular ECs. In line with our *in vitro* findings, we found that >90% of GFP-LC3 puncta in IR-stimulated postcapillary venular ECs were WIPI2$^-$ (Figures 6A and 6B), further suggesting that non-canonical autophagy mechanisms could be operating in venular ECs *in vivo*. As ATG5-dependent non-canonical autophagy pathways, such as LC3-associated phagocytosis (LAP), involve the degradation of internalized plasma membrane and cell surface

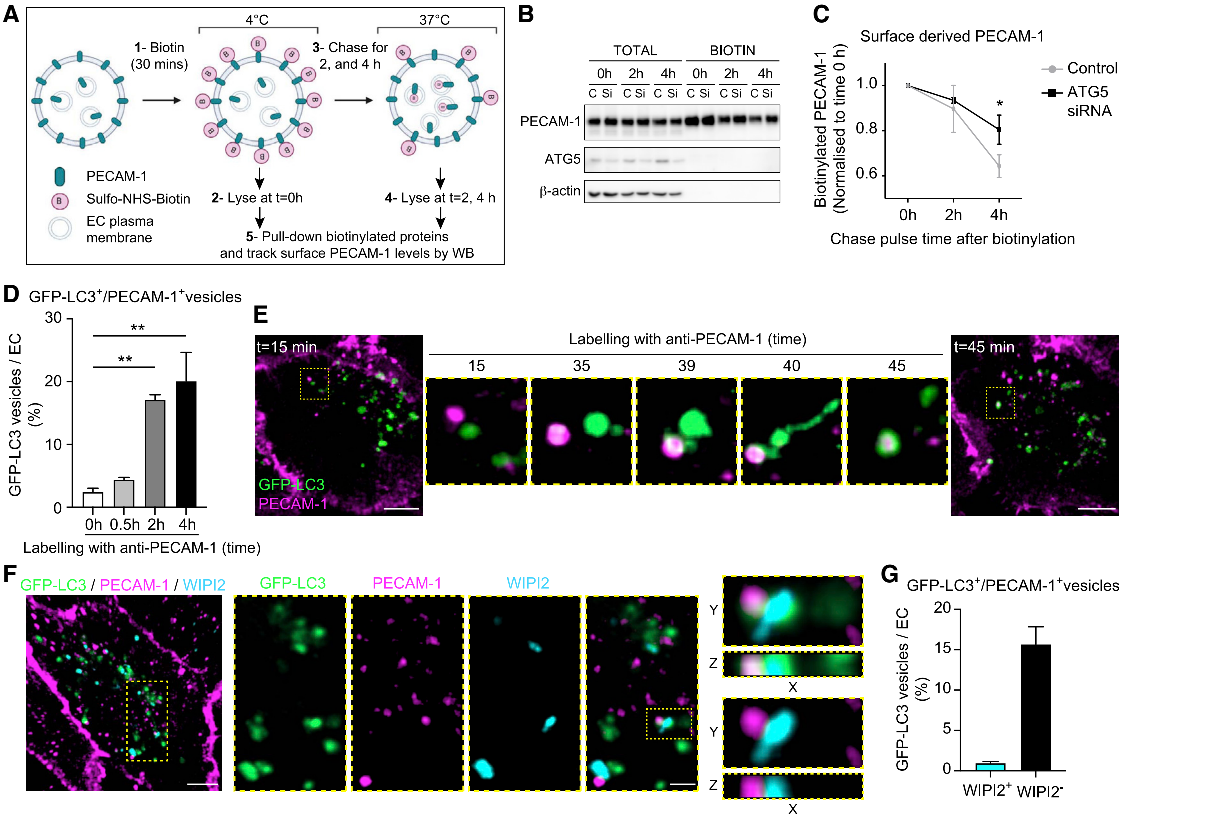

Figure 5. EC autophagy machinery regulates cell-surface PECAM-1 intracellular trafficking and degradation

(A–C) Schematic illustrating cell-surface biotinylation method. Control and ATG5 siRNA-silenced HUVECs were stimulated with LPS (A) and (B) immunoblotted for total and biotinylated PECAM-1, ATG5, and β-actin at the indicated times postbiotin incubation and (C) analyzed for fold change in cell surface-derived PECAM-1 (n = 7).

(D–G) GFP-LC3 transfected HUVECs were stimulated with LPS before antibody feeding using a nonblocking anti-PECAM-1 mAb.

(D) Number of GFP-LC3$^+$/ PECAM-1$^+$ vesicles at the indicated times after incubation with anti-PECAM-1 mAb (n = 3–5; 40–100 cells analyzed per condition).

(E) Time-lapse confocal images (Video S6) showing the formation of GFP-LC3$^+$/PECAM-1$^+$ vesicles (scale bars, 10 μm).

(F and G) Representative (n = 4) confocal images of GFP-LC3 transfected HUVECs immunostained for PECAM-1 and WIPI2 (scale bars, 10 μm and enlargements, 3 μm) (F) and (G) quantification of the number of GFP-LC3$^+$/ PECAM-1$^+$ vesicles WIPI2$^{-/+}$ (n = 4; >100 cells analyzed per condition).

Means ± SEMs. Statistically significant difference from controls is shown by *p < 0.05 and **p < 0.01.

See also Figure S5.

receptors (Florey et al., 2011; Martinez et al., 2016; Sanjuan et al., 2007), we explored the possibility that a similar pathway to LAP could be regulating the formation of LC3 puncta in ECs. Here, we used mice lacking the WD40 repeat-containing C-terminal domain of Atg16L1 (*Atg16L1$^{E230/E230}$*, hereafter referred to as *Atg16l1^{E230}*) that is essential for LC3 recruitment to endolysosomal membranes in LAP, but dispensable for canonical autophagy (Fletcher et al., 2018; Rai et al., 2019). Notably, IR-stimulated (4 h reperfusion) venular ECs of *Atg16L1^{E230}* mice exhibited significantly reduced numbers of LC3 puncta as compared to ECs of littermate control mice, as analyzed by IF (Figures 6C and 6D). Because this inhibition (~53%) was similar to that detected in *Atg5$^{-/-}$* ECs (~78%), as compared to WT ECs in *Atg5$^{\Delta EC}$* mice, the results identify the WD40 domain of ATG16L1 as a regulator of LC3 targeting to membranes in inflamed microvascular ECs *in vivo*. Functionally, as observed in *Atg5$^{\Delta EC}$* mice, IR-stimulated postcapillary venular ECs of *Atg16L1^{E230}* mice exhibited significant enrichment (>40%) of PECAM-1 at cell-cell contacts as compared to WT littermates (Figures 6E–6G) and showed a trend toward increased (>20%) VE-cadherin accumulation at junctions (Figures 6E, S6A, and S6B). These results identify a previously unknown role for a LAP-like non-canonical autophagy pathway in the regulation of EC junctional proteins in acute inflammation.

Genetic ablation of vascular autophagy leads to dysregulated neutrophil trafficking in multiple inflammatory models

To examine the broader functional consequences of ATG5-dependent modulation of EC junctions *in vivo*, we analyzed the impact of EC deficiency of ATG5 and the WD40 domain of ATG16L1 on leukocyte trafficking within multiple inflammatory models. Chimeric *Atg5$^{\Delta EC}$* mice exhibited enhanced neutrophil infiltration in an LPS-driven peritonitis model (Figure 7A) and in cutaneous models of inflammation as induced by locally injected LPS and interleukin-1β (IL-1β) (Figure 7B). Similarly, using the inducible mouse model, acute EC ATG5 deficiency promoted increased neutrophil infiltration in the LPS-driven peritonitis

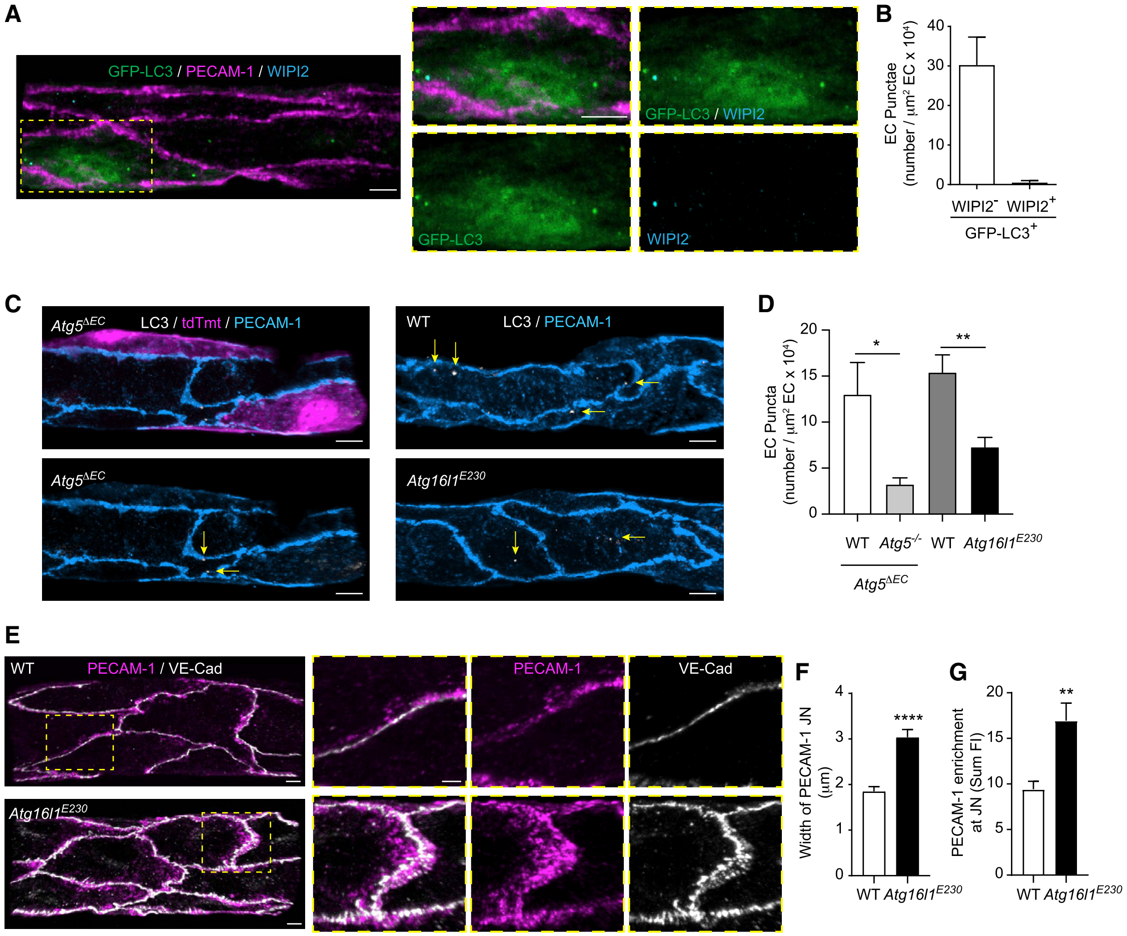

Figure 6. Non-canonical autophagy operates in microvascular ECs and regulates PECAM-1 distribution in response to IR injury
(A and B) *GFP-Map1lc3*$^{TG/+}$ mice were subjected to local IR injury.
(A and B) Representative (n = 3) confocal images of cremasteric PCVs (PECAM-1) immunostained for WIPI2, showing GFP-LC3^{+} and WIPI2^{+} puncta (scale bar, 5 µm) (A) and (B) number of GFP-LC3^{+}/WIPI2$^{-/+}$ puncta per venular EC area (n = 3 mice/group).
(C and D) *Atg5*$^{\Delta EC}$, WT, and *Atg16L1*^{E230} mice were subjected to local IR injury.
(C) Representative (n = 3–5) confocal images of cremasteric PCVs (PECAM-1) immunostained for endogenous LC3, with arrows indicating localization of LC3 puncta (scale bar, 5 µm).
(D) Number of LC3 puncta per venular EC area (n = 3–5 mice/group).
(E–G) WT and *Atg16L1*^{E230} mice were subjected to local IR injury.
(E) Representative (n = 3) confocal images of cremasteric PCVs (PECAM-1, VE-cadherin) (scale bars, 5 and 3 µm for enlargements) and associated quantification of PECAM-1.
(F and G) Junctional width (F) and (G) junctional enrichment (n = 3 mice/group).
Means ± SEMs. Statistically significant difference from controls is shown by *p < 0.05, **p < 0.01, and ****p < 0.0001.
See also Figure S6.

model (Figure 7C). In conjunction with the findings acquired from LPS- and IR-stimulated cremaster muscles (Figures 2A–2C), these results demonstrate that EC ATG5 can regulate neutrophil trafficking in multiple organs and in response to a range of stimuli. Moreover, ubiquitous *Atg16L1*^{E230} mice, or chimeric *Atg16L1*^{E230} mice exhibiting WT hematopoietic cells, showed increased neutrophil accumulation in a muramyl dipeptide (MDP)-induced peritonitis model and in response to IR in the cremaster muscle (Figures 7D–7F). Excessive neutrophil infiltration under conditions of EC ATG5 deficiency or EC ATG16L1 truncation suggested a non-redundant role for EC autophagy processes as principal modulators of acute inflammation. To investigate this further, we used a zymosan-induced peritonitis model of self-resolving acute inflammation (Damazo et al., 2006) to analyze the impact of EC ATG5 deficiency on the temporal profile of an inflammatory reaction *in vivo*. Locally injected zymosan elicited a rapid infiltration of neutrophils within the peritoneal cavity of chimeric *Atg5*$^{fl/fl}$ mice, a response that peaked by 28 h and declined toward basal after 40 h (Figure 7G). Chimeric *Atg5*$^{\Delta EC}$ mice showed a more rapid neutrophil recruitment, peaking at 16 h (Figure 7G), supporting the concept that EC autophagy restrains the breaching of venules and hence the onset of neutrophil infiltration in this model. The resolution phase of this reaction, which is known to be mediated by numerous responses

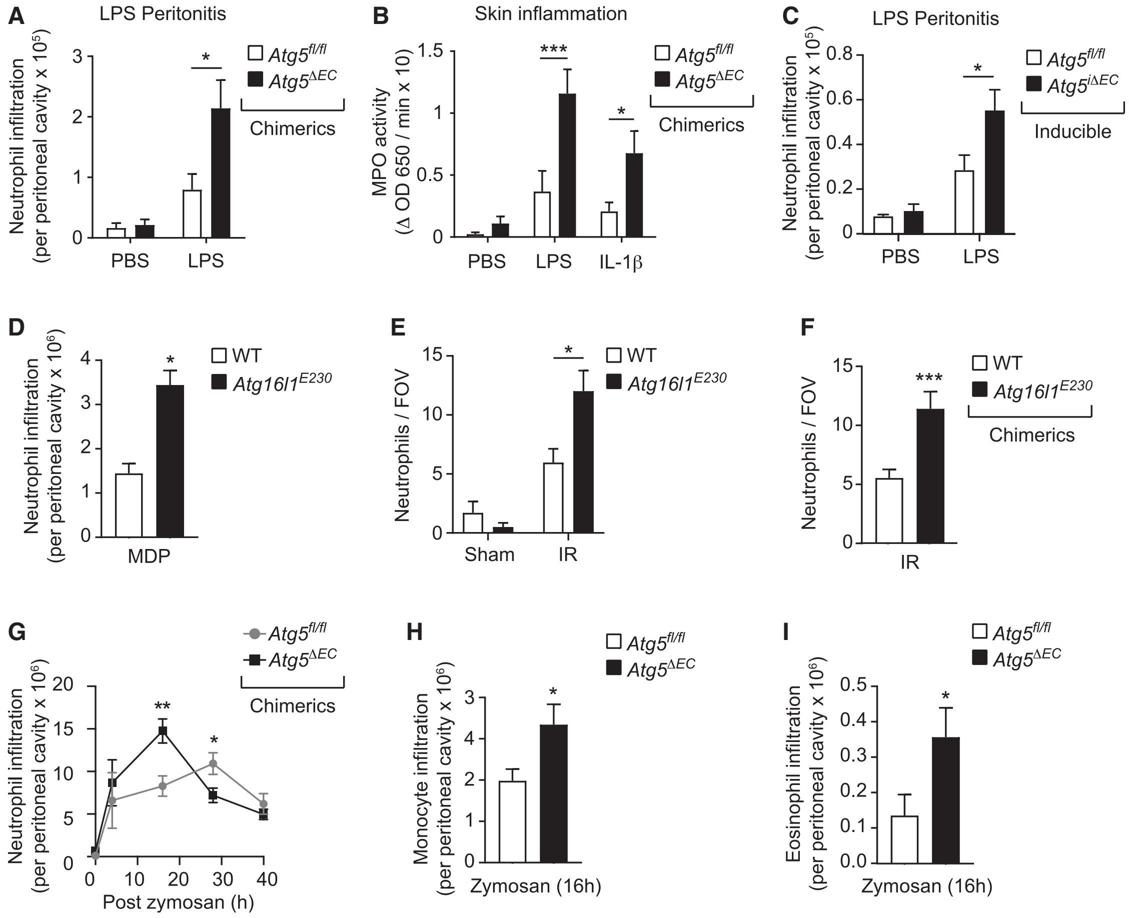

Figure 7. Genetic ablation of vascular autophagy (canonical and non-canonical) promotes exaggerated and more rapid leukocyte trafficking

(A and B) Chimeric *Atg5*$^{fl/fl}$ and *Atg5*$^{\Delta EC}$ mice were subjected to (A) LPS-induced peritonitis (n = 6–12 mice/group) or (B) skin inflammation (n = 5–6 mice/group), and neutrophil infiltration was quantified by flow cytometry or myeloperoxidase (MPO) activity, respectively.

(C) *Atg5*$^{fl/fl}$ and *Atg5*$^{i\Delta EC}$ mice were subjected to LPS-induced peritonitis, and neutrophil infiltration was quantified by flow cytometry (n = 4–9 mice/group).

(D) WT and *Atg16L1*^{E230} mice were subjected to MDP-induced peritonitis, and neutrophil infiltration was quantified by flow cytometry (n = 4–5 mice/group).

(E and F) Non-chimeric (E) and (F) chimeric WT and *Atg16L1*^{E230} mice were subjected to local IR injury, and neutrophil extravasation was assessed by confocal microscopy (n = 6–8 mice/group).

(G–I) Chimeric *Atg5*$^{fl/fl}$ and *Atg5*$^{\Delta EC}$ mice were subjected to zymosan-induced peritonitis, and infiltration of (G) neutrophils, (H) monocytes, and (I) eosinophils at the indicated times was quantified by flow cytometry (n = 3–9 mice/group).

Means ± SEMs. Statistically significant difference from controls or between indicated groups is shown by *$p < 0.05$, **$p < 0.01$, and ***$p < 0.001$.

at the inflammatory site, such as generation of pro-resolution mediators (Buckley et al., 2014; Perretti et al., 2017), was not impeded by EC autophagy (Figure 7G). Within this reaction, we also detected the enhanced recruitment of monocytes and eosinophils in chimeric *Atg5*$^{\Delta EC}$ mice (Figures 7H and 7I), demonstrating that the regulatory role of EC autophagy can be extrapolated to the trafficking of other leukocyte subtypes. Collectively, EC autophagy pathways played a pivotal role in modulating leukocyte trafficking in multiple acute inflammatory models *in vivo*.

DISCUSSION

Despite our growing understanding of the cellular and molecular events that support the onset and progression of neutrophil diapedesis, details of the mechanisms that terminate this response remain unclear. Here, we have identified EC autophagy as a molecular basis for the cessation of leukocyte recruitment in acute physiological inflammation. Specifically, we demonstrated that venular ECs upregulate autophagy in response to inflammation *in vivo*, and that genetic ablation of this reaction leads to increased tissue infiltration of neutrophils and tissue damage. Mechanistically, EC autophagy was necessary for regulating the expression of key EC adhesion molecules, most notably the trafficking and degradation of junctional molecules. These results present EC autophagy as a negative regulator of neutrophil TEM in acute inflammatory conditions.

The molecular basis of limiting inflammation is intricate and multifactorial. While inflammatory triggers can be disposed through enzymatic degradation, there is now substantial interest in the molecular basis and mode of action of endogenously generated pro-resolution pathways that can actively suppress

leukocyte recruitment (Perretti et al., 2017; Serhan and Levy, 2018). In addition, at the onset of an inflammatory reaction, leukocyte breaching of venular walls is controlled by altered expression and localization of key EC adhesion molecules (Nourshargh and Alon, 2014; Reglero-Real et al., 2016). As an example, aligned with efficient diapedesis, junctional molecules such as PECAM-1 and VE-cadherin exhibit intracellular recycling and degradation (Muller, 2016; Vestweber, 2015), although details of the associated molecular machineries require further exploration. Here, we explored the involvement of EC autophagy in neutrophil migration. The role of this degradative process as an essential regulator of immunity and inflammation is well established, but the concomitant mechanisms are complex and not fully defined (Deretic and Levine, 2018). Nonetheless, there is ample evidence for immune cell autophagy-related genes dampening inflammation, including diminishing immune cell trafficking. In contrast, while emerging *in vivo* evidence links autophagy-related genes to a wide array of EC functions, including shear stress-induced homeostasis, angiogenesis, and thrombosis (Sprott et al., 2019; Torisu et al., 2013; Vion et al., 2017; Verhoeven et al., 2021; Harlé et al., 2021), knowledge of EC autophagy as a regulator of inflammation is limited. Our findings demonstrated the occurrence and spatiotemporal regulation of autophagy (using GFP-LC3 puncta as readout) in acutely inflamed postcapillary venular ECs *in vivo*. We directly associated the induction of EC autophagy with the dynamics of neutrophil recruitment, suggesting a role for EC autophagy beyond the regulation of cell survival as previously described in inflammatory models of perturbed EC redox state (Schaaf et al., 2019). The localization of GFP-LC3 puncta at EC-EC contacts of inflamed tissues suggested a role for autophagic processes in neutrophil breaching of venular ECs. Simultaneous analysis of neutrophil interactions with WT or ATG5-deficient ECs within the same venular segment by confocal IVM revealed enhanced and faster neutrophil TEM across ATG5-compromised EC junctions. Autophagy-deficient EC contacts also acted as hotspot regions of exaggerated paracellular neutrophil TEM, and compromised EC autophagy supported increased neutrophil transcellular TEM. The overall impact of these cellular behaviors was increased infiltration of neutrophils into inflamed tissues, a finding recapitulated with several inflammatory stimuli, in a diverse range of tissues, and noted in the context of multiple leukocyte subtypes. Since excessive neutrophil extravasation in EC ATG5-deficient tissues was associated with enhanced tissue damage, and neutrophil trafficking was suppressed by local pharmacological induction of EC autophagy, the findings present activation of EC autophagy as a potential anti-inflammatory strategy.

Hypothesizing that autophagy may influence the expression of TEM-promoting molecules, we observed that EC ATG5 deficiency caused the accumulation of EC junctional adhesion molecules and the formation of thickened junctional regions. Ultrastructural analysis of inflamed venules by CLEM revealed PECAM-1$^+$ enlarged cell-cell contact sites and sheet-like plasma membrane flaps at ATG5-deficient EC junctions. Such aberrant structures could provide junctional sites with markedly accessible adhesion molecules, as indicated by the abrogation of neutrophil extravasation following pharmacological blockade of PECAM-1 in EC ATG5-deficient tissues. In addition to junctional proteins, increased cell-surface protein expression of a wide range of adhesion molecules on ATG5-deficient ECs suggested a broader role for EC autophagy in the regulation of leukocyte trafficking machinery. This did not appear to be transcriptionally regulated, but we detected EC surface PECAM-1, and potentially VE-cadherin, to traffic in LC3$^+$ vesicular compartments and be degraded in an ATG5-dependent manner in stimulated ECs. Suggesting defective junctional protein degradation and turnover in autophagy impaired ECs, these findings are in agreement with the targeting of junctional components by autophagy mechanisms reported in other cell types (Bejarano et al., 2012; Nighot et al., 2015; Wang et al., 2014). Regulated by a plethora of post-translational modifications, including phosphorylation and ubiquitination, vesicular trafficking of EC junctional components dictates their cellular localization and functional properties (Majolée et al., 2019; Wessel et al., 2014). Notably, both phosphorylation and ubiquitination act as molecular signals that can target specific pools of proteins to autophagic compartments (Bejarano et al., 2012; Levine and Kroemer, 2019). It is therefore plausible that EC autophagy impairment leads to an accumulation of modified junctional adhesion molecules with altered signaling properties that may recycle back to plasma membrane domains and influence the architecture of cell-cell contacts. Furthermore, as autophagy is regulated by molecular components of leukocyte diapedesis, such as connexins and caveolin-1 (Bejarano et al., 2014; Zhang et al., 2020), EC autophagy processes and junctional adhesion molecules may be reciprocally regulated.

EC molecules are known to distribute to a variety of junctionally connected compartments or vesicles (Reglero-Real et al., 2012; Fernandez-Martin et al., 2012). Such mechanisms contribute to maintaining the integrity of the endothelium or provide additional membranous pools that surround leukocytes as they pass across ECs. In addition to canonical autophagy, ATG5 can regulate other membrane-trafficking pathways, notably the non-canonical form of autophagy LAP that requires the WD40 domain of ATG16L1. Here, we discovered that a LAP-like pathway controlled EC LC3 puncta formation and PECAM-1 expression at EC borders of inflamed tissues, findings that report on a previously unidentified molecular trafficking mechanism in ECs. Initially described in macrophages and acting as an immunosuppressive mechanism, LAP plays an important role in the phagocytosis and clearance of pathogens and apoptotic or dead cells (Florey et al., 2011; Huang et al., 2009; Martinez et al., 2011). During this process, phagocytic vacuoles become decorated by lipidated LC3 for rapid targeting to and degradation by the lysosome. In contrast to canonical autophagy, LAP activation requires the engagement of plasma membrane receptors (e.g., Toll-like receptors [TLRs], immunoglobulin receptors) by extracellular cargo and the generation of reactive oxygen species (ROS), which in neutrophils and macrophages is mediated by the NADPH oxidase enzyme NOX2 (Heckmann et al., 2017). ECs similarly signal through TLR-4 when exposed to LPS (Tauseef et al., 2012) and generate NOX-dependent ROS following numerous inflammatory triggers (e.g., IR injury) (Frey et al., 2009). Since the latter phenomenon can regulate junctional destabilization (Nourshargh et al., 2010), the involvement of an EC LAP-like pathway in vascular junction dynamics in endotoxemia- and IR-driven acute inflammatory models is highly plausible.

CellPress
OPEN ACCESS

Finally, although speculative, the activation of a LAP-like pathway at EC contacts may be triggered by plasma membrane stretching and remodeling during neutrophil diapedesis. This response is analogous to the functional role of LAP in membrane repair following plasma and phagosomal membrane stress and damage induced by fungal pathogens (Westman et al., 2019). Our results suggest that a LAP-like non-canonical autophagy pathway in ECs orchestrates the degradation of internalized adhesion receptors present in vesicular compartments in a similar manner to targeting of phagosomal cargo-associated membranes in immune cells (Sanjuan et al., 2007). In addition, the WD40 domain of ATG16L1 may play a role in the internalization and signaling of EC adhesion molecules, as recently reported for cytokine receptors (Serramito-Gómez et al., 2020). Illustrated in mice deficient in the EC ATG16L1 WD40 domain, we identified the functional consequence of EC non-canonical autophagy deficiency as exaggerated neutrophil trafficking, a phenotype that recapitulated the results observed in EC ATG5-deficient animals. Importantly, these data suggest a role for derailed EC non-canonical autophagy signaling in the pathogenesis of inflammatory disorders linked to *Atg5* gene polymorphisms or to the deficiency of other core autophagy genes (Sachdev and Lotze, 2017). As such, it is crucial to revisit the well-established associations of autophagy with immune disorders, commonly aligned with canonical autophagy, to links with forms of non-canonical autophagy, such as LAP. Consistent with this notion, LAP deficiency in mice includes the establishment of lupus-like autoimmune disease (Martinez et al., 2016), a syndrome that has been linked to *Atg5* gene mutations in humans (Bentham et al., 2015).

In summary, this work identifies EC autophagy as a non-redundant negative modulator of leukocyte breaching of venular walls. Such a mechanism may prevent acute leukocyte-mediated tissue damage and hence suppress the potential onset of chronic inflammation. Mechanistically, we aligned this with the regulation of cell surface adhesion molecules and remodeling of EC contacts through the autophagic machinery. The involvement of a LAP-like non-canonical autophagy pathway in this context reveals a previously unidentified molecular trafficking mechanism operating in ECs. Our results suggest that the formation of aberrant EC junctions, with a high capacity for supporting leukocyte TEM, could act as a causal link between autophagy dysregulation and the development of numerous inflammatory conditions and aging-associated pathologies.

Limitations of study

While EC autophagy is identified as a negative modulator of neutrophil trafficking in multiple inflammatory models, the dynamics of neutrophil TEM were only analyzed in inflamed murine cremaster muscle microcirculation. Determining whether EC autophagy machinery regulates neutrophil TEM in other vascular beds, similarly operates in males and females, and contributes to the diapedesis of different leukocyte subtypes will be critical goals. Moreover, since following acute inflammation, EC autophagy deficiency elicits enhanced tissue damage, investigating the potential progression of this to chronic disease would be informative. Similarly, extending the findings to aging-associated pathologies that have been linked to autophagic deficiency will be an important avenue. Finally, having identified a functional role for non-canonical autophagy in ECs, the associated mechanisms require in-depth analysis. This includes defining the ultrastructure and nature of LC3^{+} puncta in inflamed ECs, as well as the molecular mechanisms targeting leukocyte trafficking machinery to this degradative pathway.

STAR★METHODS

Detailed methods are provided in the online version of this paper and include the following:

- KEY RESOURCES TABLE
- RESOURCE AVAILABILITY
  - Lead contact
  - Materials availability
  - Data and code availability
- EXPERIMENTAL MODEL AND SUBJECT DETAILS
  - Mice
  - Cell culture
- METHOD DETAILS
  - Generation of bone marrow chimeric mice
  - Inflammatory responses in the cremaster muscle
  - Cell transfection
  - Immunofluorescence staining
  - Confocal image acquisition of fixed samples
  - Confocal super-resolution imaging
  - Correlative light electron microscopy
  - Confocal intravital microscopy (IVM)
  - Time-lapse fluorescence imaging
  - *In vitro* neutrophil TEM assay
  - Peritoneal inflammation
  - Cutaneous inflammation
  - Mouse lung endothelial cell (MLEC) isolation
  - Flow cytometry and cell sorting
  - Real Time qRT-PCR analysis
  - Remote organ vascular leakage quantification
  - Cell surface biotinylation assays
  - Immunoblotting
- QUANTIFICATION AND STATISTICAL ANALYSIS
  - Quantification of confocal IVM videos
  - Image quantification of postcapillary venules in fixed cremaster muscle
  - Quantification of fixed HUVEC images
  - Statistics

SUPPLEMENTAL INFORMATION

Supplemental information can be found online at https://doi.org/10.1016/j.immuni.2021.07.012.

ACKNOWLEDGMENTS

This work was principally funded by the Wellcome Trust (098291/Z/12/Z, to S.N.). N.R.-R. is additionally supported by funding from the People Programme (Marie Curie Actions) of the European Union's Seventh Framework Programme (FP7/2007–2013) under REA grant agreement no. 608765. The work in the labs of S.A.T. and L.C. was supported by the Francis Crick Institute, which receives its core funding from Cancer Research UK (FC001187 and FC001999), the UK Medical Research Council (FC001187 and FC001999), and the Wellcome Trust (FC001187 and FC001999). E.A. was supported by the UK Medical Research Council and Barts charity grants MR/M023230/1 and MGU0488.

AUTHOR CONTRIBUTIONS

Conceptualization and writing (original draft), N.R.-R. and S.N.; methodology, investigation, analysis, and validation, S.N., N.R.-R., L.P.G., J.G.-M., C.P., A.B., A.Y., R.S.S., M.G.-N., L.R., S.N.A.-W., L.V.-M., W.A.M., T.F., G.D.R., M.G., M.-B.V., L.C., T.D.N., and E.A.; resources, S.N., C.M.B., Y.K., W.A.M., L.C., M.P., S.A.T., T.D.N., and E.A.; editing of draft, all authors; overall project conception, supervision, and funding, S.N.

DECLARATION OF INTERESTS

The authors declare no competing interests.

Received: August 19, 2020
Revised: May 13, 2021
Accepted: July 13, 2021
Published: August 6, 2021

REFERENCES

Alva, J.A., Zovein, A.C., Monvoisin, A., Murphy, T., Salazar, A., Harvey, N.L., Carmeliet, P., and Iruela-Arispe, M.L. (2006). VE-Cadherin-Cre-recombinase transgenic mouse: a tool for lineage analysis and gene deletion in endothelial cells. Dev. Dyn. *235*, 759–767.

Arganda-Carreras, I., Sorzano, C., Marabini, R., Carazo, J., Ortiz-de-Solorzano, C., and Kybic, J. (2006). Consistent and Elastic Registration of Histological Sections Using Vector-Spline Regularization. In Computer Vision Approaches to Medical Image Analysis, R.R. Beichel and M. Sonka, eds. (Springer).

Bejarano, E., Girao, H., Yuste, A., Patel, B., Marques, C., Spray, D.C., Pereira, P., and Cuervo, A.M. (2012). Autophagy modulates dynamics of connexins at the plasma membrane in a ubiquitin-dependent manner. Mol. Biol. Cell *23*, 2156–2169.

Bejarano, E., Yuste, A., Patel, B., Stout, R.F., Jr., Spray, D.C., and Cuervo, A.M. (2014). Connexins modulate autophagosome biogenesis. Nat. Cell Biol. *16*, 401–414.

Bentham, J., Morris, D.L., Graham, D.S.C., Pinder, C.L., Tombleson, P., Behrens, T.W., Martín, J., Fairfax, B.P., Knight, J.C., Chen, L., et al. (2015). Genetic association analyses implicate aberrant regulation of innate and adaptive immunity genes in the pathogenesis of systemic lupus erythematosus. Nat. Genet. *47*, 1457–1464.

Bhattacharya, A., Wei, Q., Shin, J.N., Abdel Fattah, E., Bonilla, D.L., Xiang, Q., and Eissa, N.T. (2015). Autophagy Is Required for Neutrophil-Mediated Inflammation. Cell Rep. *12*, 1731–1739.

Bogovic, J., Hanslovsky, P., Wong, A., and Saalfeld, S. (2016). Robust registration of calcium images by learned contrast synthesis. arXiv, 1511.01154v1.

Bolte, S., and Cordelières, F.P. (2006). A guided tour into subcellular colocalization analysis in light microscopy. J. Microsc. *224*, 213–232.

Buckley, C.D., Gilroy, D.W., and Serhan, C.N. (2014). Proresolving lipid mediators and mechanisms in the resolution of acute inflammation. Immunity *40*, 315–327.

Cardona, A., Saalfeld, S., Schindelin, J., Arganda-Carreras, I., Preibisch, S., Longair, M., Tomancak, P., Hartenstein, V., and Douglas, R.J. (2012). TrakEM2 software for neural circuit reconstruction. PLoS ONE *7*, e38011.

Damazo, A.S., Yona, S., Flower, R.J., Perretti, M., and Oliani, S.M. (2006). Spatial and temporal profiles for anti-inflammatory gene expression in leukocytes during a resolving model of peritonitis. J. Immunol. *176*, 4410–4418.

Deerinck, T.J., Bushong, E., Thor, A., and Ellisman, M. (2010). NCMIR methods for 3D EM: a new protocol for preparation of biological specimens for serial block face scanning electron microscopy. https://www.semanticscholar.org/paper/NCMIR-methods-for-3D-EM%3A-a-new-protocol-for-of-for-Deerinck-Bushong/49ebf43a6645985c3fb7d9e1336358b5b6d7cb8a.

Deerinck, T.J., Shone, T.M., Bushong, E.A., Ramachandra, R., Peltier, S.T., and Ellisman, M.H. (2018). High-performance serial block-face SEM of nonconductive biological samples enabled by focal gas injection-based charge compensation. J. Microsc. *270*, 142–149.

Dejana, E., Tournier-Lasserve, E., and Weinstein, B.M. (2009). The control of vascular integrity by endothelial cell junctions: molecular basis and pathological implications. Dev. Cell *16*, 209–221.

Deretic, V., and Levine, B. (2018). Autophagy balances inflammation in innate immunity. Autophagy *14*, 243–251.

Deretic, V. (2021). Autophagy in inflammation, infection, and immunometabolism. Immunity *54*, 437–453.

Dooley, H.C., Razi, M., Polson, H.E., Girardin, S.E., Wilson, M.I., and Tooze, S.A. (2014). WIPI2 links LC3 conjugation with PI3P, autophagosome formation, and pathogen clearance by recruiting Atg12-5-16L1. Mol. Cell *55*, 238–252.

Engelhardt, B., and Ransohoff, R.M. (2012). Capture, crawl, cross: the T cell code to breach the blood-brain barriers. Trends Immunol. *33*, 579–589.

Faust, N., Varas, F., Kelly, L.M., Heck, S., and Graf, T. (2000). Insertion of enhanced green fluorescent protein into the lysozyme gene creates mice with green fluorescent granulocytes and macrophages. Blood *96*, 719–726.

Feng, D., Nagy, J.A., Pyne, K., Dvorak, H.F., and Dvorak, A.M. (2004). Ultrastructural localization of platelet endothelial cell adhesion molecule (PECAM-1, CD31) in vascular endothelium. J. Histochem. Cytochem. *52*, 87–101.

Fernandez-Martin, L., Marcos-Ramiro, B., Bigarella, C.L., Graupera, M., Cain, R.J., Reglero-Real, N., Jimenez, A., Cernuda-Morollon, E., Correas, I., Cox, S., et al. (2012). Crosstalk between reticular adherens junctions and platelet endothelial cell adhesion molecule-1 regulates endothelial barrier function. Arterioscler Thromb Vasc Biol *32*, e90–e102.

Fletcher, K., Ulferts, R., Jacquin, E., Veith, T., Gammoh, N., Arasteh, J.M., Mayer, U., Carding, S.R., Wileman, T., Beale, R., and Florey, O. (2018). The WD40 domain of ATG16L1 is required for its non-canonical role in lipidation of LC3 at single membranes. EMBO J. *37*, e97840.

Florey, O., Kim, S.E., Sandoval, C.P., Haynes, C.M., and Overholtzer, M. (2011). Autophagy machinery mediates macroendocytic processing and entotic cell death by targeting single membranes. Nat. Cell Biol. *13*, 1335–1343.

Frey, R.S., Ushio-Fukai, M., and Malik, A.B. (2009). NADPH oxidase-dependent signaling in endothelial cells: role in physiology and pathophysiology. Antioxid. Redox Signal. *11*, 791–810.

Girbl, T., Lenn, T., Perez, L., Rolas, L., Barkaway, A., Thiriot, A., Del Fresno, C., Lynam, E., Hub, E., Thelen, M., et al. (2018). Distinct Compartmentalization of the Chemokines CXCL1 and CXCL2 and the Atypical Receptor ACKR1 Determine Discrete Stages of Neutrophil Diapedesis. Immunity *49*, 1062–1076.e6.

Hara, T., Nakamura, K., Matsui, M., Yamamoto, A., Nakahara, Y., Suzuki-Migishima, R., Yokoyama, M., Mishima, K., Saito, I., Okano, H., and Mizushima, N. (2006). Suppression of basal autophagy in neural cells causes neurodegenerative disease in mice. Nature *441*, 885–889.

Harlé, G., Kowalski, C., Dubrot, J., Brighouse, D., Clavel, G., Pick, R., Bessis, N., Niven, J., Scheiermann, C., Gannagé, M., and Hugues, S. (2021). Macroautophagy in lymphatic endothelial cells inhibits T cell-mediated autoimmunity. J. Exp. Med. *218*, e20201776.

Heckmann, B.L., Boada-Romero, E., Cunha, L.D., Magne, J., and Green, D.R. (2017). LC3-Associated Phagocytosis and Inflammation. J. Mol. Biol. *429*, 3561–3576.

Hobbs, J.A., May, R., Tanousis, K., McNeill, E., Mathies, M., Gebhardt, C., Henderson, R., Robinson, M.J., and Hogg, N. (2003). Myeloid cell function in MRP-14 (S100A9) null mice. Mol. Cell. Biol. *23*, 2564–2576.

Huang, J., Canadien, V., Lam, G.Y., Steinberg, B.E., Dinauer, M.C., Magalhaes, M.A., Glogauer, M., Grinstein, S., and Brumell, J.H. (2009). Activation of antibacterial autophagy by NADPH oxidases. Proc. Natl. Acad. Sci. USA *106*, 6226–6231.

Jiang, L., Lin, L., Li, R., Yuan, C., Xu, M., Huang, J.H., and Huang, M. (2016). Dimer conformation of soluble PECAM-1, an endothelial marker. Int. J. Biochem. Cell Biol. *77* (Pt A), 102–108.

Kabeya, Y., Mizushima, N., Ueno, T., Yamamoto, A., Kirisako, T., Noda, T., Kominami, E., Ohsumi, Y., and Yoshimori, T. (2000). LC3, a mammalian homologue of yeast Apg8p, is localized in autophagosome membranes after processing. EMBO J. *19*, 5720–5728.

CellPress
OPEN ACCESS

Klionsky, D.J., Abdelmohsen, K., Abe, A., Abedin, M.J., Abeliovich, H., Acevedo Arozena, A., Adachi, H., Adams, C.M., Adams, P.D., Adeli, K., et al. (2016). Guidelines for the use and interpretation of assays for monitoring autophagy (3rd edition). Autophagy *12*, 1–222.

Kremer, J.R., Mastronarde, D.N., and McIntosh, J.R. (1996). Computer visualization of three-dimensional image data using IMOD. J. Struct. Biol. *116*, 71–76.

Ktistakis, N.T., and Tooze, S.A. (2016). Digesting the Expanding Mechanisms of Autophagy. Trends Cell Biol. *26*, 624–635.

Lamagna, G., Meda, P., Mandicourt, G., Brown, J., Gilbert, R.J., Jones, E.Y., Kiefer, F., Ruga, P., Imhof, B.A., and Aurrad-Lions, M. (2005). Dual interaction of JAM-C with JAM-B and alpha(M)beta2 integrin: function in junctional complexes and leukocyte adhesion. Mol Biol Cell *16*, 4992–5003.

Levine, B., and Kroemer, G. (2019). Biological Functions of Autophagy Genes: A Disease Perspective. Cell *176*, 11–42.

Ley, K., Laudanna, C., Cybulsky, M.I., and Nourshargh, S. (2007). Getting to the site of inflammation: the leukocyte adhesion cascade updated. Nat. Rev. Immunol. *7*, 678–689.

Majolée, J., Kovačević, I., and Hordijk, P.L. (2019). Ubiquitin-based modifications in endothelial cell-cell contact and inflammation. J. Cell Sci. *132*, jcs227728.

Mamdouh, Z., Chen, X., Pierini, L.M., Maxfield, F.R., and Muller, W.A. (2003). Targeted recycling of PECAM from endothelial surface-connected compartments during diapedesis. Nature *421*, 748–753.

Martinez, J., Almendinger, J., Oberst, A., Ness, R., Dillon, C.P., Fitzgerald, P., Hengartner, M.O., and Green, D.R. (2011). Microtubule-associated protein 1 light chain 3 alpha (LC3)-associated phagocytosis is required for the efficient clearance of dead cells. Proc. Natl. Acad. Sci. USA *108*, 17396–17401.

Martinez, J., Cunha, L.D., Park, S., Yang, M., Lu, Q., Orchard, R., Li, Q.Z., Yan, M., Janke, L., Guy, C., et al. (2016). Noncanonical autophagy inhibits the autoinflammatory, lupus-like response to dying cells. Nature *533*, 115–119.

Martinez-Martin, N., Maldonado, P., Gasparrini, F., Frederico, B., Aggarwal, S., Gaya, M., Tsui, C., Burbage, M., Keppler, S.J., Montaner, B., et al. (2017). A switch from canonical to noncanonical autophagy shapes B cell responses. Science *355*, 641–647.

Mizushima, N., Yamamoto, A., Matsui, M., Yoshimori, T., and Ohsumi, Y. (2004). In vivo analysis of autophagy in response to nutrient starvation using transgenic mice expressing a fluorescent autophagosome marker. Mol. Biol. Cell *15*, 1101–1111.

Muller, W.A. (2011). Mechanisms of leukocyte transendothelial migration. Annu. Rev. Pathol. *6*, 323–344.

Muller, W.A. (2016). Localized signals that regulate transendothelial migration. Curr. Opin. Immunol. *38*, 24–29.

Muller, W.A., and Luscinskas, F.W. (2008). Assays of transendothelial migration in vitro. Methods Enzymol. *443*, 155–176.

Muller, W.A., Weigl, S.A., Deng, X., and Phillips, D.M. (1993). PECAM-1 is required for transendothelial migration of leukocytes. J. Exp. Med. *178*, 449–460.

Nighot, P.K., Hu, C.A., and Ma, T.Y. (2015). Autophagy enhances intestinal epithelial tight junction barrier function by targeting claudin-2 protein degradation. J. Biol. Chem. *290*, 7234–7246.

Nourshargh, S., and Alon, R. (2014). Leukocyte migration into inflamed tissues. Immunity *41*, 694–707.

Nourshargh, S., Hordijk, P.L., and Sixt, M. (2010). Breaching multiple barriers: leukocyte motility through venular walls and the interstitium. Nat. Rev. Mol. Cell Biol. *11*, 366–378.

Nussenzweig, S.C., Verma, S., and Finkel, T. (2015). The role of autophagy in vascular biology. Circ. Res. *116*, 480–488.

Okabe, K., Kobayashi, S., Yamada, T., Kurihara, T., Tai-Nagara, I., Miyamoto, T., Mukouyama, Y.S., Sato, T.N., Suda, T., Ema, M., and Kubota, Y. (2014). Neurons limit angiogenesis by titrating VEGF in retina. Cell *159*, 584–596.

Peraro, L., Zou, Z., Makwana, K.M., Cummings, A.E., Ball, H.L., Yu, H., Lin, Y.S., Levine, B., and Kritzer, J.A. (2017). Diversity-Oriented Stapling Yields Intrinsically Cell-Penetrant Inducers of Autophagy. J. Am. Chem. Soc. *139*, 7792–7802.

Perretti, M., Cooper, D., Dalli, J., and Norling, L.V. (2017). Immune resolution mechanisms in inflammatory arthritis. Nat. Rev. Rheumatol. *13*, 87–99.

Pham, D.L., Kim, S.H., Losol, P., Yang, E.M., Shin, Y.S., Ye, Y.M., and Park, H.S. (2016). Association of autophagy related gene polymorphisms with neutrophilic airway inflammation in adult asthma. Korean J. Intern. Med. (Korean. Assoc. Intern. Med.) *31*, 375–385.

Rai, S., Arasteh, M., Jefferson, M., Pearson, T., Wang, Y., Zhang, W., Bicsak, B., Divekar, D., Powell, P.P., Naumann, R., et al. (2019). The ATG5-binding and coiled coil domains of ATG16L1 maintain autophagy and tissue homeostasis in mice independently of the WD domain required for LC3-associated phagocytosis. Autophagy *15*, 599–612.

Reglero-Real, N., Colom, B., Bodkin, J.V., and Nourshargh, S. (2016). Endothelial Cell Junctional Adhesion Molecules: Role and Regulation of Expression in Inflammation. Arterioscler. Thromb. Vasc. Biol. *36*, 2048–2057.

Reglero-Real, N., Marcos-Ramiro, B., and Millán, J. (2012). Endothelial membrane reorganization during leukocyte extravasation. Cell. Mol. Life Sci. *69*, 3079–3099.

Riffelmacher, T., Clarke, A., Richter, F.C., Stranks, A., Pandey, S., Danielli, S., Hublitz, P., Yu, Z., Johnson, E., Schwerd, T., et al. (2017). Autophagy-Dependent Generation of Free Fatty Acids Is Critical for Normal Neutrophil Differentiation. Immunity *47*, 466–480.e5.

Sachdev, U., and Lotze, M.T. (2017). Perpetual change: autophagy, the endothelium, and response to vascular injury. J. Leukoc. Biol. *102*, 221–235.

Sanjuan, M.A., Dillon, C.P., Tait, S.W., Moshiach, S., Dorsey, F., Connell, S., Komatsu, M., Tanaka, K., Cleveland, J.L., Withoff, S., and Green, D.R. (2007). Toll-like receptor signalling in macrophages links the autophagy pathway to phagocytosis. Nature *450*, 1253–1257.

Santovito, D., Egea, V., Bidzhekov, K., Natarelli, L., Mourão, A., Blanchet, X., Wichapong, K., Aslani, M., Brunßen, C., Horckmans, M., et al. (2020). Noncanonical inhibition of caspase-3 by a nuclear microRNA confers endothelial protection by autophagy in atherosclerosis. Sci. Transl. Med. *12*, eaaz2294.

Serramito-Gómez, I., Boada-Romero, E., Villamuera, R., Fernández-Cabrera, Á., Cedillo, J.L., Martín-Regalado, Á., Carding, S., Mayer, U., Powell, P.P., Wileman, T., et al. (2020). Regulation of cytokine signaling through direct interaction between cytokine receptors and the ATG16L1 WD40 domain. Nat. Commun. *11*, 5919.

Schaaf, M.B., Houbaert, D., Meçe, O., and Agostinis, P. (2019). Autophagy in endothelial cells and tumor angiogenesis. Cell Death Differ. *26*, 665–679.

Schindelin, J., Arganda-Carreras, I., Frise, E., Kaynig, V., Longair, M., Pietzsch, T., Preibisch, S., Rueden, C., Saalfeld, S., Schmid, B., et al. (2012). Fiji: an open-source platform for biological-image analysis. Nat. Methods *9*, 676–682.

Serhan, C.N., and Levy, B.D. (2018). Resolvins in inflammation: emergence of the pro-resolving superfamily of mediators. J. Clin. Invest. *128*, 2657–2669.

Sprott, D., Poitz, D.M., Korovina, I., Ziogas, A., Phieler, J., Chatzigeorgiou, A., Mitroulis, I., Deussen, A., Chavakis, T., and Klotzsche-von Ameln, A. (2019). Endothelial-Specific Deficiency of ATG5 (Autophagy Protein 5) Attenuates Ischemia-Related Angiogenesis. Arterioscler. Thromb. Vasc. Biol. *39*, 1137–1148.

Tauseef, M., Knezevic, N., Chava, K.R., Smith, M., Sukriti, S., Gianaris, N., Obukhov, A.G., Vogel, S.M., Schraufnagel, D.E., Dietrich, A., et al. (2012). TLR4 activation of TRPC6-dependent calcium signaling mediates endotoxin-induced lung vascular permeability and inflammation. J. Exp. Med. *209*, 1953–1968.

Torisu, T., Torisu, K., Lee, I.H., Liu, J., Malide, D., Combs, C.A., Wu, X.S., Rovira, I.I., Fergusson, M.M., Weigert, R., et al. (2013). Autophagy regulates endothelial cell processing, maturation and secretion of von Willebrand factor. Nat. Med. *19*, 1281–1287.

Verhoeven, J., Baelen, J., Agrawal, M., and Agostinis, P. (2021). Endothelial cell autophagy in homeostasis and cancer. FEBS Lett. *595*, 1497–1511.

Vestweber, D. (2015). How leukocytes cross the vascular endothelium. Nat. Rev. Immunol. *15*, 692–704.

Vion, A.-C., Kheloufi, M., Hammoutene, A., Poisson, J., Lasselin, J., Devue, C., Pic, I., Dupont, N., Busse, J., Stark, K., et al. (2017). Autophagy is required for endothelial cell alignment and atheroprotection under physiological blood flow. Proc. Natl. Acad. Sci. USA *114*, E8675–E8684.

Wang, B.S., Yang, Y., Lu, H.Z., Shang, L., Zhang, Y., Hao, J.J., Shi, Z.Z., Wang, X.M., Liu, Y.Z., Zhan, Q.M., et al. (2014). Inhibition of atypical protein kinase Cι induces apoptosis through autophagic degradation of β-catenin in esophageal cancer cells. Mol. Carcinog. *53*, 514–525.

Wessel, F., Winderlich, M., Holm, M., Frye, M., Rivera-Galdos, R., Vockel, M., Linnepe, R., Ipe, U., Stadtmann, A., Zarbock, A., et al. (2014). Leukocyte extravasation and vascular permeability are each controlled in vivo by different tyrosine residues of VE-cadherin. Nat. Immunol. *15*, 223–230.

Westman, J., Hube, B., and Fairn, G.D. (2019). Integrity under stress: host membrane remodelling and damage by fungal pathogens. Cell. Microbiol. *21*, e13016.

Woodfin, A., Voisin, M.B., Beyrau, M., Colom, B., Caille, D., Diapouli, F.M., Nash, G.B., Chavakis, T., Albelda, S.M., Rainger, G.E., et al. (2011). The junctional adhesion molecule JAM-C regulates polarized transendothelial migration of neutrophils in vivo. Nat. Immunol. *12*, 761–769.

Yu, L., Chen, Y., and Tooze, S.A. (2018). Autophagy pathway: cellular and molecular mechanisms. Autophagy *14*, 207–215.

Zhang, X., Ramírez, C.M., Aryal, B., Madrigal-Matute, J., Liu, X., Diaz, A., Torrecilla-Parra, M., Suárez, Y., Cuervo, A.M., Sessa, W.C., and Fernández-Hernando, C. (2020). Cav-1 (Caveolin-1) Deficiency Increases Autophagy in the Endothelium and Attenuates Vascular Inflammation and Atherosclerosis. Arterioscler. Thromb. Vasc. Biol. *40*, 1510–1522.

CellPress
OPEN ACCESS

STAR★METHODS

KEY RESOURCES TABLE

REAGENT or RESOURCE	SOURCE	IDENTIFIER
Antibodies		
Anti-mouse PECAM-1 (clone 390)	Thermo Fisher Scientific	Cat#16-0311-85; RRID: AB_468933
Blocking anti-mouse PECAM-1 (clone Mec12.3)	Biolegend	Cat#102502; RRID: AB_312909
Anti-mouse IgG2a, κ isotype control (clone RTK2758)	Biolegend	Cat#400502; RRID: AB_2736919
Anti-mouse CD102 (clone 3C4)	Biolegend	Cat#105602; RRID: AB_313195
Anti-mouse CD45 (clone 30-F11)	Biolegend	Cat#103102; RRID: AB_312967
Anti-mouse ICAM-1 (clone YN1)	Biolegend	Cat#116102; RRID: AB_313693
Anti-mouse Ly6G (clone 18A)	Biolegend	Cat#127602; RRID: AB_1089180
Anti-mouse MRP14 (clone 2B10)	Gift from Dr N. Hogg (The Francis Crick Institute, UK) (Hobbs et al., 2003)	N/A
Anti-mouse CD144 (clone BV14)	Thermo Fisher Scientific	Cat#14-1442-82; RRID: AB_891369
Polyclonal anti-mouse GFP	Abcam	Cat#ab6662; RRID: AB_305635
Anti-mouse JAM-C (clone 114.2)	Gift from Dr M. Aurrand-Lions (INSERM, France) (Lamagna et al., 2005)	N/A
Polyclonal anti-mouse LC3	Thermo Fisher Scientific	Cat#PA1-16930; RRID: AB_2281384
Anti-mouse WIPI2	Gift from Dr S. Tooze (The Francis Crick Institute, UK) (Dooley et al., 2014)	N/A
Anti-mouse F4/80 (clone BM8)	Biolegend	Cat#123102; RRID: AB_893506
Anti-mouse Ly6C (clone HK1.4)	Biolegend	Cat#128002; RRID: AB_1134214
Anti-mouse SiglecF (clone S17007L)	Biolegend	Cat#155502; RRID: AB_2810420
Anti-mouse CD11b (clone M1/70)	Biolegend	Cat#1012020; RRID: AB_312785
Anti-mouse neutrophil (clone 7/4)	Abcam	Cat#ab53453; RRID: AB_881408
Anti-mouse CD16/CD32 (CLONE 2.4G2)	BDBiosciences	Cat#553141; RRID: AB_394656
Anti-human PECAM-1 (clone WM59)	Thermo Fisher Scientific	Cat#14-0319-82; RRID: AB_467204
Blocking anti-human PECAM-1	Generated in the lab of Dr W. A. Muller (Feinberg School of Medicine, Chicago, IL, USA) (Mamdouh et al., 2003)	N/A

(*Continued on next page*)

Continued

REAGENT or RESOURCE	SOURCE	IDENTIFIER
Polyclonal anti-human LC3	Cell Signaling Technology	Cat#2775; RRID: AB_915950
Anti-human Atg5 (clone D5F5U)	Cell Signaling Technology	Cat#12994; RRID: AB_2630393
Anti-human β-Actin (clone AC-15)	Sigma-Aldrich	Cat#A5441; RRID: AB_476744
Anti-human VE-Cadherin (F-8)	Santa Cruz Biotechnology	Cat#sc-9989 RRID: AB_2077957
**Chemicals, peptides, and recombinant proteins**		
LPS	Sigma-Aldrich	Cat#A9543
Peptidoglican G from Staphylococcus aureus	Sigma-Aldrich	Cat#77410-10mg
Zymosan A	Sigma-Aldrich	Cat#Z4250
Muramydipeptide (MDP, L-D isomer, active)	InvivoGen	Cat#tlrl-mdp
IL1β	R&D	Cat#201-LB-010
M199 medium	GIBCO-BRL	Cat#11150059
DMEM	GIBCO-BRL	Cat#21068028
Fetal Bovine Serum (FBS)	Sigma-Aldrich	Cat#F9665
Heparin	Sigma-Aldrich	Cat#H3393
Endothelial Cell Growth Supplement (ECGS)	Sigma-Aldrich	Cat#E2759
Fetal Calf Serum (FCS)	Thermo Fisher Scientific	Cat# 26010074
Paraformaldehyde (PFA)	Sigma-Aldrich	Cat#158127
EZ-Link Sulfo-NHS-Biotin	Thermo Fisher Scientific	Cat#21217
Pierce NeutrAvidin Agarose	Thermo Fisher Scientific	Cat#29201
Triton X-100	Sigma-Aldrich	Cat#9002-93-1
Bovine Serum Albumin (BSA)	Sigma-Aldrich	Cat#A9543
Tyrode’s solution	Sigma-Aldrich	Cat#T1788
Bafilomycin A1	AlfaAesar (Avocado Research Chemicals)	Cat# J67193
Pig Skin Gelatin	Sigma-Aldrich	Cat#9000-70-8
EDTA	Sigma-Aldrich	Cat#EDS
Halt Protease and phosphatase inhibitors	Thermo Fisher Scientific	Cat#78440
3,3′,5,5′-tetramethylbenzidine	Invitrogen	Cat#N301
2X Laemmli Sample Buffer	Bio-Rad	Cat#1610737
Type I Collagenase	GIBCO	Cat#10114532
Tat-Beclin 1 D11 Autophagy Inducing Peptide - Retroinverso form	Bio-techne	Cat#NBP2-49888
Tat-Beclin 1 L11S Peptide - Scrambled Control	Bio-techne	Cat#NBP2-49887
Propidium Iodide	Sigma-Aldrich	Cat#P4864
**Critical commercial assays**		
Alexa Fluor 488 Antibody Labeling Kit	Thermo Fisher Scientific	Cat#A20181
Alexa Fluor 555 Antibody Labeling Kit	Thermo Fisher Scientific	Cat#A20187
Alexa Fluor 647 Antibody Labeling Kit	Thermo Fisher Scientific	Cat#A20186
DyLight 405 antibody Labeling Kit	Thermo Fisher Scientific	Cat#53021
Yellow Zombie Fixable Viability Kit	Biolegend	Cat#423103
RNeasy Micro Kit	QIAGEN	Cat#74004
iScript cDNA Synthesis Kit	Bio-Rad	Cat#4106228
iQ SYBR Green Supermix	Bio-Rad	Cat#1708880

(*Continued on next page*)

Continued

REAGENT or RESOURCE	SOURCE	IDENTIFIER
Experimental models: Cell lines		
Human umbilical vein endothelial cells (HUVECs)	PromoCell	Cat#C-14008
Experimental models: Organisms/strains		
Mouse, *GFP-LC3*$^{TG/+}$	Generated in the lab of Prof. N. Mizushima (RIKEN BRC through the National Bio-Resource Project of the MEXT, Japan) (Mizushima et al., 2004)	N/A
Mouse, *Cdh5-Cre; Atg5*$^{fl/fl}$	Generated in the lab of Dr C.Boulanger (INSERM, France) (Vion et al., 2017)	N/A
Mouse, *Rosa26*$^{CAG\text{-}loxP\text{-}STOP\text{-}loxP\text{-}tdTomato/\ CAG\text{-}loxP\text{-}STOP\text{-}loxP\text{-}tdTomato}$	The Jackson Laboratory	JAX 007905
Mouse,*Cdh5-CreER*^{T2} (*Cdh5(BAC)-CreER*^{T2}*; Rosa26*$^{tdTomato/tdTomato}$)	Generated in the lab of Prof. Y. Kubota (Keio University School of Medicine, Japan) (Okabe et al., 2014)	N/A
Mouse, *Lyz2-EGFP-ki*	Gift from Dr M. Sperandio (Ludwig Maximilians University of Munich, Germany) (Faust et al., 2000)	N/A
Mouse, *Atg16l1*$^{E230/E230}$	Generated in the lab of Prof T. Wileman (Quadram Institute, UK) (Rai et al., 2019)	N/A
Oligonucleotides		
Atg5 ON-TARGETplus siRNA SMARTpooL 5′-GGCAUU AUCCAAUUGGUUU-3′ 5′-GCAGAACCAUACUAUUU GC-3′, 5′-UGACAGAUUUGACCAGUUU-3′ 5′-ACAAA GAUGUGCUUCGAGA-3′	Dharmacon Inc; Horizon Discovery	Cat# L-004374-00-0005
Luciferase ON-TARGETplus siRNA 5′-CGUACGC GGAAUACUUCGA-3′	Dharmacon Inc; Horizon Discovery	Cat#D-002050-01-20
Real-time PCR primer: *Gapdh* Forward 5′- TCGTGG ATCTGACGTGCCGCCTG-3′	This paper	N/A
Real-time PCR primer: *Gapdh* Reverse 5′- CACCAC CCTGTTGCTGTAGCCGTA-3′	This paper	N/A
Real-time PCR primer: *Pecam-1* Forward 5′- AGGTGG AACAAGCACAGATG-3′	This paper	N/A
Real-time PCR primer: *Pecam-1* Reverse 5′- TTGCTG ATGGCACCATCTTC-3′	This paper	N/A
Real-time PCR primer: *Cdh5* Forward 5′-CAGCCC AAAGTGTGTGAGAA-3′	This paper	N/A
Real-time PCR primer: *Cdh5* Reverse 5′- GAACTTC ACGTTTCGTGGTG-3′	This paper	N/A
Real-time PCR primer: *Atg5* Forward 5′- GCAGTAG GCTTGAGTGAACT-3′	This paper	N/A
Real-time PCR primer: *Atg5* Reverse 5′- CTAGGG CATTGTAGGCTTGA-3′	This paper	N/A
Real-time PCR primer: *TdTomato* Forward 5′- CAAGC TGGACCATCACCTCC-3′	This paper	N/A
Real-time PCR primer: *TdTomato* Reverse 5′- TGCCC GTACAGGAACAGGTG-3′	This paper	N/A
Real-time PCR primer: *E-selectin* Forward 5′- AGCTA CCCATGGAACACGAC −3′	This paper	N/A

(*Continued on next page*)

Continued

REAGENT or RESOURCE	SOURCE	IDENTIFIER
Real-time PCR primer: *E-selectin* Reverse 5′- ACGCAA GTTCTCCAGCTGTT-3′	This paper	N/A
Real-time PCR primer: *Icam-1* Forward 5′- GTGCAA TCATGGTTCAGTGC –3′	This paper	N/A
Real-time PCR primer: *Icam-1* Reverse 5′- GTGTGG TGTTGTGAGCCTAT –3′	This paper	N/A
Real-time PCR primer: *Icam-2* Forward 5′- ATCAAC TGCAGCACCAACTG –3′	This paper	N/A
Real-time PCR primer: *Icam-2* Reverse 5′- ACTTGA GCTGGAGGCTGGTA-3′	This paper	N/A
Real-time PCR primer: *Vcam-1* Forward 5′- TCTTGG GAGCCTCAACGGTA –3′	This paper	N/A
Real-time PCR primer: *Vcam-1* Reverse 5′- CAAGTG AGGGCCATGGAGTC –3′	This paper	N/A
Real-time PCR primer: *Cxcl1* Forward 5′- CCGAAG TCATAGCCACACTCAA –3′	This paper	N/A
Real-time PCR primer: *Cxcl1* Reverse 5′- GCAGTC TGTCTTCTTTCTCCGTTA –3′	This paper	N/A
Real-time PCR primer: *TnfRI* Forward 5′- CAGTCT GCAGGGAGTGTGAA-3′	This paper	N/A
Real-time PCR primer: *TnfRI* Reverse 5′- CACGCA CTGGAAGTGTGTCT –3′	This paper	N/A
Real-time PCR primer: *TnfRII* Forward 5′- TACCAA GGGTGGCATCTCTC-3′	This paper	N/A
Real-time PCR primer: *TnfRII* Reverse 5′- TCCTGG GATTTCTCATCAGG –3′	This paper	N/A
Recombinant DNA		
pEGFP-LC3 plasmid	Gift from Dr T Yoshimori (Osaka University, Japan) (Kabeya et al., 2000)	N/A
Software and algorithms		
FlowJo v10.2	Tree Star	https://www.flowjo.com/
ImageJ v2.0	NIH	https://imagej.nih.gov/ij/
Imaris v9.2	Bitplane	https://imaris.oxinst.com/
Prism v8.4	GraphPad	https://www.graphpad.com/scientific-software/prism/
IMOD	University of Colorado	https://bio3d.colorado.edu/imod/
Other		
123cound eBeads counting beads	Thermo Fisher Scientific	Cat#01-1234-42
Staurosporine	Abcam	Cat#120056

RESOURCE AVAILABILITY

Lead contact

Further information and requests for resources and reagents should be directed to and will be fulfilled by the lead contact, Sussan Nourshargh (s.nourshargh@qmul.ac.uk).

Materials availability

The supply of the following reagents and mice are subject to MTA agreements with the academics indicated in parenthesis: *GFP-Map1lc3*$^{TG/+}$ mice and *Atg5*$^{fl/fl}$ mice (Prof. Noboru Mizushima); *Cdh5-cre;Atg5*$^{fl/fl}$ mice (Dr. Chantal Boulanger); *Cdh5-creER*^{T2} mice (Prof. Yoshiaki Kubota); *Atg16L1*^{E230} mice (Prof. Tom Wileman).

Data and code availability

This study did not generate or analyze large datasets or codes.

CellPress
OPEN ACCESS

EXPERIMENTAL MODEL AND SUBJECT DETAILS

Mice

All mice were on a C57BL/6 background with the exception of *Atg16l1*$^{E230/E230}$ mice, which were on a mixed sv129 and C57BL/6 background. All animal procedures were performed using 8-12-week-old mice (age and sex matched groups) in accordance with the institutional Animal Welfare Ethical Review Body (AWERB) and UK Home Office guidelines. For some experiments, mice were starved for 24 h with access to water *ad libitum*. *GFP-Map1lc3*$^{TG/+}$ mice were provided by Prof. Mizushima (RIKEN BRC through the National Bio-Resource Project of the MEXT, Japan) (Mizushima et al., 2004). Mice constitutively deficient in EC *Atg5* were obtained by crossing *Cdh5-cre* transgenic mice with *Atg5*$^{fl/fl}$ mice provided by Prof. N. Mizushima (Hara et al., 2006) to create *Cdh5-cre;Atg5*$^{fl/fl}$ mice (Vion et al., 2017). In order to assess the deletion efficiency of the *Cdh5-cre* transgene, *Cdh5-cre;Atg5*$^{fl/fl}$ were intercrossed with *Rosa26*$^{CAG\text{-}loxP\text{-}STOP\text{-}loxP\text{-}tdTomato/\ CAG\text{-}loxP\text{-}STOP\text{-}loxP\text{-}tdTomato}$ reporter mice (*Rosa26*$^{tdTomato/tdTomato}$) (Jackson Laboratory, Stock No: 007905) resulting in *Cdh5-cre;Atg5*$^{fl/fl}$*;Rosa26*$^{tdTomato/+}$ (referred to as *Atg5*$^{\Delta EC}$ in main text) progeny. This mouse line exhibited genetically mosaic populations of ECs comprised of tdTomato^{+} ATG5 null ECs (*Atg*$^{-/-}$, referred to as *Atg*$^{-/-}$ ECs in the main text) neighboring tdTomato^{-}, ATG5 expressing ECs (*Atg5*$^{fl/fl}$, referred to as WT ECs in the main text). *Atg5*$^{\Delta EC}$ mice showed normal circulating leukocyte numbers compared to their littermate controls. Specifically, male *Atg5*$^{\Delta EC}$ and *Atg5*$^{fl/fl}$ mice exhibited 1790 and 1540 neutrophils/μl blood, respectively ($p > 0.7$). Additionally, male *Atg5*$^{\Delta EC}$ and *Atg5*$^{fl/fl}$ mice exhibited 7070 and 7055 lymphocytes/μl blood ($p > 0.9$), 190 and 95 monocytes/μl blood ($p > 0.4$), 150 and 105 eosinophils/μl blood ($p > 0.15$) and 10 and 5 basophils/μl blood ($p > 0.3$), respectively. Because all constitutive EC-Cre deleters are known to be expressed in the embryonic haemogenic endothelium, which thus gives rise to recombined floxed alleles in ECs and hematopoietic lineages, we generated bone marrow chimeric mice (see below) to eliminate any potential impact of myeloid cell *Atg5* deficiency on our experimental readouts. Thus, *Lyz2-EGFP* genetically targeted mice were used as donors for the generation of bone marrow chimeras and were kindly provided by Dr Markus Sperandio (Ludwig Maximilians University of Munich, Germany), used with the permission of Dr Thomas Graf (Center for Genomic Regulation and ICREA, Spain). *Atg5*$^{\Delta EC}$ and *Atg5*$^{fl/fl}$ chimeric animals showed similar peripheral blood neutrophil counts (males: 1271 and 1073 neutrophils/ul of blood ($p > 0.4$); females: 1273 and 1055 neutrophils/ul of blood ($p > 0.4$), respectively) and equivalent reconstitution amounts of EGFP^{+} neutrophils (> 95%). Mice with inducible deletion *Atg5* in ECs (*Atg5*$^{i\Delta EC}$) were obtained by intercrossing the *Cdh5-creER*^{T2} mouse line (*Cdh5(BAC)-creER*^{T2}*; Rosa26*$^{tdTomato/tdTomato}$) (Okabe et al., 2014) with *Atg5*$^{fl/fl}$ mice. 8 days prior to tissue harvesting, *Atg5*$^{iEC\Delta}$ mice and *Atg5*$^{fl/fl}$ littermate controls were subjected to intragastric administration of 100 mL Tamoxifen in corn oil (20 mg/ml) for 2 consecutive days. In *Atg5*$^{i\Delta EC}$ mice, the latter resulted in > 90% recombination of the Rosa allele as determined by tdTomato expression in the cremaster muscle microcirculation, and a ~70% reduction in ATG5 protein and LC3 puncta in lung and cremaster ECs, respectively. *Atg5*$^{i\Delta EC}$ and *Atg5*$^{fl/fl}$ mice showed comparable peripheral blood neutrophil counts after Tamoxifen treatment (1003 and 1112 neutrophils/ul of blood, respectively ($p > 0.6$)). Homozygous *Atg16l1*$^{E230/E230}$ mice (referred to as *Atg16l1*^{E230} in the main text), lacking the WD and linker domain of ATG16L1 (Rai et al., 2019), were provided by Dr Tom Wileman. *Atg16l1*^{E230} mice showed normal circulating leukocyte numbers compared to their littermate controls. Specifically, *Atg16l1*^{E230} and WT mice exhibited 2450 and 1170 neutrophils/μl blood, respectively ($p > 0.4$). Additionally, male *Atg16l1*^{E230} and WT mice exhibited 7287 and 7090 lymphocytes/μl blood, 187 and 95 monocytes/μl blood, 193 and 220 eosinophils/μl blood and 10 and 10 basophils/μl blood, respectively ($p > 0.3$).

Cell culture

Human umbilical vein endothelial cells (HUVECs) isolated from pooled donors (Promocell, Heidelberg, Germany) were grown in M199 (GIBCO-BRL, Grand Island, NY) with 20% fetal calf serum (Hyclone, Logan, UT), 10 U/ml heparin (Sigma, St. Louis, MO) and 30 μg/ml endothelial cell growth supplement (Sigma). All cells were incubated in plates pre-coated with 1% pig skin gelatin (Sigma-Aldrich) at 37°C in an atmosphere of 5% CO_2/95% air.

METHOD DETAILS

Generation of bone marrow chimeric mice

Mice exhibiting ATG5-deficiency in ECs and littermate control chimeras were generated by transferring bone marrow cells from *Lyz2-EGFP* genetically targeted mice into *Atg5*$^{\Delta EC}$ and *Atg5*$^{fl/fl}$ mice respectively. To establish the effect of LAP deficiency in ECs, mice exhibiting LAP sufficiency specifically in the hematopoietic system were established by transferring bone marrow cells from *Lyz2-EGFP* genetically targeted mice into *Atg16l1*$^{E230/E230}$ recipients. For the generation of bone marrow chimeras, 6-8-week-old recipient mice were lethally irradiated with two doses of 5 Gy of ionizing radiation 4 h apart. After 24 h, these mice were intravenously (i.v.) injected with 1.5×10^6 donor cells and reconstitution of neutrophil pools was assessed 4 weeks later by flow cytometry. Validation experiments confirmed that *Atg5*$^{\Delta EC}$ chimeras and *Atg16l1*^{E230} chimeras showed normal circulating neutrophil numbers compared to their littermate control chimeras. Specifically, female *Atg5*$^{\Delta EC}$ and *Atg5*$^{fl/fl}$ mice exhibited 1055 and 1273 neutrophils/μl blood, respectively. Male *Atg5*$^{\Delta EC}$ and *Atg5*$^{fl/fl}$ mice exhibited 1073 and 1271 neutrophils/μl blood, respectively. Finally, male *Atg16l1*^{E230} and WT mice showed 1511 and 1677 neutrophils/μl blood, respectively ($p > 0.5$, n = 6-52 mice per group).

Inflammatory responses in the cremaster muscle

Mice were anaesthetized with 3% isoflurane and cremasteric ischemia-reperfusion (IR) injury was induced by placing an orthodontic elastic band (1/8'') around the anterior aspect of the testis (40 min) followed by removal of the band, intrascrotal (i.s.) injection of

AF-conjugated non-blocking anti-PECAM-1 mAb (4 μg in 400 μL PBS) and initiation of the reperfusion phase (4h unless otherwise specified). Control sham mice underwent placement of the orthodontic band around the testis followed by its immediate removal. For some experiments, anti-PECAM-1 blocking mAb (1 mg/kg) or an isotype control mAb were injected i.v. 30 min prior to the induction of ischemia. In other experiments, mice were subjected to cremasteric IR injury and two hours prior to culling, were injected i.v. with Crimson (625-645) 20 nm microspheres (0.8 μl/g body weight) and non-blocking anti-PECAM-1 mAb to assess vascular permeability and label the vasculature, respectively. Alternatively, mice were subjected to sham or cremasteric IR injury and 3 μg of scrambled control or Tat-Beclin 1 peptide were locally injected (i.s.) together with non-blocking anti-PECAM-1 mAb at 0h or 2 h post-reperfusion, and tissues were harvested 2 h later for analysis. These doses had no impact on local neutrophil extravasation at baseline (Figure 2H). Furthermore, no increase in LC3 puncta was observed in the ear skin vasculature in scrambled versus Tat-Beclin 1 treated animals (0.2 versus 0.18 puncta / um^2 EC x 10^4; respectively), showing that the autophagy-inducing peptide elicited a localized effect and did not impact EC autophagy in distant vascular beds. Alternatively, anaesthetized mice were locally injected (i.s.) with LPS (Sigma-Aldrich, 300 ng in 400 μL PBS) or PBS together with fluorescently conjugated non-blocking anti-PECAM-1 mAbs i.s. for 4 h. At the end of experiments, mice were culled by Schedule one method (cervical dislocation) and tissues were collected for IF staining. To induce apoptosis, mice were anaesthetized with 3% isoflurane and injected locally (i.s.) with Staurosporine (Abcam, 1 μM in 400 μL PBS) together with fluorescently conjugated non-blocking anti-PECAM-1 mAb for 4 h.

Cell transfection

Plasmid and siRNA transfections were performed by nucleofection (Nucleofector II, program U-001; Amaxa Biosystems, Gaithersburg, MD, USA) using 2-10 μg DNA or 250 pmol of siRNA.

Immunofluorescence staining

Cremaster muscles were fixed in 4% paraformaldehyde (PFA, Sigma-Aldrich) for 1 h at 4°C and permeabilized and blocked in PBS containing 0.5% Triton X-100 (Sigma-Aldrich) and 25% fetal calf serum (FCS, Thermo Fisher Scientific) for 4 h at room temperature. Subsequently, the tissues were incubated with fluorescently labeled primary antibodies in PBS containing 10% FCS over night at 4°C.

For *in vitro* imaging, HUVECs were seeded on #1.5 thickness coverslips (0.17 mm) (VWR) and/or polymer coverslip bottom dishes (Ibidi) and grown to confluence. Next, unstimulated or LPS-treated (1 μg/ml for 4 h) HUVECs were fixed with 4% PFA for 20 min, permeabilized for 5 min with PBS containing 0.2% Triton X-100 at room temperature, blocked with PBS containing 3% Bovine Serum Albumin (BSA, Sigma-Aldrich) for 15 min and incubated at 4°C with primary antibodies over-night. After extensive washes, cells were incubated with appropriate fluorophore-conjugated secondary antibodies.

Confocal image acquisition of fixed samples

Leica TCS SP8 and Zeiss LSM800 laser scanning confocal microscopes were used to capture serial optical z sections of cultured HUVECs and postcapillary venules and arterioles (diameter 20-45 μm) in immunostained whole-mounted cremaster muscles. Images were acquired with 20x/1.0 water, 40x/1.3 oil or 63x/1.4 oil objective lenses, with a 0.07-0.62 μm pixel size and 0.23-1.0 μm voxel depth as required. Where colocalization analyses were required, all channels were acquired sequentially and pinhole diameter was adjusted to retain optical section thickness of 555 ± 62 nm.

Confocal super-resolution imaging

Fixed HUVECs overexpressing GFP-LC3 were imaged with an inverted Nikon CSU-W1 SoRa spinning disk confocal microscope, equipped with a 100x/1.35 silicon objective lens and Prime 95B sCMOS camera (Photometrics). Images were acquired with the SoRa disk and appropriate magnifier to produce an effective pixel size of 0.04 μm. Serial z stacks were captured with a 0.15-1.0 μm interval and subsequently deconvolved with a maximum likelihood estimation method.

Correlative light electron microscopy

Region of interest (ROI) targeting

The tissue was carefully removed from the glass slide in phosphate buffer (PB) and immediately fixed with 2.5% glutaraldehyde and 4% paraformaldehyde in PB for 1h. The sample was then post-fixed and stained in a Pelco BioWave Pro+ microwave (Ted Pella) using an adapted version of the NCMIR protocol (Deerinck et al., 2010). Briefly, the tissue was post-fixed in 2% osmium tetroxide and 1.5% potassium ferricyanide, incubated in 1% thiocarbohydrazide, followed by 2% osmium tetroxide. Osmicated tissue was then stained en bloc with 1% uranyl, followed by Walton's lead aspartate at 60°C. It was then dehydrated with a graded ethanol series, flat-embedded in Durcupan ACM® resin, and polymerized at 60°C.

Position of the ROI was identified in the embedded tissue by X-ray micro-computed tomography (micro-CT) using an XRadia 510 Versa (Zeiss X-Ray Microscopy) guided by tissue shape and venules as landmarks. The tissue was trimmed to the area that includes the landmarks and was mounted onto an aluminum pin using conductive epoxy glue (ITW Chemtronics). A second micro-CT scan was performed and the tissue was trimmed around the ROI. The final SBF-SEM block was micro-CT scanned at 1.03 μm/pixel resolution which revealed cell nuclei. tdTomato signal of the 63 × tile scan stack was matched to the tomographic slices using the Fiji plugin BigWarp (Bogovic et al., 2016) based on nuclei positions as landmarks. Positions of the *Atg5*$^{-/-}$ cells were identified on the

CellPress
OPEN ACCESS

block face and depth of the tissue was estimated from the tomographic slices to target for SBF-SEM. All micro-CT data were handled in the 3dmod program of the IMOD software package (Kremer et al., 1996).

Serial block face scanning electron microscopy (SBF-SEM)

Prior to SBF-SEM, the block was sputter-coated with 5–10 nm platinum using a Q150R S sputter coater (Quorum Technologies). SBF-SEM data was collected using a 3View2XP (Gatan Inc.) attached to a Sigma VP SEM (Zeiss). The microscope was operated at 1.8kV with 30-μm aperture, using Focal Charge Compensation mode (Deerinck et al., 2018). Inverted backscattered electron images were acquired every 50 nm, at a resolution of 8.44 nm/pixel. Acquired images were imported into Fiji (Schindelin et al., 2012) and aligned using the Register Virtual Stack Slices (Arganda-Carreras et al., 2006) and in TrakEM2 using manual landmarks (Cardona et al., 2012). 63 × Z stack was matched to the aligned SBF-SEM data using BigWarp (Bogovic et al., 2016) based on nuclei positions and overlaid to the EM data after applying thin-plate spline transformation. Cells in the region of expanded cell-cell contacts were selected and segmented using the TrakEM2. Cell-cell contacts were segmented if the plasma membranes of two neighboring cells were closely apposed with spacing less than or equal to 25 nm based on the length of homo dimer of PECAM extracellular domains (Jiang et al., 2016). 3D reconstructions were made in the 3dmod program.

Confocal intravital microscopy (IVM)

The mode and dynamics of neutrophil migration through blood vessel walls was analyzed by confocal IVM as described previously (Woodfin et al., 2011). In order to analyze neutrophil interactions with ECs, chimeric *Cdh5-cre;Atg5*$^{fl/fl}$*;Rosa26*$^{tdTomato/+}$ male mice were injected i.s. with an AF-647 labeled non-blocking anti-PECAM-1 mAb to stain EC junctions. 2 h after i.s. injection, mice were anaesthetized by intraperitoneal (i.p.) administration of ketamine (100 mg/kg) and xylazine (10 mg/kg) and cremaster muscles were exteriorized and pinned out flat over the optical window of a heated custom-built microscope stage. Animals were maintained at 37°C and cremaster muscles were perfused with warm Tyrode's solution (Sigma-Aldrich) throughout the experiment. 2 h after i.s. injection, mice were subjected to IR injury by placing a clamp at the base of the exteriorized tissue for 40 min to induce ischemia, after which the clamp was removed to allow reperfusion over a 2 h period. Control sham operated mice underwent identical surgical procedures but without inducing tissue IR. For some experiments, anaesthetized mice received an i.s. injection of fluorescently labeled anti-PECAM-1 mAb alone or co-injected with LPS (300 ng) to label blood vessels and/or induce an inflammatory response, respectively. Alternatively, exteriorised cremaster muscle tissues of sham or IR-treated mice were incubated with 2 μM propidium iodide during 5 minutes prior to imaging acquisition.

Postcapillary venules (20-45 μm diameter) of flat mounted cremaster muscles were imaged every 60 s for 0.5-2.0 h using an upright Leica SP5 confocal laser scanning microscope equipped with a 20x/1.0 water-dipping objective lens and 8 kHz resonant scanner. Resultant time-lapse z stack images were assembled offline into 3D videos using Imaris software. The x × y × z dimensions of the recorded area were typically 300 × 130 × 35 μm and the resulting voxel size was 0.29 × 0.29 × 0.69 μm.

Time-lapse fluorescence imaging

HUVECs expressing GFP-LC3 were seeded onto 8 well μ-slides (Ibidi) and grown for 24-48 h. Cells were then stimulated with LPS (1 μg/ml) for 4 h and AF-Fluor 555 non-blocking anti-PECAM-1 mAb (2 μg/ml) was added for 5-15 min prior to imaging using an inverted Zeiss LSM800 confocal laser scanning microscope. Cells were imaged at 30 s intervals for a maximum of 1 h in growth medium containing 20 mM HEPES, in a heated chamber at 37°C. Images were analyzed using Imaris software.

In vitro neutrophil TEM assay

TEM of human neutrophils through HUVECs was quantified as previously reported (Muller and Luscinskas, 2008; Muller et al., 1993). Briefly, HUVEC monolayers grown to confluence on hydrated collagen gels were treated for 4 h with bafilomycin A1 (100 nM) or diluent control in complete culture media (Medium 199 + 20% heat-inactivated adult human serum). Neutrophils, collected from healthy adult donors, were isolated by density gradient centrifugation at room temperature without red blood cell (RBC) lysis. HUVEC monolayers were washed 3 times in Medium 199 + 0.1% human serum albumin. Neutrophils were resuspended to 1 × 10^6 per ml in the same medium containing 20 μg/ml rabbit anti-PECAM IgG or preimmune IgG from the same rabbit. The cells were allowed to transmigrate for 2.5 minutes, washed in saline and fixed in 10% neutral buffered formalin prior to staining and quantitation of TEM.

Peritoneal inflammation

Peritonitis was induced during 4h (unless otherwise indicated) by i.p. injection of LPS (1 mg/kg), zymosan A (Sigma-Aldrich, 1 mg) or MDP (300 μg) in 500 μl of PBS, with control mice receiving PBS only. Mice were then culled and subjected to peritoneal lavage using 5 mL PBS containing 5 mM EDTA (Sigma-Aldrich) and 0.25% BSA. The resultant peritoneal exudates were immunostained with fluorescently labeled primary antibodies targeting proteins of interest to quantify the total numbers of infiltrating neutrophils by flow cytometry (Live $CD45^+CD11b^+SiglecF^-Ly6G^+F4/80^-$), monocytes (Live $CD45^+CD11b^+SiglecF^-Ly6G^-Ly6C^{hi}F4/80^{low}$) and eosinophils (Live $CD45^+CD11b^+SiglecF^+$), per peritoneal cavity.

Cutaneous inflammation

LPS (300 ng in 50 μl PBS, IL1β [R&D], 50 ng in 50 μl PBS) or PBS alone were injected intradermally into shaved dorsal skin of chimeric mice. After 4 h, skin samples were dissected, frozen in liquid nitrogen and homogenized in homogenization buffer (600 mM NaCl, 0.5% HTAB buffer, 600 mM KH_2PO_4, 66 mM Na_2HPO_4) using a Precellys-24 tissue homogenizer (Bertin Technologies). Tissue debris

was removed by centrifugation and the supernatants assayed for peroxidase activity, determined by adding the MPO substrate 3,3′,5,5′-tetramethylbenzidine (Invitrogen) and measuring optical absorbance at 650 nm at 1 min intervals over 20 min using a Spectra MR microplate spectrophotometer (Dynex Technologies). MPO activity (used as a readout for neutrophil tissue infiltration) was expressed as the increase in optical density per min multiplied by 10.

Mouse lung endothelial cell (MLEC) isolation

Lungs from *Cdh5-cre;Atg5*$^{fl/fl}$*;Rosa26*$^{tdTomato/+}$ control mice or mice subjected to a model of endotoxemia (i.p. injection of LPS from *Escherichia coli* [6 mg/kg, Sigma-Aldrich] and Peptidoglycan G from *Staphylococcus aureus* [1 mg/kg, Sigma-Aldrich] [LPS + PG]), were digested in 0.1% type I collagenase (GIBCO) for 40 min at 37°C. Homogenates were passed through an 18G needle followed by centrifugation, and RBC lysis of the pellet using ACK lysis buffer (150 mM NH_3Cl, 1 mM $KHCO_3$ and 1 mM EDTA) at room temperature for 5 min. Samples were then filtered and washed before immunostaining and cell sorting as detailed below.

Flow cytometry and cell sorting

Where required, blood samples were treated with ACK lysis buffer. All samples were incubated with anti CD16/-CD32 antibodies (Becton Dickinson) to block Fc-receptors and stained with fluorescently labeled primary antibodies of interest. The samples were analyzed on an LSR Fortessa flow cytometer (Becton Dickinson) and FlowJo software (TreeStar). Total cell counts were determined using fluorescent counting beads (Thermo Fisher Scientific). Freshly isolated MLECs from *Cdh5-cre;Atg5*$^{fl/fl}$*;Rosa26*$^{tdTomato/+}$ mice were sorted on a BD FACSAria II cell sorter as CD45$^-$PECAM-1$^+$CD102$^+$tdTomato$^-$ (WT ECs) and CD45$^-$PECAM-1$^+$CD102$^+$tdTomato$^+$ (*Atg5*$^{-/-}$ ECs).

Real Time qRT-PCR analysis

Total RNA was purified from isolated MLECs using the RNeasy micro kit (QIAGEN) and reverse transcribed into cDNA with the iScript cDNA synthesis kit (Bio-Rad). Quantitative real-time PCR was carried out using the iQ SYBR Green Supermix (Bio-Rad or Thermofisher Scientific) according to the manufacturer's instructions, primers for *Pecam-1*, *Cdh5*, , *Icam-1, Icam-2, Vcam-1, E-selectin, Cxcl1, TnfRI, TnfRII Atg5*, *TdtTomato* and *Gapdh* (Integrated DNA Techonologies) and the 7900HT Fast Real-Time PCR system (Thermo Fisher Scientific). All transcript were quantified relative to *Gapdh*.

Remote organ vascular leakage quantification

In some experiments, remote organ vascular permeability was quantified by accumulation of the plasma protein tracer Evans Blue (EB, Sigma-Aldrich). Here, filtered 1% Evans Blue solution was injected i.v. (5 μl/g body weight) 30 mins prior to sacrifice by exsanguination followed by a whole-body vascular washout using 10 mL PBS. Lungs were collected and tissue accumulated dye was quantified by eluting in 300 μl of formamide for 18 h at 56°C. Optical density (OD) readings were taken at 620 nm nm and used as a measure of vascular leakage.

Cell surface biotinylation assays

Cultured HUVECs were stimulated with LPS (1 μg/ml) for 4 h prior to labeling cell surface-associated proteins with EZ-Link sulfo-NHS-biotin (Thermo Scientific). Briefly, HUVEC monolayers plated on 60 mm dishes were incubated with a solution of 0.1 mg/ml of sulfo-NHS-biotin in PBS supplemented with 1.5 mM Ca^{2+} for 30 min at 4°C to block membrane trafficking events such as internalisation of cell surface proteins, during the biotinylation reaction. Excess sulfo-NHS-biotin was then removed by washing with cold PBS supplemented with 1.5 mM Ca^{2+} and cells were incubated with cold DMEM supplemented with 10% FBS to quench any remaining free reagent. Following another wash, cells were either directly lysed *in situ* or, for pulse-chase experiments, cultured at 37°C for the indicated times and then lysed as above. Lysates were prepared by passing cell homogenates through a 0.6 mm, 24G needle and then centrifuged at 14,000 rpm for 5 min to pellet insoluble debris. Part of the supernatant was conserved as total lysate followed by pull-down of biotinylated proteins with NeutrAvidin agarose (Thermo Scientific).

Immunoblotting

Cell lysates were prepared in 2X Laemmli sample buffer (Bio-Rad) containing protease and phosphatase inhibitors (Roche) and denatured at 95°C for 5 min. Samples were run on 10 or 15% SDS-PAGE gels and transferred onto PVDF membranes (GE Healthcare) using standard techniques. Immunoblots were blocked in PBS containing 5% non-fat milk and 0.1% Tween 20 at room temperature for 1 h and then incubated with primary antibodies overnight at 4°C. After extensive washes, immunoblots were incubated with horseradish peroxidase-conjugated secondary antibodies (Dako), and proteins were visualized by enhanced chemiluminescence (Radiance Plus, Azure) acquired on Azure Biosystems Imaging System (Cambridge Bioscience).

QUANTIFICATION AND STATISTICAL ANALYSIS

Quantification of confocal IVM videos

Models of longitudinal half vessel projections were generated to unequivocally identify individual neutrophils migrating through the different compartments of the venular walls. Different modes of neutrophil TEM (paracellular and transcellular) and their dynamics were determined by manual tracking of individual neutrophils using Imaris software. Luminal neutrophils were defined as adherent

CellPress
OPEN ACCESS

when they remained stationary on the endothelium for at least 60 s. Neutrophil TEM was defined as an event where neutrophils fully migrated through ECs in a luminal-to-abluminal direction. The duration of TEM events was calculated as the time between the image frame in which the neutrophil was first observed to disrupt immunolabelled PECAM-1 regions of ECs up to the image frame in which the neutrophil had fully traversed the EC barrier. Paracellular and transcellular TEM were defined as the percentage of total TEM events whereby neutrophils migrated through EC junctions or directly through the EC body, respectively. TEM through ''hot spots'' was defined as any instance where 2 or more neutrophils were observed migrating through the same EC pore/junction. Frequency of such 'hotspot' TEM events was quantified as the percentage of total TEM events observed in each experiment. The number of PECAM-1 pores was quantified by manually counting the total number of single PECAM-1 immunolabeled pores that were observed throughout the entire imaging acquisition period. The duration of PECAM-1 pore 'opening time' was defined as the time between the first image frame in which PECAM-1 labeling was disrupted to the image frame in which the pore fully closed. Of note, PECAM-1 pores that remained open throughout the total experimental time frame were excluded from this analysis.

Image quantification of postcapillary venules in fixed cremaster muscle

Image stacks of longitudinal half vessels were reconstructed in 3D and analyzed using Imaris software (Bitplane). ECs exhibiting GFP-LC3 puncta were quantified as cells presenting at least one GFP-LC3-positive punctum. Individual ECs and neutrophils in the field of view were counted manually and data expressed as percentage of cells expressing puncta. The number of puncta per unit area of EC was quantified using the Imaris software 'spots function' (the 'estimated diameter' function for identifying puncta was set to 0.5 μm), and was normalized to the total surface area of individual ECs based on the 'isosurfaces' masking of PECAM-1 positive areas (i.e., PECAM-1^{high} junctional and PECAM-1^{low} non-junctional regions).

Extravasated neutrophils per field of view (330 × 160 × 45 μm) were defined as those that had fully transmigrated through both the EC and pericyte layers, identified by characteristic morphological changes to the neutrophil cell body, and were quantified from 8-10 images per mouse.

Thickened EC junctions defined as having a width of > 2 μm were determined using the measuring tool of Imaris software to specify EC-cell contact regions identified by PECAM-1 or VE-Cadherin immunostaining. The frequency of thickened junctions per EC was quantified based on the isosurface masks made on PECAM-1 positive areas (PECAM-1^{high} junctional and PECAM-1^{low} non-junctional regions). Fluorescence intensity line profiles along EC-cell contacts (as visualized by PECAM-1 or VE-Cadherin immunostaining) were determined using ImageJ software (NIH) and were used to quantify EC junction width (μm) and junctional molecule enrichment (fluorescence sum intensity) at cell-cell contact sites. 5-8 postcapillary venules were quantified per mouse.

Quantification of fixed HUVEC images

Images were smoothed with a Gaussian filter and background signal removed using the ImageJ 'Background Subtraction' function. Manual thresholding was carried out on each channel by visual inspection. Signal colocalization was quantified using the Manders colocalization coefficient, implemented using the ImageJ JACoP plugin (Bolte and Cordelières, 2006). Where biological control was not possible, the analysis was repeated after 90º rotational offset of one channel with respect to the other, providing an internal control (referred to as offset control in main text). The number of GFP-LC3 vesicles co-labeled with PECAM-1 and/or WIPI2 were determined using the ImageJ 3D Objects Counter plugin (Bolte and Cordelières, 2006).Following image pre-processing, binary 'co-localization' channels were derived from the intersection of the individual channel thresholds. Puncta with a minimum 50 voxel overlap were counted, in addition to the total GFP-LC3 puncta number.

Statistics

Statistical analyses were performed using Prism 8.0 software (GraphPad) and datasets expressed as mean ± SEM with n numbers of 3 or more biological replicates as detailed in Figure legends. Sample sizes were chosen on the basis of published work in which similar experiments had been reported. Comparisons between two groups were carried out using the paired or unpaired Student's t test. One-way ANOVA or two-way ANOVA with Tukey's or Bonferroni's post hoc test were performed for multiple group comparisons. Statistical significance was accepted at $p < 0.05$.

Immunity, Volume *54*

Supplemental information

Autophagy modulates endothelial junctions to restrain neutrophil diapedesis during inflammation

Natalia Reglero-Real, Lorena Pérez-Gutiérrez, Azumi Yoshimura, Loïc Rolas, José Garrido-Mesa, Anna Barkaway, Catherine Pickworth, Rebeca S. Saleeb, Maria Gonzalez-Nuñez, Shani N. Austin-Williams, Dianne Cooper, Laura Vázquez-Martínez, Tao Fu, Giulia De Rossi, Matthew Golding, Mathieu-Benoit Voisin, Chantal M. Boulanger, Yoshiaki Kubota, William A. Muller, Sharon A. Tooze, Thomas D. Nightingale, Lucy Collinson, Mauro Perretti, Ezra Aksoy, and Sussan Nourshargh

Figure S1

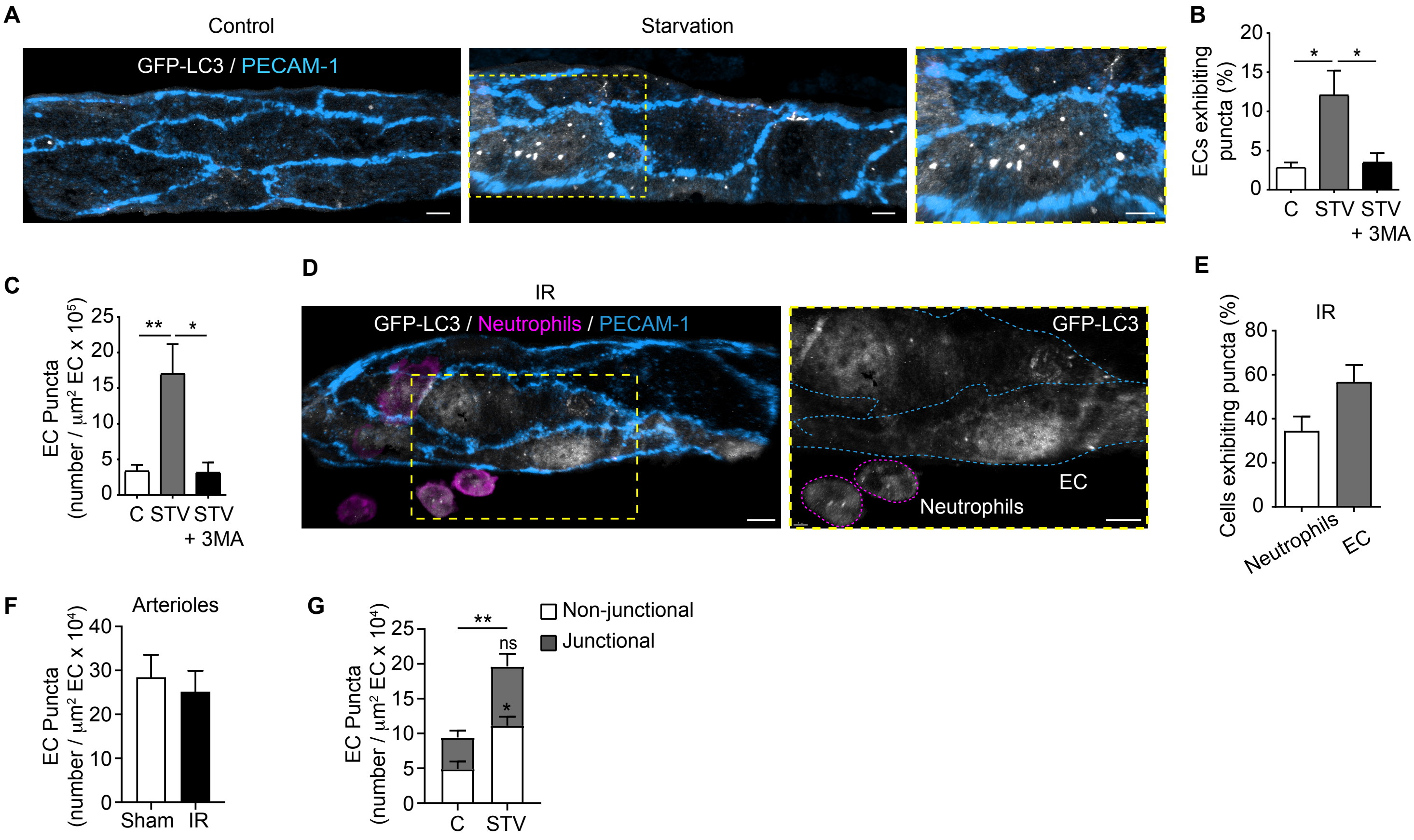

Figure S1 (Related to Figure 1). ECs exhibit GFP-LC3 puncta *in vivo* in response to starvation and inflammation. (**A-C**) *GFP-Map1lc3a*$^{TG/+}$ mice were starved for 24 h. (**A**) Representative (n=7) confocal images of post-capillary venules (PCVs, PECAM-1) (scale bar 5 µm). (**B**) Percentage of venular ECs exhibiting puncta and (**C**) number of puncta per EC area in control and 3-MA-treated tissues, as quantified by confocal microscopy (n= 4-7 mice/group). (**D-F**) *GFP-Map1lc3a*$^{TG/+}$ mice were subjected to local IR injury. (**D**) Representative confocal images (n=4) of PCVs (PECAM-1) stained for neutrophils (MRP14) (scale bar 5 µm). (**E**) Percentage of neutrophils and ECs exhibiting puncta (n=3-4 mice/group) and (**F**) quantification of GFP-LC3 puncta per arteriolar EC area, as assessed by confocal microscopy (n=3-6 mice/group). (**G**) *GFP-Map1lc3a*$^{TG/+}$ mice were starved for 24 h and cremasteric PCVs analyzed for GFP-LC3 puncta localization by confocal microscopy (n=4-7 mice/group). Dashed boxes delineate magnified areas. Means ± SEM. Statistically significant difference from controls is shown by *P< 0.05 and **P< 0.01; ns, not significant.

Figure S2

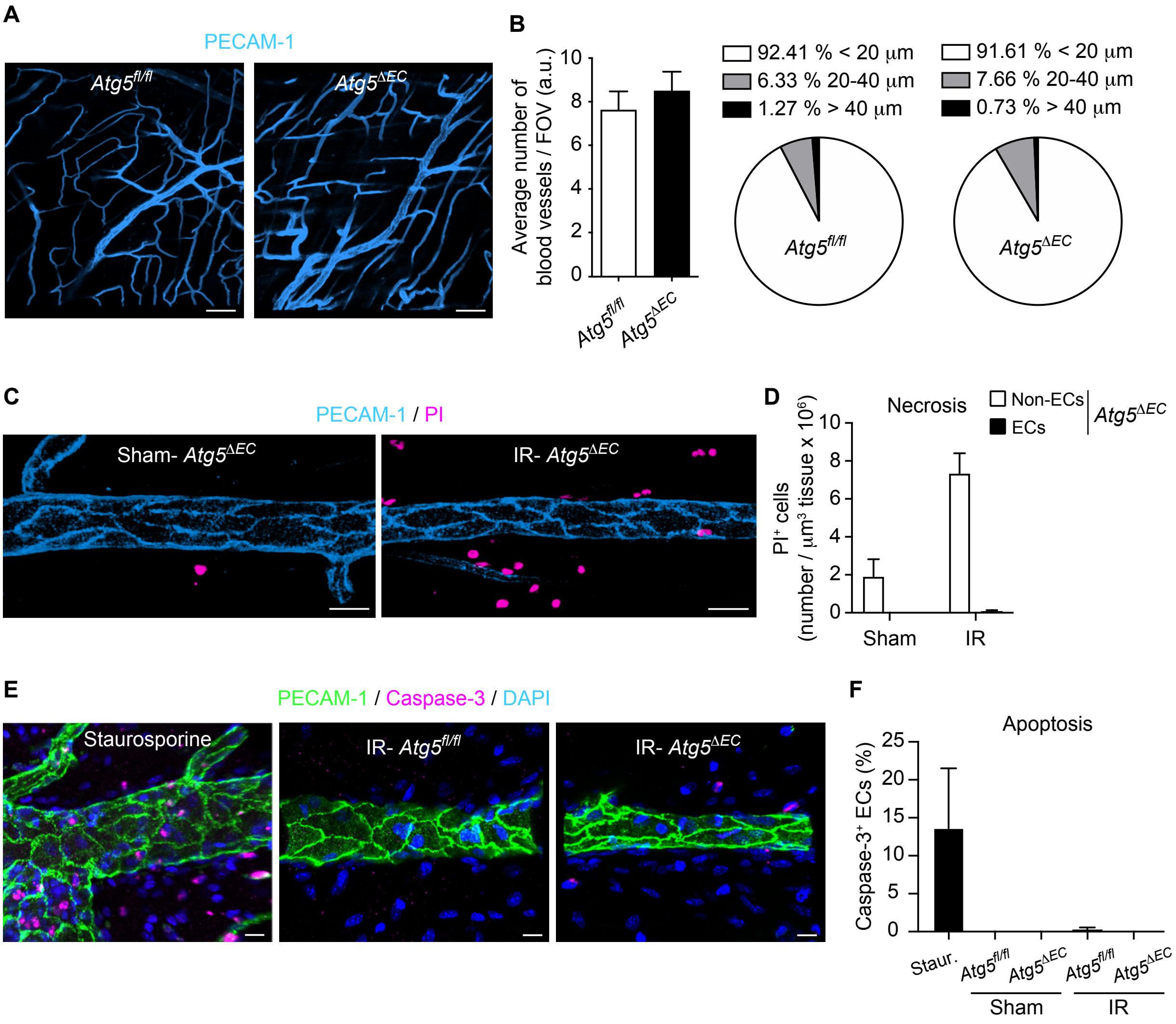

Figure S2 (Related to Figure 2). ATG5 deficient ECs are viable and healthy *in vivo*. (**A**) Representative confocal images (n=4) and (**B**) quantification of the number and size of blood vessels in post-capillary venules (PCVs) of *Atg5$^{fl/fl}$* and *Atg5$^{\Delta EC}$* mice, as quantified by confocal microscopy (n=4 mice/group). (**C-F**) *Atg5$^{fl/fl}$* and *Atg5$^{\Delta EC}$* mice were subjected to local IR injury. (**C**) Representative confocal images (n=4) of PCVs (PECAM-1) stained for propidium iodide (PI) and (**D**) quantification of necrotic cells (PI+) within *Atg5$^{\Delta EC}$* mice, as quantified by confocal IVM (n=4 mice/group). (**E**) Representative confocal images (n=3) of PCVs (PECAM-1) stained for apoptotic cells (Caspase-3, scale bar 15 µm) and (**F**) percentage of Caspase-3 positive ECs, as assessed by confocal microscopy (n=3 mice/group). Means ± SEM.

Figure S3

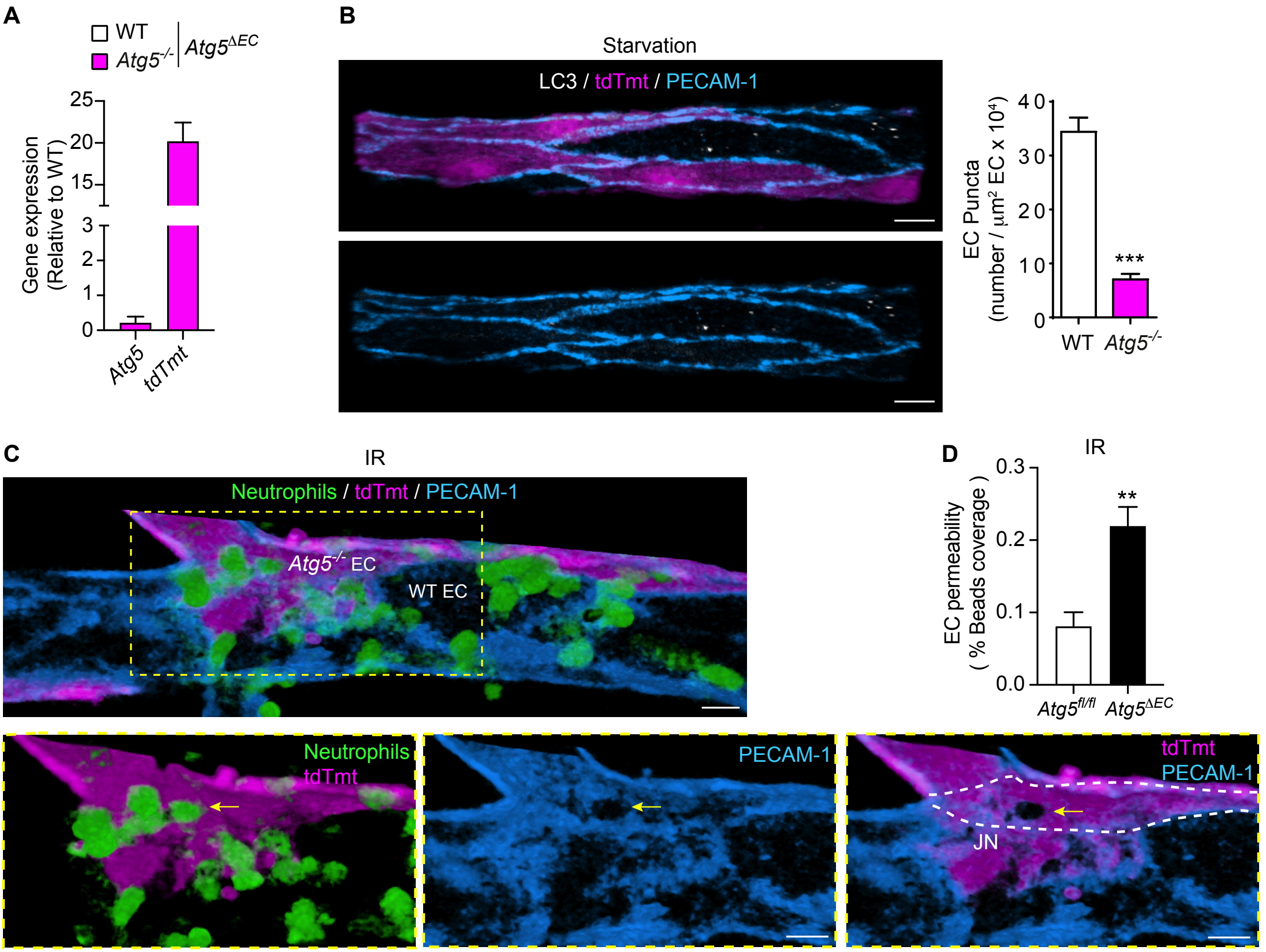

Figure S3 (Related to Figure 3). EC ATG5 deficiency inhibits LC3 puncta formation *in vivo* and supports neutrophil transcellular TEM. (**A**) Lung ECs of *Atg5*$^{\Delta EC}$ mice were analyzed for *Atg5* and *tdTomato* mRNA levels by RT-qPCR (n=3 mice/group). (**B**) Representative confocal images (n=3) of cremasteric post-capillary venules (PCVs) of starved *Atg5*$^{\Delta EC}$ mice stained for endogenous LC3, and associated quantification of LC3 puncta per venular EC area of tdTomato$^-$, wild-type (WT) ECs and tdTomato$^+$, *Atg5*$^{-/-}$ ECs (scale bar 7 μm, n=3 mice/group). (**C-D**) *Atg5*$^{fl/fl}$ and *Atg5*$^{\Delta EC}$ mice were subjected to local IR injury. (**C**) Representative confocal images (n=7) of a PCV supporting neutrophil transcellular TEM as shown through establishment of a pore in the body of an *Atg5*$^{-/-}$ (tdTmt$^+$) EC (arrow, scale bar 10 μm). (**D**) Quantification of EC permeability in *Atg5*$^{fl/fl}$ and *Atg5*$^{\Delta EC}$ mice, as assessed by confocal microscopy (n=3-4 mice/group). Dashed boxes delineate magnified areas. Means ± SEM. Statistically significant difference from controls is shown by **P< 0.01 and ***P< 0.001.

Figure S4

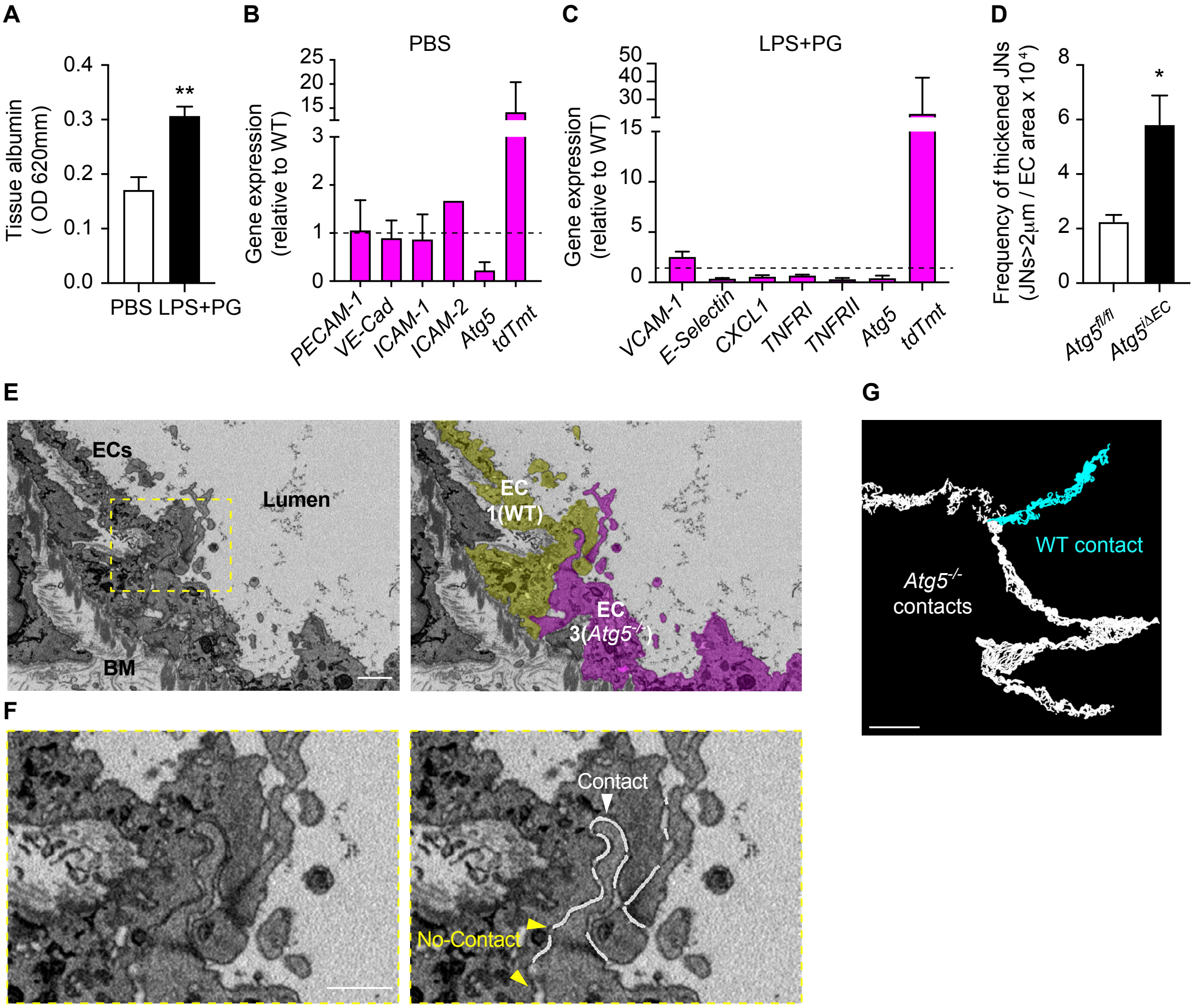

Figure S4 (Related to Figure 4). ATG5-dependent autophagy regulates the architecture and molecular composition of EC contacts. (**A-C**) *Atg5*$^{\Delta EC}$ mice were subjected to PBS or a model of endotoxemia (LPS+PG). (**A**) Quantification of lung vascular leakage, as assessed by local Evans blue extravasation (n=3 mice/group). mRNA levels under (**B**) basal and (**C**) endotoxemia conditions in lung ECs from *Atg5*$^{\Delta EC}$ mice, as assessed by RT-qPCR (n=3-5 mice/group). (**D**) Frequency of thickened junctions in post-capillary venules of IR-stimulated *Atg5*$^{i\Delta EC}$ mice, as quantified by confocal microscopy (n=3-4 mice/group). (**E-G**) One *Atg5*$^{\Delta EC}$ mouse was subjected to local IR injury. Electron micrographs of WT and *Atg5*$^{-/-}$ ECs shown in Figure 4J illustrating segmentation of (**E**) ECs and (**F**) cell-cell contacts (scale bars 1 μm and 0.5 μm, respectively). (**G**) 3D reconstruction of segmented cell-cell contacts following SBF-SEM of ECs depicted in Figure 4K (scale bar 5 μm). Dashed box delineates magnified area. Means ± SEM. Statistically significant difference from controls or between indicated groups is shown by *P< 0.05 and **P<0.01. BM, Basement membrane.

Figure S5

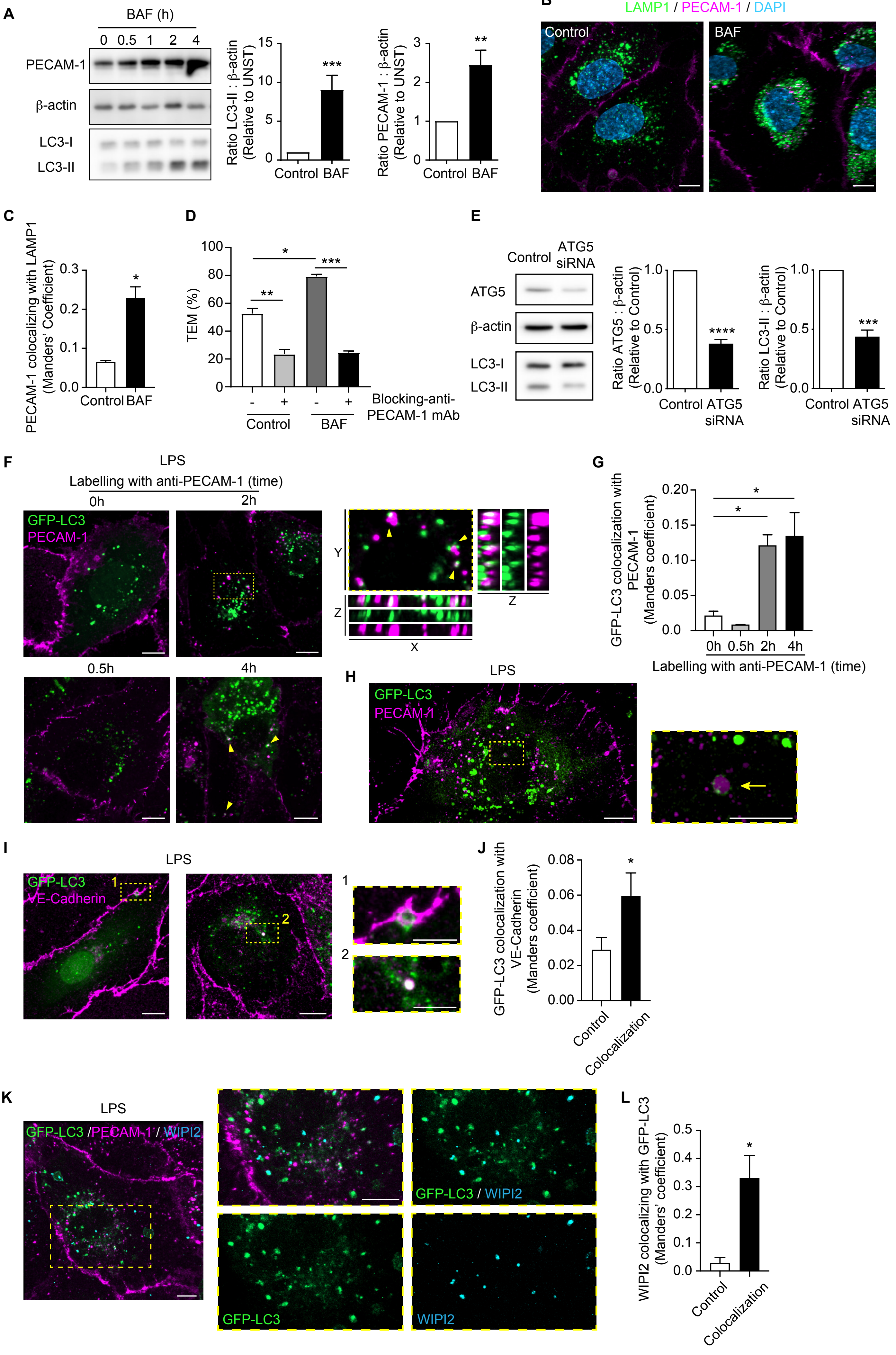
A
BAF (h)
0 0.5 1 2 4
PECAM-1
β-actin
LC3-I
LC3-II
Ratio LC3-II : β-actin (Relative to UNST)
Ratio PECAM-1 : β-actin (Relative to UNST)
Control BAF
B
LAMP1 / PECAM-1 / DAPI
Control
BAF
C
PECAM-1 colocalizing with LAMP1 (Manders' Coefficient)
Control BAF
D
TEM (%)
Blocking-anti-PECAM-1 mAb
Control
BAF
E
Control
ATG5 siRNA
ATG5
β-actin
LC3-I
LC3-II
Ratio ATG5 : β-actin (Relative to Control)
Ratio LC3-II : β-actin (Relative to Control)
F
LPS
Labelling with anti-PECAM-1 (time)
0h
2h
0.5h
4h
GFP-LC3
PECAM-1
G
GFP-LC3 colocalization with PECAM-1 (Manders coefficient)
0h 0.5h 2h 4h
Labelling with anti-PECAM-1 (time)
H
LPS
GFP-LC3
PECAM-1
I
LPS
GFP-LC3
VE-Cadherin
J
GFP-LC3 colocalization with VE-Cadherin (Manders coefficient)
Control
Colocalization
K
LPS
GFP-LC3 / PECAM-1 / WIPI2
GFP-LC3 / WIPI2
GFP-LC3
WIPI2
L
WIPI2 colocalizing with GFP-LC3 (Manders' coefficient)
Control
Colocalization

Figure S5 (Related to Figure 5). Bafilomycin A1 increases PECAM-1 protein levels and supports enhanced neutrophil TEM and internalized surface PECAM-1 localizes to GFP-LC3 positive compartments. (**A-D**) HUVECs were treated with bafilomycin A1 (BAF, 100 nM) for 4 h or the indicated times. (**A**) Immunoblot of total PECAM-1, LC3-I and LC3-II with β-actin acting as loading protein control and associated quantifications of fold increase of LC3-II and PECAM-1 relative to unstimulated conditions (n=11). (**B**) Representative confocal images (n=3) of HUVECs immunostained for PECAM-1 and LAMP1 and (**C**) associated quantification of PECAM-1 colocalization with LAMP1 (n=3, scale bars 10 μm). (**D**) Neutrophil TEM in the presence or absence of a blocking anti-PECAM-1 mAb (n=3). (**E**) Immunoblot of total ATG5 and LC3-II with β-actin acting as loading protein control for ATG5 silenced and control HUVECs and associated quantifications showing ATG5 and LC3-II fold change (n=6). (**F-L**) GFP-LC3 transfected HUVECs were stimulated with LPS for 4 h. (**F**) Representative confocal images (n=3-5) of GFP-LC3$^+$/ PECAM-1$^+$ vesicles at the indicated times after incubation with anti-PECAM-1 mAb by antibody feeding (scale bars 10 μm); and (**G**) associated quantification of GFP-LC3 colocalization with PECAM-1, as quantified by Manders' coefficient (n=3-5; 40-100 cells analyzed per condition). (**H**) Representative super-resolution confocal image (n=1) of a GFP-LC3-associated vesicle containing PECAM-1 (arrow, scale bars 10 μm). (**I**) Representative confocal images (n=4) of GFP-LC3 transfected HUVECs immunostained for VE-Cadherin (scale bars 10 μm and enlargements 5 μm) and (**J**) quantification of GFP-LC3 colocalization with VE-Cadherin as compared to its offset control, as quantified by Manders' coefficient (n=4; >100 cells analyzed). (**K**) Representative confocal images (n=4) of GFP-LC3 transfected HUVECs immunostained for PECAM-1 and WIPI2, showing GFP-LC3$^+$ and WIPI2$^+$ vesicles (scale bar 10 μm) and (**L**) associated quantification of WIPI2 colocalization with GFP-LC3 as compared to its offset control, as quantified by Manders' coefficient (n=4; >100 cells analyzed). Dashed boxes delineate magnified areas. Means ± SEM. Statistically significant difference from controls or between indicated groups is shown by *P< 0.05, **P< 0.01, ***P< 0.001 and ****P<0.0001.

Figure S6

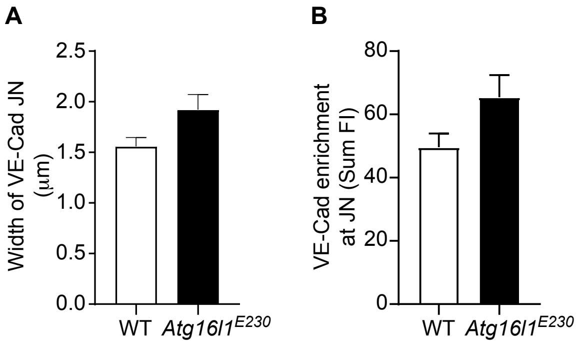

Figure S6 (Related to Figure 6). VE-Cadherin junctional expression in post-capillary venules from *Atg16L1^{E230}* mice. WT and *Atg16L1^{E230}* mice were subjected to local IR injury. Quantification of VE-Cadherin (**A**) junctional width and (**B**) protein enrichment, as assessed by confocal microscopy (n=3 mice/group; 12-19 vessels quantified per condition). Means ± SEM.
